# Efficient data extraction from neutron time-of-flight spin-echo raw data

**DOI:** 10.1107/S1600576719010847

**Published:** 2019-08-29

**Authors:** P. A. Zolnierczuk, O. Holderer, S. Pasini, T. Kozielewski, L. R. Stingaciu, M. Monkenbusch

**Affiliations:** a Forschungszentrum Jülich GmbH, JCNS Outstation, Oak Ridge, Tennessee, USA; b Forschungszentrum Jülich GmbH, JCNS MLZ, Garching, Germany; c Forschungszentrum Jülich GmbH, JCNS-1, Jülich, Germany; d NScD, Oak Ridge National Laboratory, Oak Ridge, TN 37831, USA

**Keywords:** neutron spin echo, NSE, spallation neutron sources, data reduction

## Abstract

Neutron spin-echo spectrometers with a position-sensitive detector and operating with extended time-of-flight-tagged wavelength frames are able to collect a comprehensive set of data covering a large range of wavevector and Fourier time space with only a few instrumental settings in a quasi-continuous way. Extracting all the information contained in the raw data and mapping them to a suitable physical space in the most efficient way is a challenge. This article reports algorithms employed in dedicated software, *DrSpine* (data reduction for spin echo), that achieves this goal and yields reliable representations of the intermediate scattering function *S*(*Q*, *t*) independent of the selected ‘binning’.

## Abstract 2

Methods for efficient data extraction for neutron spin-echo spectroscopy with multipixel area detectors and – at a pulsed neutron source – multiple time-of-flight wavelength bins are presented.

## 1. Introduction   

**Figure 1:**
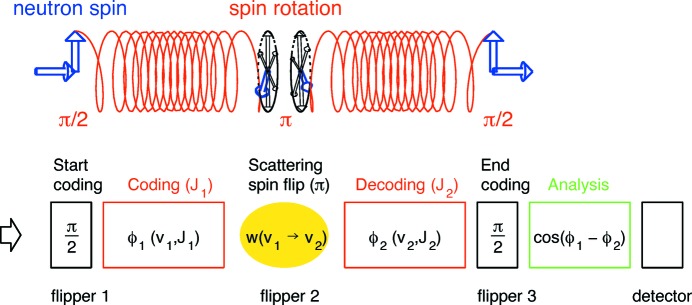
Scheme of an NSE (secondary) spectrometer: first arm (coding), sample position, second arm (decoding) and detector position.

High-resolution neutron spin-echo spectroscopy (NSE) was invented in the 1970s by Mezei (1972 ▸, 1980 ▸). The principle of the (various) NSE methods is to tag neutrons with a phase label (spin precession angle) in order to encode the individual velocity in the first arm of the spectrometer, and to reverse the phase manipulation in the second arm after the scattering from the sample (see Fig. 1 ▸). As NSE is a Fourier method, it yields the intermediate scattering function 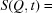 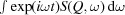 rather than *S*(*Q*, ω), where ℏω is the energy transfer and *Q* is the magnitude of the scattering wavevector. This technique has become the only method that extends the energy resolution of neutron spectrometers significantly below 1 µeV, even down to neV. For example, a 1 neV energy resolution corresponds to a Fourier time of about 0.7 µs, and it can be pushed even further up to 1 µs in favorable cases. On the other hand, the accessible NSE Fourier times can reach down to a few picoseconds, which allows for the coverage of up to six orders of magnitude.

A remarkable feature of the spin-echo technique is that it is very sensitive to tiny neutron velocity changes despite a rather broad velocity spectrum of the incoming neutron beam, thereby providing sufficient scattering intensity even at the highest resolution. Thus, for a large part of applications, the NSE provides a dynamic window to the small-angle neutron scattering (SANS) regime, where by analyzing the quasi-elastic scattering one can measure the mobility of the structures that give rise to the corresponding intensity [see *e.g.* Richter *et al.* (2005 ▸)].

There are a number of NSE spectrometers at neutron sources around the world (Farago, 1997 ▸; Schleger *et al.*, 1999 ▸; Rosov *et al.*, 2000 ▸; Holderer *et al.*, 2008 ▸; Nagao *et al.*, 2006 ▸; Longeville *et al.*, 2003 ▸; Häussler *et al.*, 2007 ▸), typically now implementing position-sensitive area detectors. Some NSE instruments are installed at pulsed spallation sources, which implies operation with time-of-flight (TOF)-tagged variable-wavelength neutrons (Ohl *et al.*, 2012 ▸; Hino *et al.*, 2013 ▸). Both allow the collection of information to be boosted but require more sophisticated techniques for data reduction to efficiently arrive at the physically relevant scattering function. The discussion of these techniques is the topic of the present paper. *DrSpine*, a Fortran 2008 program that requires only minimal external dependencies, implements the procedures described here.

### 1.1. Scattering intensities and scattering functions   

The scattering intensity that is to be analyzed in order to infer the physics of the sample corresponds to the double differential cross section (Marshall & Lovesey, 1971 ▸): 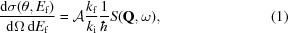
with 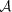 the scattering cross section or contrast factor. For a simple system of *N*_a_ atoms each with scattering length *b*_a_, 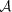 is given by 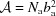; alternatively, if the typical SANS description in terms of scattering length densities applies (as in most NSE applications) it rather is given by 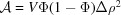, with sample volume *V* and Φ the volume fraction of the labeled (molecular) entities with scattering contrast Δρ with respect to the solvent or surrounding ‘matrix’. *k*_i_ and *k*_f_ are the magnitudes of the incoming and final wavevectors, **Q** = **k**_f_ − **k**_i_, ℏω = *E*_f_ − *E*_i_ is the difference between final and incoming neutron energies, and θ is the scattering angle.

For typical NSE applications the velocity (and hence the wavevector) changes are negligible compared with the initial value, and thus the factor *k*_f_/*k*_i_ ≃ 1 may be ignored and 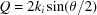. The factor 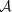 may have a more complicated form for complex molecular systems and is inferred from 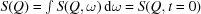.

At full symmetry, *i.e.* exact equality of the coding/decoding field integrals before and after the sample (π-flipper), the combination of the velocity coding/decoding leads to a full recovery of the initial phase if the scattering did not change the velocity (see Fig. 1 ▸). This is called the ‘echo’, in analogy to the Hahn spin echo first observed in nuclear magnetic resonance (Hahn, 1950 ▸). As soon as the velocity is modified in the course of the scattering process, a residual phase change is observed. Averaged over the distribution of many scattered neutrons, this results in a reduction of restored polarization [see *e.g.* Monkenbusch & Richter (2007 ▸)]: 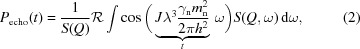
where 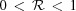 denotes the resolution function, 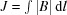 is the magnetic field integral along a neutron path through the spin precession region, γ_n_ is the gyromagnetic ratio of the neutron, *m*_n_ is the neutron mass, *h* is Planck’s constant and *t* is the Fourier time.

#### 1.1.1. The detector signal   

The distribution of the neutron-velocity changes after the scattering from the sample is proportional to the spectral part of *S*(*Q*, ω). The related difference of accumulated (precession) angles in the coding and decoding sections of the NSE instrument in combination with the analyzer transmission finally introduces an intensity modulation with the cosine of the angle difference, leading to the detector signal 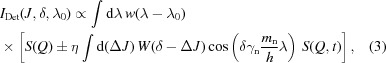
where
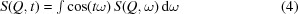
is the real part of the intermediate scattering function, *w*(λ − λ_0_) is the incoming neutron wavelength distribution centered at the nominal wavelength λ_0_, η is a factor that accounts for imperfect polarization efficiencies of the polarizer and analyzer and potential further depolarization due to imperfect flippers or sections with poor adiabaticity, and *W*(δ − Δ*J*) is the distribution of the field integral differences Δ*J* = *J*_2_ − *J*_1_ within the neutron trajectories. In other words *W* describes the field integral inhomogeneity, which is centered at the nominal field asymmetry δ. The latter is the primary parameter that is scanned during a phase scan. The width of this distribution determines the resolution and is in the range of a few µT m for a corrected (ultra)-high-resolution spectrometer. Even though the field integral deviations due to asymmetries and inhomogeneity and neutron wavelength distributions are typically not described by a Gaussian, it is instructive to approximate them as Gaussian distributions around their nominal values δ and λ_0_ with widths described by Σ and Λ, respectively: 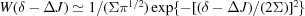 and 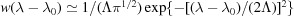. With these assumptions we arrive at analytical expressions that immediately reveal the salient features of the influence of these distributions on the NSE signal (for the full analysis we will go beyond that approximation). A detailed derivation of the resulting expressions is given in the supporting information. Here we quote the main results. The detector intensity *I*_Det_ depends on the (nominal) field integral *J*, the asymmetry parameter δ and the mean wavelength λ_0_: 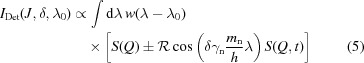
with the resolution function 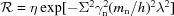. Note that the dominant contribution to the width Σ is proportional to *J*!. Further integrating over the wavelength distribution yields 
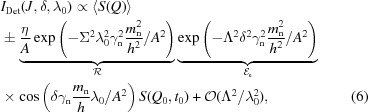
with *A* = [(2ΣΛγ_n_ *m*_n_/*h*)^2^ + 1]^1/2^, *Q*_0_ = *Q*(λ_0_) and *t*_0_ = *t*(λ_0_). For any practical purpose, *A* = 1 and thus is ignored in the *DrSpine* implementation. Under this condition [(2ΣΛγ_n_ *m*_n_/*h*)^2^ ≪ 1] the echo shape can also be taken as the cosine Fourier transform (CFT) of the wavelength distribution *w*(λ − λ_0_). In equation (7) the CFT for a narrow rectangular wavelength slice corresponding to a TOF bin will be used. 〈*S*(*Q*)〉 ≃ *S*[*Q*(θ, λ_0_)] denotes the average of *S*(*Q*) over the wavelength width. The higher-order terms 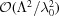 result from the λ dependence of *t* and *Q*. Their contribution is normally very small. For the possible finer λ slicing at a pulsed source it can be further reduced to virtually zero.

#### 1.1.2. The challenges   

Whereas the early spectrometers operated with only one detector (channel) and (for a given run) with a given wavelength λ_0_, the more recent instruments are equipped with multichannel area detectors and – if situated at a pulsed source – allow for time-of-flight tagging of the wavelength. Thus, primarily, we collect raw data for a large number of detector pixels (*p*_*x*_, *p*_*y*_) with the effective scattering angle θ = θ(*p*_*x*_, *p*_*y*_) and an azimuthal angle ψ = ψ(*p*_*x*_, *p*_*y*_), and in addition each pixel contains counts from a range of wavelengths binned in time-of-flight channels *k*. To measure the intermediate scattering function it is further necessary to scan the coding (field integral) asymmetry δ around the symmetry point and collect counts for a number of ‘phase points’ 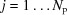 (δ = δ_*j*_) and to extract the prefactor of 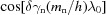, *i.e.* the echo amplitude, from it. This has to be performed for each *J*_*n*_ value from a list of *n* chosen values and for each value of the scattering arm θ_*m*_(0, 0) and eventually for an additional selection of wavelength frames. After resolution correction and background subtraction, the thus obtained individual pixel–time-of-flight–bin bits of information have to be collected in a consistent way in order to yield the best representation of the desired intermediate scattering function *S*(*Q*, *t*) or the usual normalized form *S*(*Q*, *t*)/*S*(*Q*).

**Figure 2:**
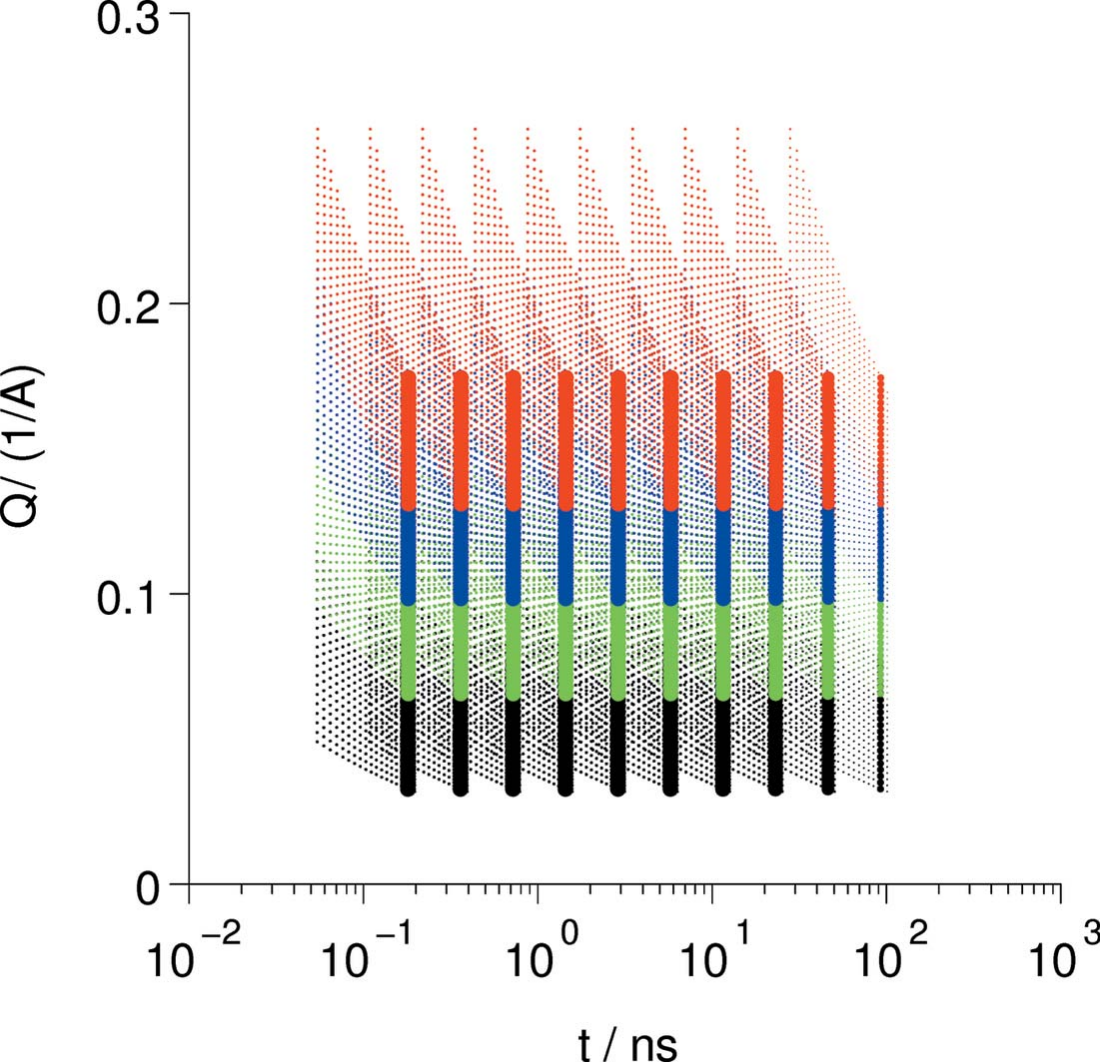
Coverage of (*Q*, *t*) space by a series of settings for *J*, the coding parameter (*i.e.* field-integral), and several nominal scattering angles (color coded). The dots indicate the location of individual detector pixels (zones). The fine dots illustrates the field at a pulsed-source instrument (SNS-NSE) while the thick lines apply to a typical selection of *J* and scattering angle settings (different colors) at a reactor instrument at a single given wavelength. In both cases the width of the color bands corresponds to the solid angle of the detector.

To achieve this, the appropriate contributions pertaining to a (*Q*, *t*) box must be collected and summed in a way that considers their individual information content. This ensures that a faithful representation of the *S*(*Q*, *t*)/*S*(*Q*) with the least possible error in the box range, as shown in Fig. 2 ▸, can be constructed.

**Figure 3:**
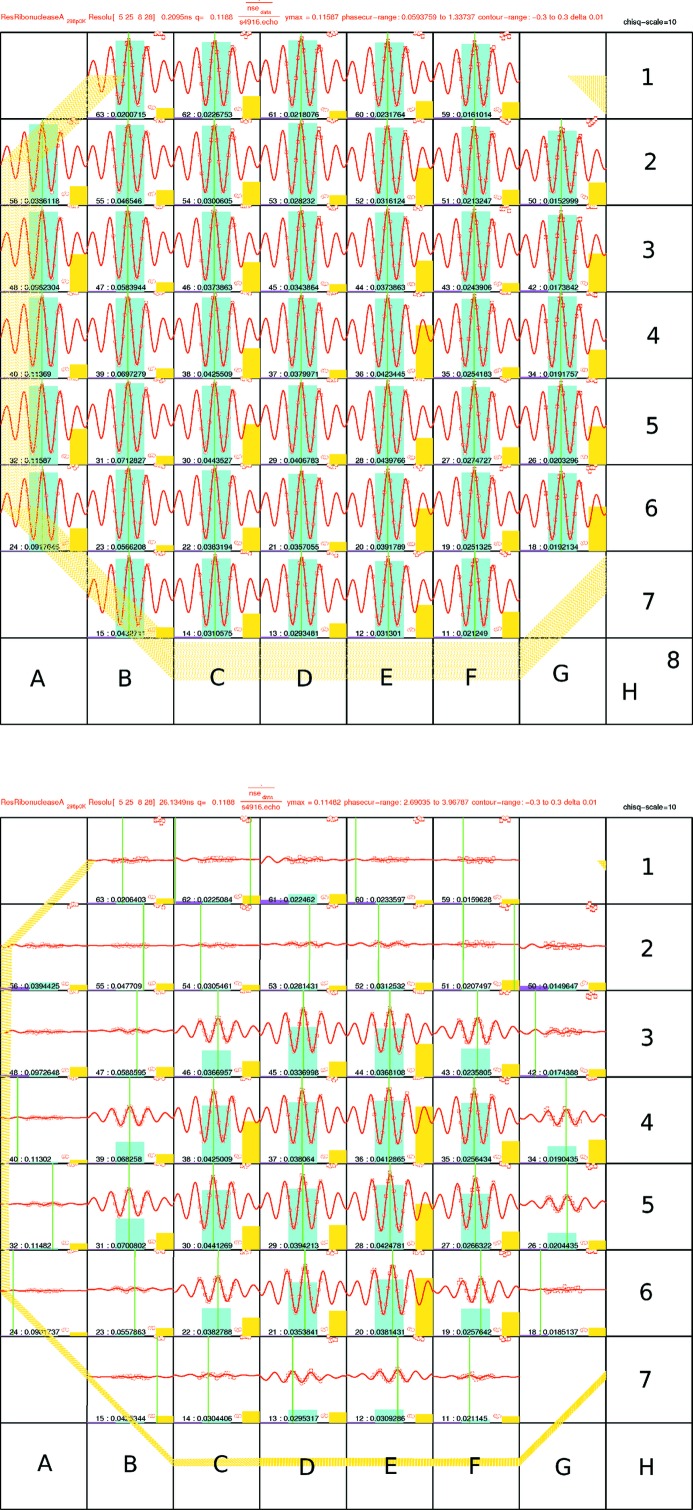
Detector portraits obtained at the SNS-NSE spectrometer for an 8 × 8 pixel binning. Each bin contains the sum of a 4 × 4 block of detector pixels. The binned wavelength covers the whole available range, which here is 8–11 Å. The image on the left side represents the situation for a small field integral of *J* = 0.0043 T m, whereas the image on the right side is for *J* = 0.5318 T m. The nominal scattering angle was about 6.9°.

A generic feature of the resolution properties is shown in Fig. 3 ▸. The echo signal from a resolution sample seen at different detector pixels, as well as the exact phase symmetry point, depends on both the value of the *J* parameter and the distance from the detector center. The loss of amplitude away from the center illustrates the limitation of the correction coils used to minimize Σ, in particular for the outskirts of the detector. At low field integrals nearly all detector pixels contain relevant information, whereas for increasing *J* the information content of the outer pixels diminishes. The goal of the presented evaluation scheme is to extract all of the available information.

Furthermore, we note that for different wavelength (bins) in the used time-of-flight-frame the single detector pixels correspond to different 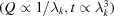 contributions to *S*(*Q*, *t*).

## 2. Methods   

### 2.1. Phase and resolution determination   

**Figure 4:**
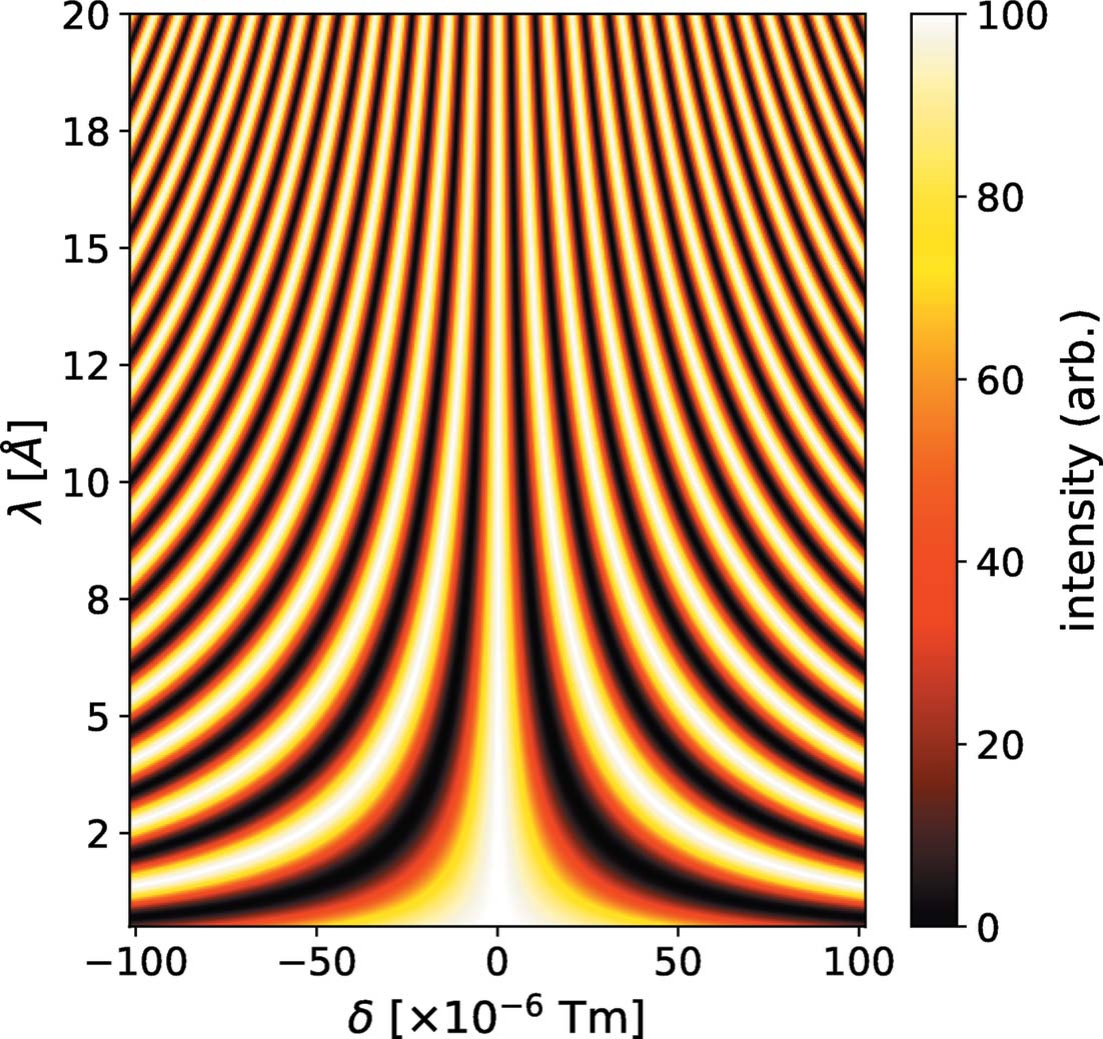
Computed echo oscillations (intensity coded) as function of wavelength (λ in °) and coding asymmetry δ in T m. At the true symmetry point δ = 0 the intensity modulation phase does not depend on λ.

A prerequisite to obtain the echo amplitude 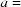 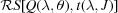 is the determination of the true symmetry point δ = 0 with respect to the asymmetry parameter of the instrument setting (*i.e.* the phase coil current). Furthermore, the resolution factor 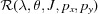 must be known in order to extract *S*(*Q*, *t*) from the echo amplitudes. For that purpose a reference experiment on a sample which effectively experiences only elastic scattering has to be performed. This serves to fix the true symmetry point, to determine the resolution and possibly also as a secondary cross section calibration standard. The dependence of the echo oscillations on wavelength and asymmetry are illustrated in Fig. 4 ▸.

From equation (5) it is obvious that, for a single defined wavelength [*i.e.* *w*(λ − λ_0_) = δ(λ − λ_0_)] and for a reference sample with *S*(*Q*, *t*) = *S*(*Q*) independent of *t*, the intensity variation after scanning the asymmetry (phase current) is proportional to a simple cosine function, 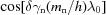. For such a very narrow wavelength band, the intensity modulation is simply along one horizontal line in Fig. 4 ▸. As an isolated observation this contains no clues about the actual maximum (minimum) of the true symmetry location. Considering several wavelengths, however, allows a unique identification of the symmetry location, which is characterized by the lack of dependence of the oscillation phase on λ. At continuous source experiments a 10–20% FWHM wavelength band is used. Integration thus reduces the modulation due to the obvious dephasing far from the symmetry (δ = 0). For a Gaussian wavelength distribution this leads to the echo-signal envelope factor [equation (6)] 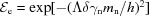 with 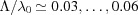. The latter is already a good approximation for the triangular wavelength distribution from a selector. Its exact form is given in the supporting material. At a pulsed source, the wavelength frame covered within one experimental setting is larger but with an intensity distribution far from Gaussian. Thus, for an exact representation of the envelope shape 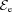 different approaches have to be used. The TOF-tagged λ distribution has a fixed absolute width limited by the frame overlap chopper system: Δλ = (*h*/*m*_n_)/(*Lf*), with *L* the source-to-detector distance and *f* the pulse repetition frequency. For the Spallation Neutron Source (SNS) NSE (Oak Ridge, TN, USA; Ohl *et al.*, 2012 ▸), depending on the chosen moderator detector distance (18, 21 or 24 m), the value is Δλ = 3.6–2.7 Å. These still comparatively small values are due to the high repetition frequency of 60 Hz. For smaller frequencies (*e.g.* at the new European Spallation Source, Lund, Sweden), Δλ ≃ 8 Å may be reached. Within the available Δλ frame one has the freedom to select arbitrary wavelength slices from the Δλ band in the frame. Thus various schemes to infer the correct symmetry location are imaginable, which utilize the fact that the location of the proper maximum must be independent of λ.

In practice, all determination schemes are limited by statistical counting errors, in particular since the determination has to be done pixel-wise. Thus the approach that proves most reliable here is to integrate over the whole frame and choose the best fit to the expected envelope. The shape of the echo signal *z*(ϕ) to be fitted reads as follows (Ohl *et al.*, 2012 ▸): 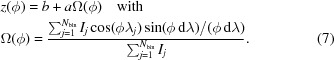

*N*_bin_ is the number of time-of-flight wavelength bins in any chosen wavelength (sub)-band, ϕλ is the phase angle with ϕ = (δ − δ_0_)γ_n_(*m*_n_/*h*) and the wavelength λ_*j*_ = λ_min_ + 2(*j* − 1)dλ. Thus Ω(ϕ) depends on the form of the normalized wavelength spectrum arriving at the detector, 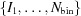. This is just an implementation of the λ integration over the 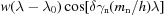 factor in equation (5). The intensity distribution within one TOF bin is assumed as constant and integrated, yielding the 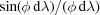 factor in equation (7). The time bin *j* nominally contains [λ_*j*_ − dλ, λ_*j*_ + dλ]. The wavelength-dependent intensity *I*_*j*_ is derived from the average of the detector intensity over the complete phase scan. This ensures that effects due to the implicit wavelength dependence of *S*[*Q*(λ)] are also accounted for. ϕ_*i*_ = *CI*_phase,*i*_ is proportional to the asymmetry (phase) current. Then, using the set of *N* ≥ 3 phase points ϕ_*i*_ and the corresponding counts *z*_*i*_ with statistical error δ*z*_*i*_, the echo amplitude *a* can be readily computed for all λ bins for any choice of time-of-flight λ binnings after insertion of the symmetry phase Δ = δ_0_γ_n_(*m*_n_/*h*), which has to be determined in a one-dimensional nonlinear optimization for Δ by minimizing the deviation 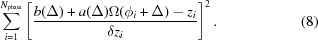
For the purpose of reliable determination of Δ it proved to be the best approach to use here data *z*_*i*_ that correspond to the sum over all valid TOF channels of the full wavelength frame with the associated echo-shape function Ω(ϕ). In that case the envelope of Ω(ϕ) is sufficiently peaked to discriminate the true symmetry point from solutions shifted by multiples of π in phase angle. By inserting the thus determined symmetry locations Δ, the amplitude for counts *z*_*i*_ obtained by any chosen wavelength binning then may readily be computed by 
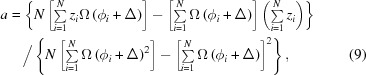
with the error 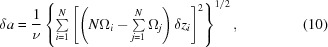
where ν stands for the denominator of equation (9). For the average *b*

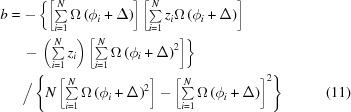
with error 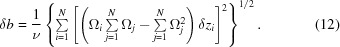



**Figure 5:**
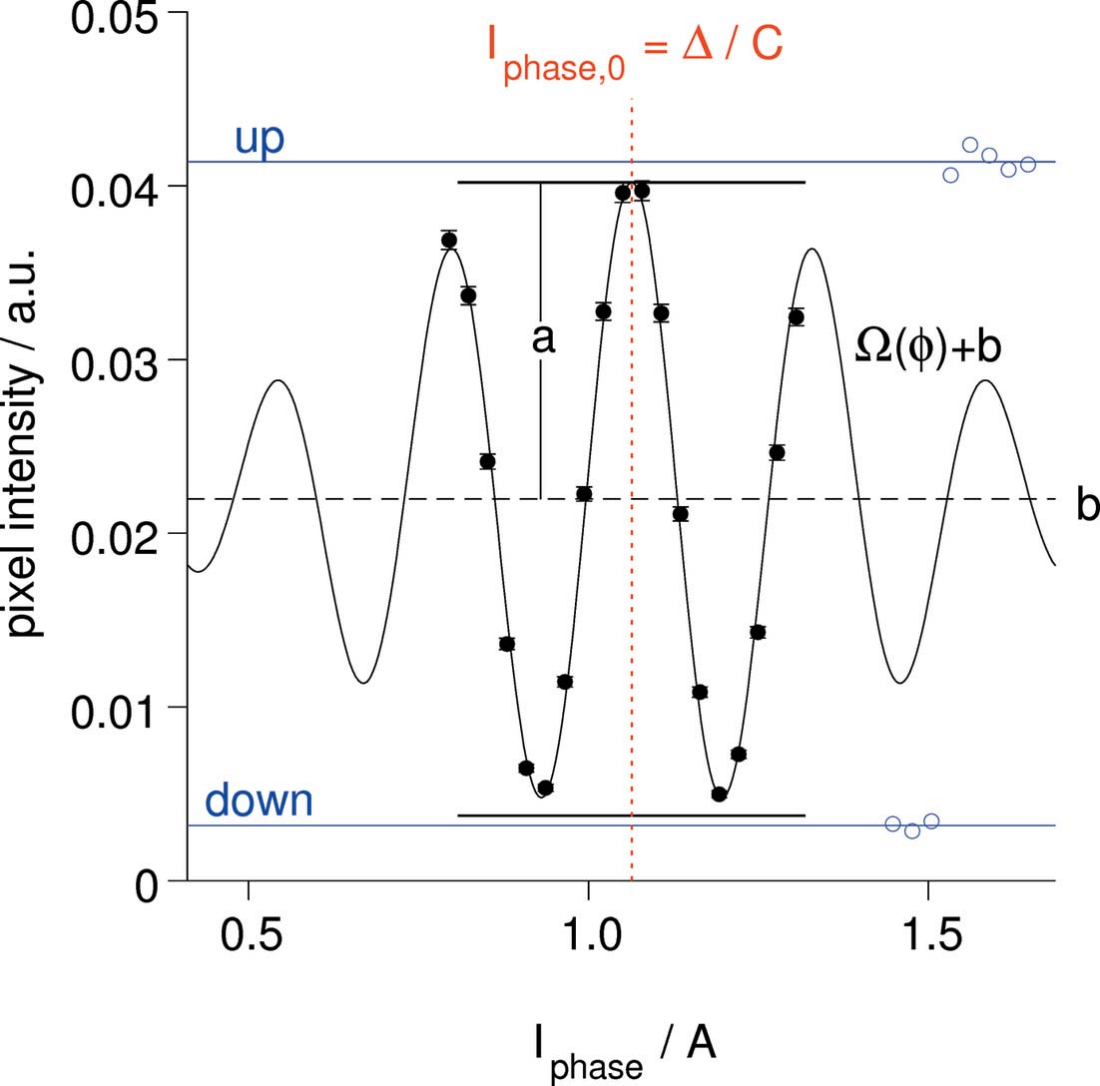
An echo signal in a 4 × 4 cm pixel cell in the central part of the SNS-NSE detector. The echo-shape function shown corresponds to the spectral intensity in the range λ = 5–8 Å obtained from the average time-of-flight spectrum measured at the detector. The phase symmetry current *I*_phase_ pertains to a symmetry coil of 80 turns around the beam. The echo results shown come from a reference sample measurement at *J* = 0.04 T m and 2θ = 8.6°. The solid symbols correspond to the phase scan around the symmetry point (red dotted line). The open symbols represent the up and down intensities. The extracted values for amplitude *a* and average *b* are indicated.

Fig. 5 ▸ illustrates how the various parameters are related to the data *z*_*i*_ from a phase scan and the echo shape Ω(ϕ). Note that in many cases the statistics in one pixel patch can be much worse and that the envelope modulation in λ sub-bands is less pronounced. We use the full bandwidth to determine the symmetry point because of the better definition due to the more pronounced envelope modulation and the better statistics due the inclusion of all time-of-flight channels.

**Figure 6:**
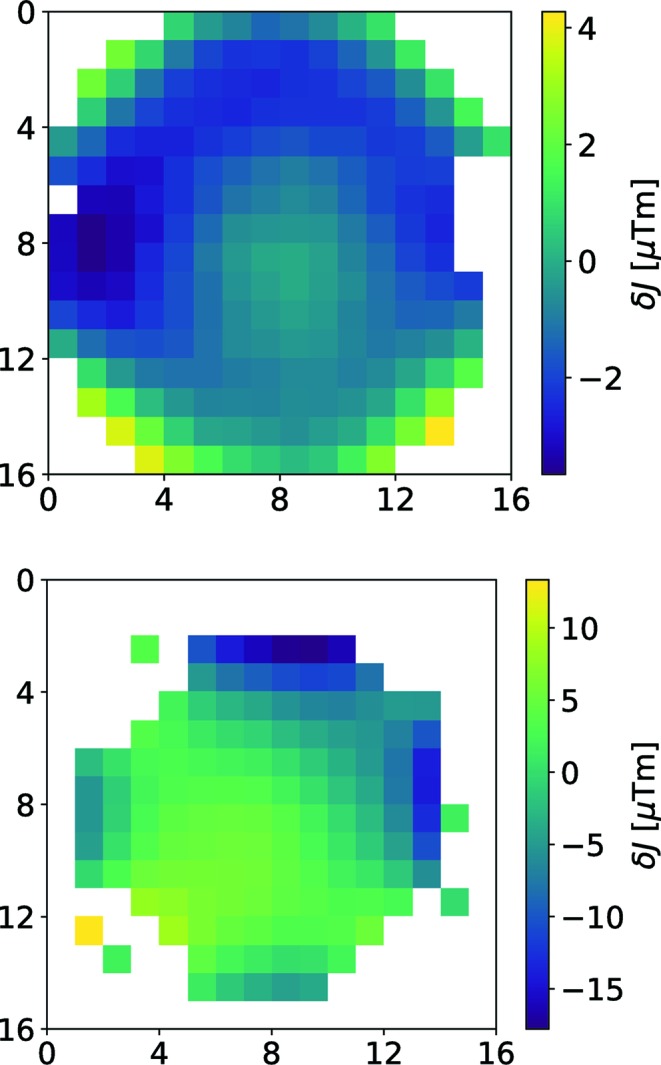
Phase maps for the SNS spectrometer (top) and the JNSE (bottom). White pixels contain no valid signal. The phase is given in terms of δ*J* in µT m. Pixel binning was 2 × 2.

The main source for the pixel-wise phase determination is the reference run. It should be performed such that the counting statistics are sufficient for phase determination and to yield resolution errors that are much smaller than the counting statistics due to the sample. The phase-map determination starts with a 4 × 4 pixel group in the center of the detector (controlled by the r.center_size program parameter) and then follows an outward spiral path toward the edges of the detector. Thus a smooth phase map is established, which is automatically included in the generated report (see supporting information). Subsequent echo amplitude extraction from sample and reference (and background) runs irrespective of the then possibly differently chosen TOF-channel binning rely on the thus determined phase map. This requires only direct computation using equations (9)–(12) [Disp-formula fd11] [Disp-formula fd12]. As an option, the phase of the sample run can be checked and corrected by a global offset by minimizing the residual error (weighted sum) of all pixels as a function of an offset phase applied to the phase map as determined using the reference data. The values are always given in the automatic report. Examples are shown in Fig. 6 ▸.

For each setting of *J* and θ, detector pixels that exhibit too low a resolution or too few counts or cannot be fitted with equation (7) are flagged as ‘non-valid’ and ignored in further steps. Note further for a reactor (steady-state) instrument the envelope width is fixed to the shape determined by the wavelength width as imposed on the incoming beam by a mechanical velocity selector (typically 10–20% FWHM). A velocity selector provides typically a triangular shaped envelope instead of a Gaussian. Both functions can be selected in the data evaluation program.

### 2.2. Echo determination   

In a first step, the pixelated reference (resolution) phase scan data have to be treated as described above to extract the exact symmetry offsets and echo amplitudes that lead to the resolution factors. Once the resolution factors and symmetry phases have been determined, the pixel- and time-of-flight-bin-wise determination of echo amplitudes *a*(*p*_*x*_, *p*_*y*_, *k*, θ_0_, frame) according to equation (9) can be performed. For the SNS-NSE spectrometer, for example, the symmetry phases as determined from the reference experiment are stable and thus reliable because it has a magnetic shielding excluding external magnetic disturbance. For other instruments, such as the Jülich NSE (J-NSE), the change between reference and sample experiments is an issue and it may be necessary to readjust symmetry phases using the echo signals of the sample – as far as statistics allow. While the primary results of the symmetry scan are the echo amplitudes 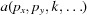, the desired output in terms of *S*(*Q*, *t*) or *F*(*Q*, *t*) = *S*(*Q*, *t*)/*S*(*Q*)^1^ requires further treatment. For that purpose the maximum polarization obtainable is determined from counting results corresponding to direct scattering on the detector without any coding/decoding or polarization manipulation (‘spin up’), but with the polarization analyzer in the beam, and then in the nominal blocking situation with only the π-flip (spin reversal) active (‘spin down’) (see *e.g.* Fig. 5 ▸). The obtained difference yields *I*_up_ − *I*_down_ ∝ η*S*(*Q*) and η = (*I*_up_ − *I*_down_)/(*I*_up_ + *I*_down_). Thus for each pixel bin from the sample data we have 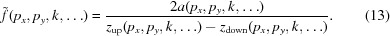
Applying this procedure to the reference sample, the corresponding resolution was obtained: 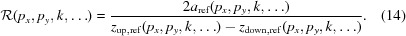
Then the resolution-corrected pixel-bin contributions 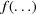 and 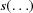 to *F*(*Q*, *t*) and *S*(*Q*, *t*) are 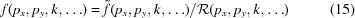
and 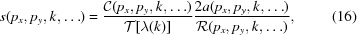
where 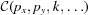 denotes an optional (in future implementations) calibration factor (see Appendix *B* [App appb]) and 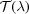 the sample transmission. Typically, the pixelized *f* and *s* values have sizeable statistical errors δ*f* and δ*s*. Finally, the scattering from a background sample may be subtracted pixel-bin-wise: 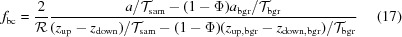
and 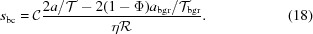
The background correction methods are discussed further in Appendix *A* [App appa].

### 2.3. Data collection, harvesting results   

After evaluation of all available NSE echo files pertaining to the sample under consideration, one can start to collect all results that contain information on *S*(*Q*, *t*) or *F*(*Q*, *t*) pertaining to specific (*Q*, *t*) pairs. For this purpose, a boxed grid for *S*(*Q*, *t*) may be defined, covering a number of *Q* and *t* slices 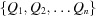 and 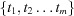 (see Fig. 8 among the *Examples* [Sec sec4] below).

To arrive at a representation of *S*(*Q*, *t*) with all information available, the *Q*, *t* values *Q*(*p*_*x*_, *p*_*y*_, *k*, θ_0_, frame) and *t*(*p*_*x*_, *p*_*y*_, *k*, *J*, frame) of all available valid pixel bins are assigned to the corresponding grid box (*i*_*Q*_, *j*_*t*_) and the corresponding amplitude or normalized amplitude information *s* ± δ*s* or *f* ± δ*f* is added to the grid box. To do this in a way that preserves the significance of the individual contributions and does not explicitly depend on the number of contributions, we use the following scheme, which is a key method to preserve the data information content:

(1) If the grid cell is still empty, copy the value of any kind of elementary data *s*_*i*_ ± δ*s*_*i*_ derived from the contents of a contributing pixel–time-of-flight bin, *e.g.* typically *S*(*Q*, *t*) and its error, and put actual *Q* and *t* values in corresponding fields of the grid.

(2) If the grid cell already contains data *s*_0_, δ*s*_0_ (where *s*_*i*_ here stands for any result in the bins to be combined, *e.g.* scattering function *s* or normalized scattering function *f* or other), insert the additional information *s*_1_, δ*s*_1_ in the following way, where the relative weight of the added information is 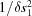: 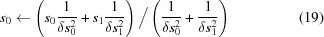
and for the errors 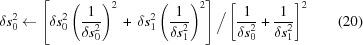
in parallel. Accordingly, the effective *Q* and *t* values of the grid cells are updated by adding the new values with the same weight factors as the *s* values. This weighting scheme ensures that compartmentalized counts *z*_*i*_ that are recombined to 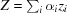 yield the appropriate combination factors α_*i*_ (*i.e.* the relative size of the compartment, *e.g.* time bin or pixel area) and the correct error, identical to what a plain summation of the original counts would yield for the resulting value and the counting-statistics-related error (see supporting information for a concise derivation). Using the same weights, the (*Q*_*i*_, *t*_*i*_) values of the bins are combined to yield effective centers (*Q*_c_, *t*_c_) associated with the corresponding histogram box. Finally a table containing the data from all nonzero grid cells can be generated and is provided as output. Examples for the resulting mapping are displayed in Fig. 8. It is obvious that the full power and validity of this method is only realized if all errors δ*s* are correctly computed and propagated from the initial count statistics.^2^

## 3. Implementation   

Basically *DrSpine* only requires a list of raw data run numbers for all relevant reference runs (flagged with role ‘reference’), all sample runs (role ‘sample’) and, if present, background runs (role ‘background’). The data formats that can presently be read are those from SNS-NSE and J-NSE. Typically some pre-binning of detector pixels (from 1024 to 64) and (1–42) time-of-flight bins is performed during reading. The further matching of reference sample, background and evaluation to *S*(*Q*, *t*) is automatic. Within the (*Q*, *t*) regime covered by any available combination of wavelength, arm setting, pixel-dependent scattering angle and coding parameter *J*_*i*_, a wide range of *Q* and *t* binning schemes can be chosen for data collection. Since the collection step typically requires not more than a few seconds, it is foreseen that in a normal procedure a selection of several (say five) standard different ‘default’ histogramming options will be produced, reported and supplied as tables. Conversion of the report to a well formatted document requires the installation of TeX (Rahtz *et al.*, 2018 ▸).

### 3.1. Paradigm for user support and information   

**Figure 7:**
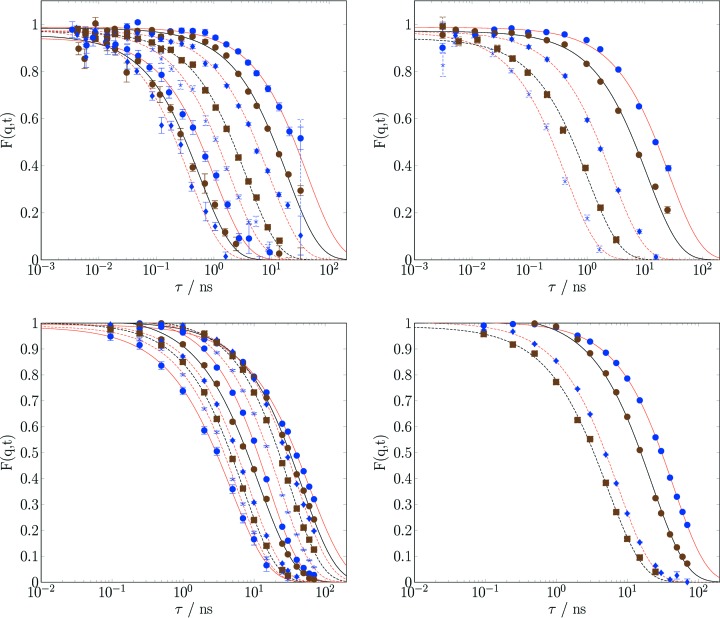
Examples of evaluated data. Upper part: Polymer dendrimer solution (Lederer & Kruteva, 2016 ▸) data collected at the SNS-NSE instrument using a 5–8 Å neutron wavelength frame, four settings of the nominal scattering angle (3.71, 7.31, 12.4 and 19.8°) and a total of 15 settings of the field integral *J* between 10^−4^ and 0.5 T m (11 normal and four short-time mode). Lower part: SDS micellar solution data collected at the J-NSE Phoenix spectrometer using 8 Å (20% FWHM) neutrons and four settings of the nominal scattering angle (see Fig. 10 ▸). Shown are normal (left) and coarse-grained (right) *F*(*Q*, *t*) histogramming of the same data, obtained as part of a default data reduction scheme presented to the user at the end of the evaluation run. Lines are stretched exponential fits drawn to guide the eye.

Since the whole process including the generation of the report is done automatically in a few minutes even on a current personal computer with no further operator interference needed, a close monitoring of the progress of the experiment is enabled (provided the references are available). The user (novice or expert) will automatically be provided with a comprehensive report containing all references and auxiliary information of the processed files. The most important information is that on the experimental setup, the binning used and the selection of curves for *F*(*Q*, *t*) from the default histogramming schemes, which are generally fitted with general standard model curves (*e.g.* stretched exponential plus background), plus effective diffusion estimates as illustrated by Figs. 7 and 9, discussed in the next section[Sec sec4]. Only the display of the report requires TeX to be installed. Further and more sophisticated model comparisons or evaluation then have to rely on separate independent consideration of the tabulated *S*(*Q*, *t*) data in the simultaneously created (human readable) output files for all tried histogrammings.

## 4. Examples   

Fig. 7 ▸ shows the results obtained for typical soft-matter samples. The curves shown are obtained for different final histogramming box settings, thus enabling us to focus on different aspects of the scattering function, for example trading statistical error versus number of different *Q* and/or *t* values, depending on the primary question to be answered. Normally this means simply choosing the results from one of the different automatically applied histogramming schemes. If needed, further custom histogramming schemes can easily be added to the evaluation.

**Figure 8:**
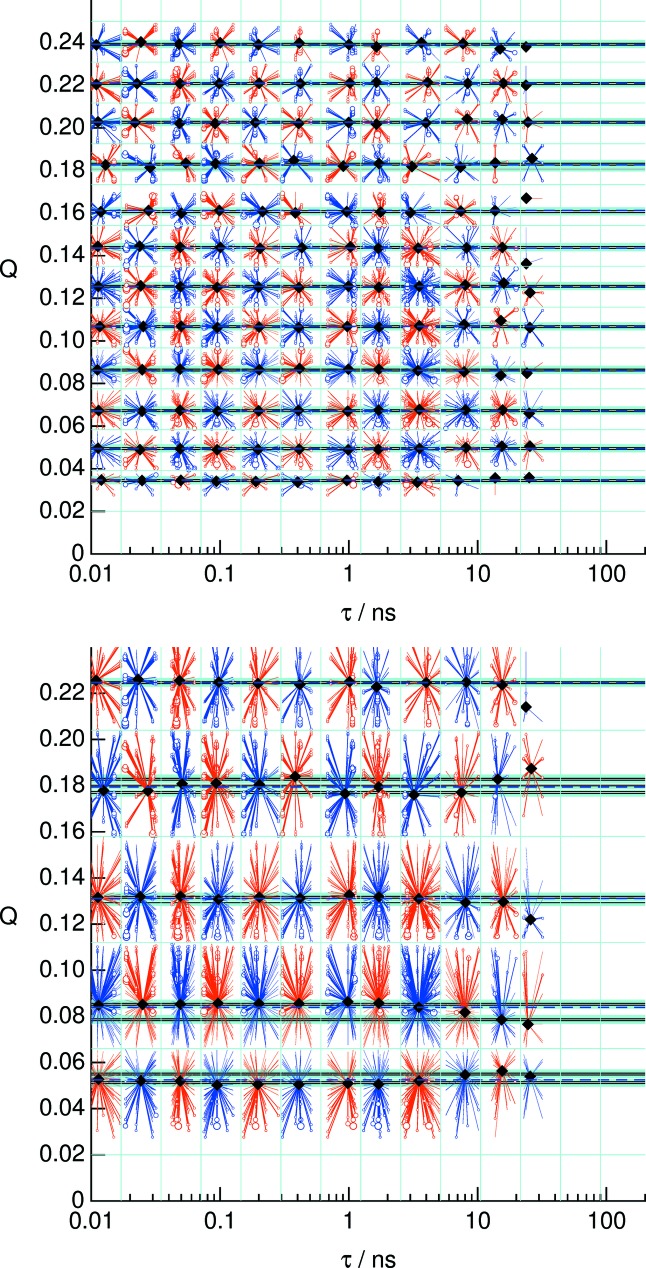
The ray plot representing evaluated (*Q*, *t*) points alongside the corresponding contributions. The data are the same as in the upper part of Fig. 7 ▸. The black diamonds show the effective values assigned to each (*Q*, *t*) point on a grid (thin blue horizontal and vertical lines). The rays from each diamond symbol point to the location of a pair of (*Q*, *t*) values that contribute to that point, and the circle size indicates the weight of the contribution. Since the best estimates of the nominal *Q* values indicated by the diamonds depend on the weights and distribution of the contributing pixels, these values fluctuate around the average value. In order to be able to represent data in the common *t* table for given *Q* values without introducing significant deformation of the curve, only points with values within a narrow ‘*Q*-grouping catch size’ are accepted to enter the corresponding table. The checkerboard-type red/blue coloring is an aid to guide the eye only. The black dashed horizontal lines surrounded by a light blue zone (the *Q*-grouping catch size) indicate the *Q* values used to create the plots in Fig. 7 ▸. This type of plot allows for an easy assessment of the *Q* variation within each *Q* group and is a part of the standard report.

The grouping into curves *F*(*Q*, *t*) with fixed *Q* and varying *t* is in accordance with the box histogramming. However, as Fig. 8 ▸ illustrates, the best value of *Q* may vary from the box centers. While for most applications the slight variations of *Q* with respect to the average is negligible, the most precise fitting to a model may be performed on the set {(*Q*_*i*_, *t*_*j*_), *F*(*Q*_*i*_, *t*_*j*_)} with (*Q*_*i*_, *t*_*j*_) the star centers in Fig. 8 ▸, *i.e.* the importance-weighted (*Q*, *t*) points. This weighting is a genuine measure to ensure consistency and accuracy of the result irrespective of the details of the chosen binning.

**Figure 9:**
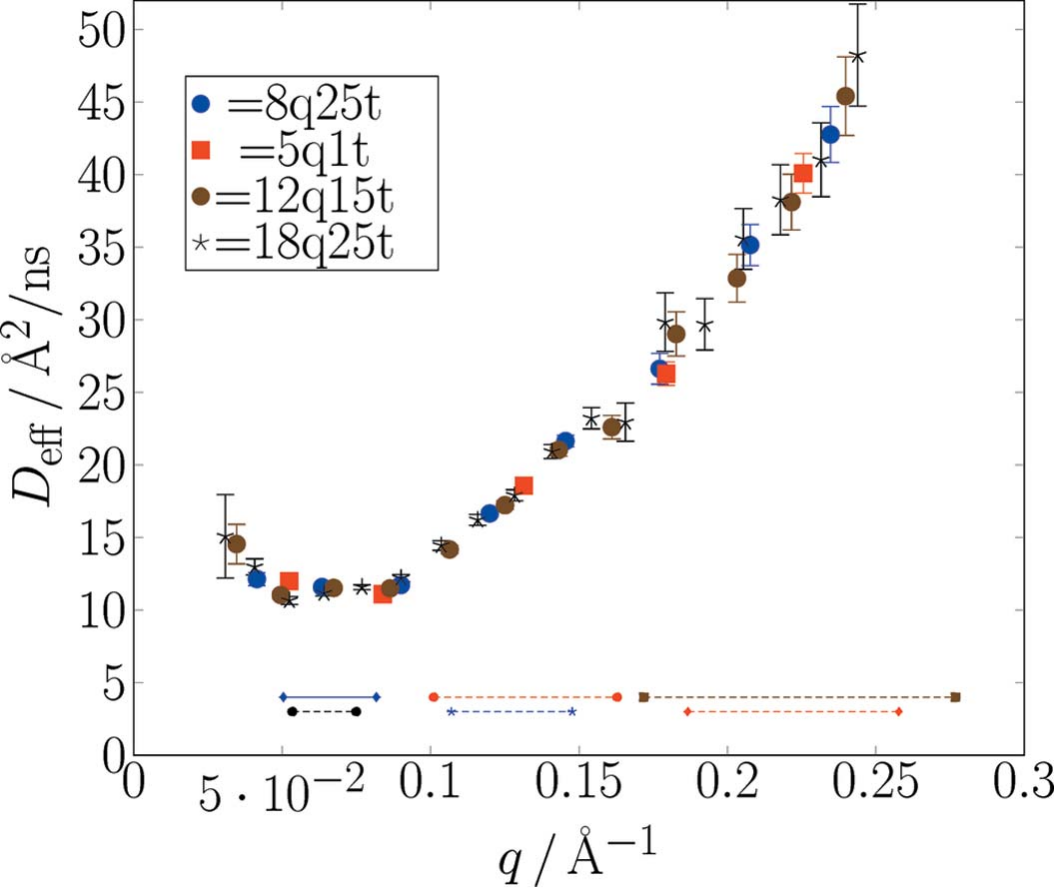
A compilation of (automatically) derived effective diffusion coefficients obtained by fitting the *F*(*Q*, *t*) data from the upper part of Fig. 7 ▸ (SNS-NSE data). Results shown here come from different histogramming schemes: [number of *Q* bins (linear), number of *t* bins (logarithmic)] = [8, 25], [5, 15], [12, 15], [18, 25]. All results are consistent, but observe the different error bars and range coverage. Horizontal lines indicate the nominal (detector center) *Q* values for each of three scattering angle settings. The upper lines correspond to a wavelength frame λ = [5 Å, 8 Å] and the lower lines to a frame λ = [8 Å, 11 Å]. The experimental range due to detector width amounts to about ±0.02 Å^−1^ beyond the range indicated by the horizontal lines.

For each of these schemes, the report contains tentative automatic fits to generic relaxation models and estimates for effective diffusion *D*_eff_(*Q*) as an immediate guide for the user. This feature, however, depends on the applicability of the model to the actual problem. At the moment plain stretched 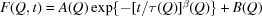 exponentials are used with free or fixed parameters *A*, β and *B*. The lines in Fig. 7 ▸ are created by this automatic mechanism. The corresponding *D*_eff_(*Q*) values are shown in Figs. 9 ▸ and 10 ▸. The data stem from typical soft-matter sample experiments at the SNS-NSE with time-of-flight-tagged wavelength bands (dendrimer solution) and at a continuous single-wavelength band-reactor instrument (J-NSE Phoenix, MLZ Garching; Pasini *et al.*, 2019 ▸) [sodium dodecyl sulfate (SDS) micelles in D_2_O]. Figs. 9 ▸ and 10 ▸ display all results of the different (*Q*, *t*) binning schemes to show that all schemes yield consistent results.

The corresponding data are supplied in the output *S*(*Q*, *t*) tables.

## 5. Conclusion   

With the algorithms and procedures as implemented in *DrSpine*, a unified approach to the extraction from multi-detector and multi-wavelength raw data from neutron spin-echo spectrometers at continuous and, in particular, at pulsed sources is described. The method of inverse-error-weighted (incremental) combination of results may also be applied to other raw data extractions that rely on the combination of experimental information from many bins with varying significance (sensitivity, illumination, resolution *etc.*). Using automatic matching and consistent binning, we have presented a streamlined procedure for a typical set of 10–20 raw data scans on resolution, background and sample covering several settings of nominal scattering angle and possibly subsets of the coding parameter *J* (*e.g.* from short-time and normal mode). Dynamic (error-weight-controlled) masking allows the use of each bit of information contained in the raw data sets.

## 

## Supplementary Material

Example for an automatically generated report pertaining to the results shown in the lower part of Fig. 7. DOI: 10.1107/S1600576719010847/po5149sup1.pdf

Derivation of the echo expressions in Gaussian approximation, derivation of the data combination rule and comments on example report. DOI: 10.1107/S1600576719010847/po5149sup2.pdf

## Figures and Tables

**Figure 10:**
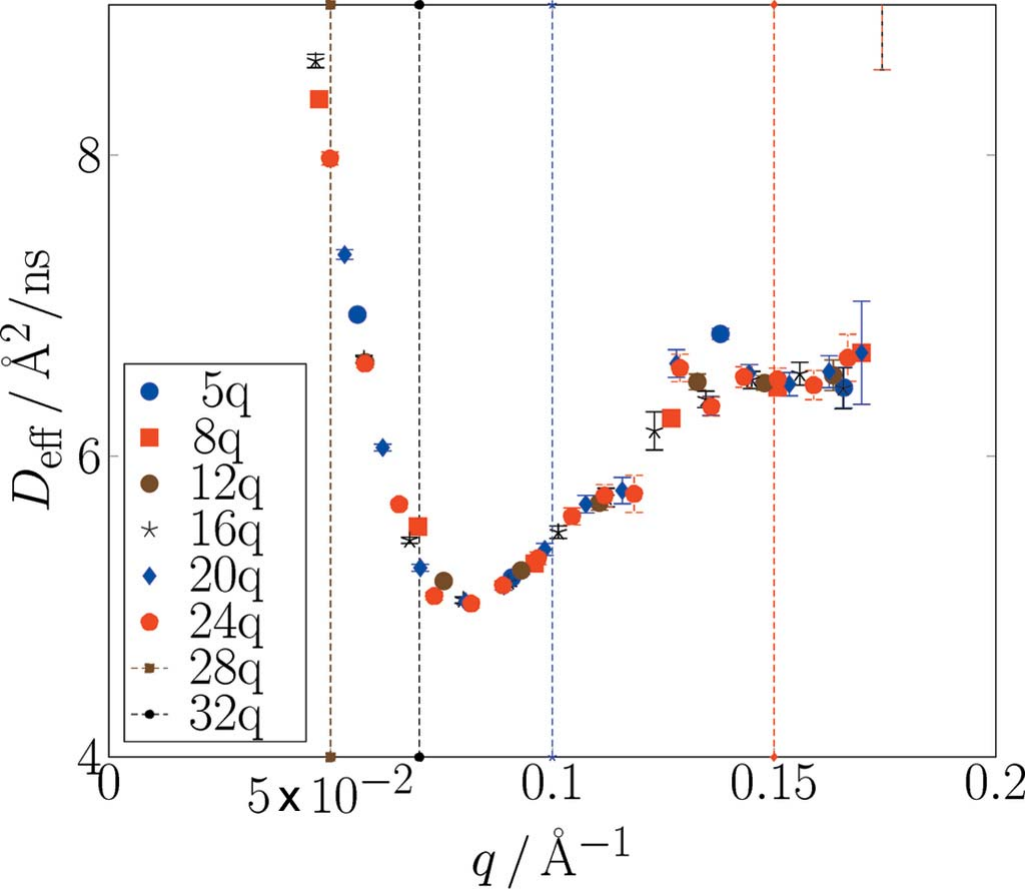
A compilation of (automatically) derived effective diffusion coefficients obtained by fitting the *F*(*Q*, *t*) data from the lower part of Fig. 7 ▸ (J-NSE data). Results shown here come from different histogramming schemes. The vertical dashed lines indicate the *Q* values of the nominal scattering arm settings. It is obvious that because of the use of only one fixed wavelength the detector coverage is insufficient in the larger scattering angle gap between *Q* = 0.1 Å^−1^ and *Q* = 0.15 Å^−1^. All other results are consistent, but observe the different error bars and range coverage.

**Figure 11:**
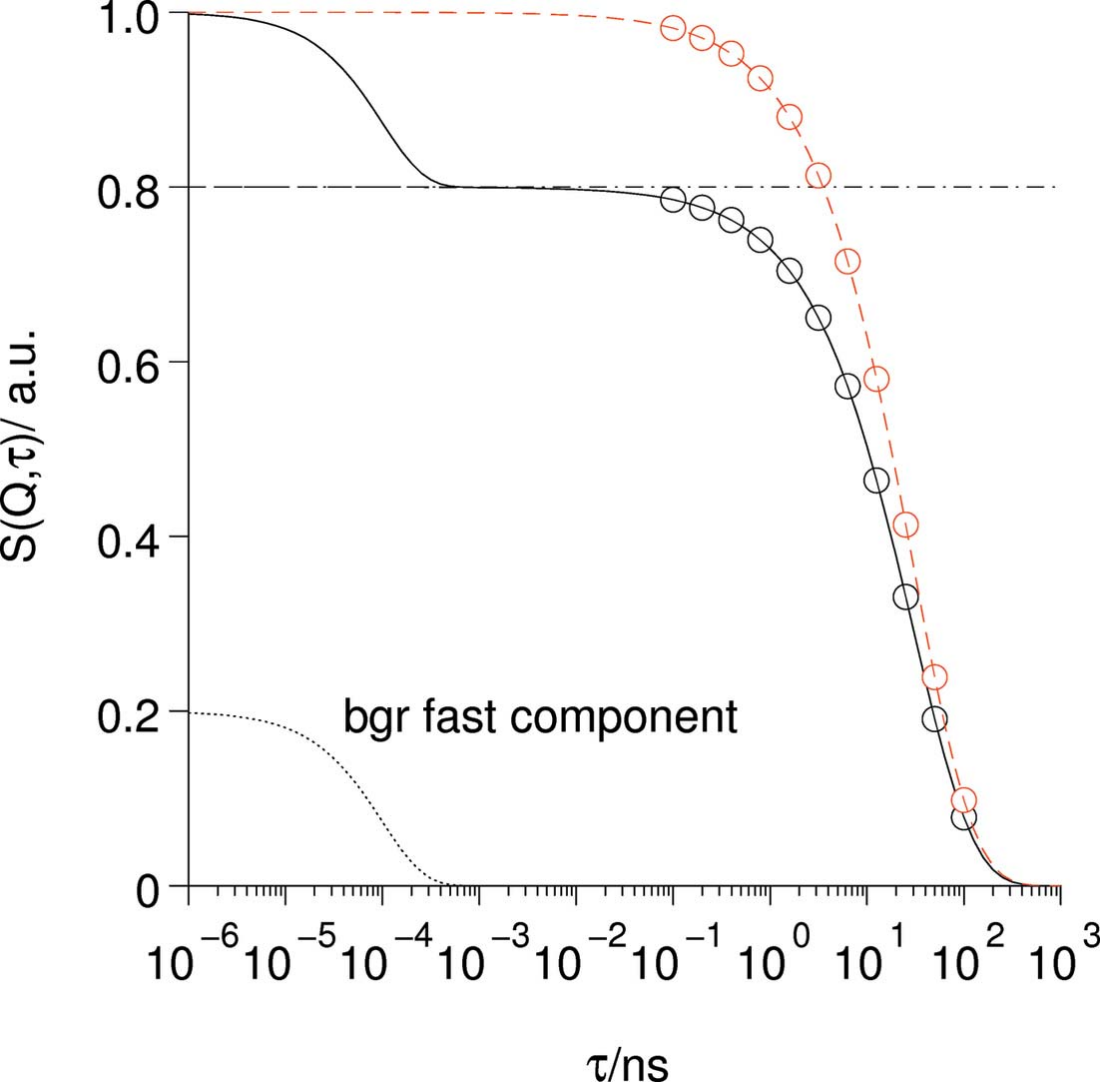
Role and effects of the fast background contribution. The commonly observed fact that normalized NSE relaxation curves [*S*(*Q*, τ)/*S*(*Q*)] do not extrapolate to 1 if τ → 0 is caused by the presence of a very fast contribution to the scattering that only contributes at times shorter than the short-time limit of the NSE instrument. The red curves indicate the data after correction with the factor resulting from the amplitudes of slow (NSE) and fast dynamic contributions.

## Footnotes

1 In the following we use the notation *S*(*Q*, *t*) for *S*(*Q*, *t*) and *F*(*Q*, *t*), whatever applies in the context. Currently NSE evaluations are usually made to yield *F*(*Q*, *t*) = *S*(*Q*, *t*)/*S*(*Q*, 0). The *DrSpine* software has also provisions to yield *S*(*Q*, *t*).

2 Caveat: When combining normalized results from (binned) detector pixels to sum a quantity from pixel-wise-derived contributions over a larger detector, plain error propagation may overestimate the error. This pertains, for example, to the contribution due to statistics of the normalizing monitor values. Since monitor values are common to all detector pixels, their influence on the statistical fluctuation of normalized pixel data is correlated.

## Appendix A Background subtraction

The choice of what exactly is to be considered as directly sample-related background contributions pertains to the nature of the sample and the scattering contribution that contains the desired information. Some prototypes of samples for which a corresponding distinction between signal and background can be made are, for example, macromolecular or colloidal solutions or polymer melts, where the polymer is protonated and has volume fractions ϕ between ∼2 and 20%. The majority component, *i.e.* the solvent or ‘matrix’ polymer of the melt, is deuterated. In those cases the primary scattering signal to be analyzed is the coherent scattering function of the labeled polymer chains – as in the simple case is predicted by the Rouse dynamics (Doi & Edwards, 1994 ▸). For labeled macromolecules at low *Q* values the scattering intensity in the echo signal is dominated by the labeled (macro)molecules and conveys their dynamics. With increasing *Q*, the spin-incoherent proton scattering of the labeled compound, spin incoherent scattering from a deuterated majority compound and multiple scattering involving large intermediate *Q* will start to significantly contribute to the echo signal. The latter certainly is to be considered as (fast) background. Incoherent scattering contributions are in principle part of the genuine scattering signal and carry dynamic information. Still, those from the solvent/matrix are background. Again their relative importance rises as *Q* increases, such that their typical contribution grows from a few percent at *Q* = 0.05 Å^−1^ to several tens of percent at *Q* = 0.2–0.3 Å^−1^. They often have fast dynamics, most probably stemming from multiply scattered large *Q* contributions. As the source of this type of background scattering is considered to be the matrix, its contribution to the sample scattering scales with the volume fraction of matrix polymer in the sample. The composition of the scattering signal containing the background described above is illustrated in Fig. 11 ▸. The fast dynamics relax the echo signal at times much lower than the smallest available NSE Fourier time (symbols indicate measured NSE points). It is obvious that extending the NSE range down by one or two orders of magnitude (short-time mode) may serve to determine the amount of fast background without additional measurement of a background sample, thus also eliminating any concerns about the actual background source.

The correction of only this type of background basically is the determination of the extrapolation of the echo intensity at τ → 0 by subtraction of the fast background component from the normalizing up–down intensity only. In the general expression for background correction, equation (17), the corresponding factor results from the modification in the denominator.

Depending on the nature of the background sample there may be scattering contributions that show a dynamic within the NSE-accessible range and/or slower components, *i.e.* a nonzero *a*_bgr_ term in the numerator of equation (17). In these cases there is a background contribution also to the echo signal.

A dynamic component with low intensity can always be attributed to the incoherent scattering from the deuterons (and possibly residual protons) in the matrix (*e.g.* melt matrix or solvent). The echo-signal contribution is scaled by −1/3 and displays the dynamics of polymer segment or solvent molecule diffusion. These contributions are mostly only relevant at larger *Q* (> 0.2 Å^−1^). The next possible source of background scattering is the sample container and possibly its immediate surroundings. Except for the subtle difference that the sample container is always present in full, it may be considered like the sample scattering. The subtle difference means that the container scattering should not be multiplied with the (1 − ϕ) factor. Note that, despite the fact that scattering from the sample environment, windows *etc*. is usually elastic, the corresponding background signal may nevertheless exhibit a kind of pseudo-dynamics, since the location of the scattering differs significantly from the nominal sample position and thus additional echo-signal loss will occur due to dephasing.

### A1. Optimized counting time distribution

In order to determine the best distribution of a given counting time between sample and background experiments we use the following consideration: Assume that in any region of interest on the detector (and in some wavelength band) we observe counting rates *r*_s,bgr_ for the sample or background, respectively. There we will accumulate *N*_s_ = *r*_s_ *t*_s_ and *N*_bgr_ = *r*_bgr_ *t*_bgr_ counts during the sample experiment with counting time *t*_s_ and the background experiment with counting time *t*_bgr_. Consequently, the statistical errors of these rates are

The final result of the corrected scattering rate after transmission and background subtraction follows from

and the corresponding error

After inserting δs and considering the squared error for simplicity, we find

where we set *t*_c_ as the total available counting time, distributed as *t*_c_ = *t*_s_ + *t*_bgr_ between sample and background experiment. For a coarse estimate at the time of planning the experiment, minimizing equation (24) with respect to *t*_bgr_ and assuming  yields a good estimate of the optimum time fraction for background measurement:

For example, for a background amounting to 10% of the sample scattering (assuming nearly equal transmission), equation (25) yields *t*_bgr_ = *t*_c_[(0.1)^1/2^ − 0.1]/(1 − 0.1) ≃ 0.25, *i.e.* in this case the available time for the sample and background experiments should be distributed as 3 (for sample):1 (for background) to obtain the best possible statistics within a given time slot *t*_c_ for sample and background scans.

## Appendix B Absolute calibration

As an extension, we are currently developing the procedures in which the reference samples may serve as secondary calibration standards such that the *DrSpine* program will be able to deliver in addition to the normalized *F*(*Q*, *t*) = *S*(*Q*, *t*)/*S*(*Q*) values also the *S*(*Q*, *t*) tables. The latter would have the advantage that combinations and background subtractions can be made across different experiments and even instruments. A set of well defined and preserved reference samples (needed anyway for resolution correction) may be used to serve as standards for absolute calibration. This requires a one-time measurement of dΣ(*Q*)/dΩ of these samples in absolute units on a SANS machine or diffractometer. Let us define sensitivity factors ζ_(*i*, *j*), *k*(λ)_ and flipping ratios (← polarization efficiencies) for each pixel and λ by using the corresponding monitor-normalized up and down intensities of the reference measurement  and  in order to get the pixel-bin calibration factors:

with *d*_eff_ the sample thickness and  the transmission [depending on derivation of the *S*(*q*)_Ref_ data].

### b1. Amplitude-derived *S* ^NSE^(*Q*, *t*)

In order to cope with general cases with some decoupling from the up–down normalization (*i.e.* coherent–incoherent combinations where *I*_up_ ≃ *I*_down_) *etc*., *DrSpine* may yield an absolute calibrated

for soft-matter-type samples or

for paramagnetic samples. The collection of the pixel-bin contributions follows the same error-weighing scheme as used for the normalized NSE data. However, here we collect the calibrated and resolution-corrected amplitude values directly, which yields additional information on *S*_coh_(*Q*, *t* = 0) and *S*_inc_(*Q*, *t* = 0) as extracted from the up and down intensities. These values are obtained with the collected *S* ^NSE^(*Q*, *t*) spectra (for soft-matter-type problems). For paramagnetic-type samples, *S*_paramag_(*Q*, *t* = 0) and *S*_nuclear_(*Q*, *t* = 0) are supplied by a slightly different route.

*S* ^NSE^(*Q*, *t*) then is obtained via collection of the pixel-bin contributions:

where *a*(*i*, *j*, *k*) is the pixel-bin-associated echo amplitude and  the resolution.

## Appendix C Program availability

Typical use of this front-end evaluation program is for extraction of *S*(*Q*, *t*) at the end of the experiment. For this purpose, web-based access is the standard mode of use at MLZ in Garching and at SNS in Oak Ridge. Thereby, a bundle containing a comprehensive set of tabulated *S*(*Q*, *t*) data together with a report containing all technical details of the experiment and evaluation is created, which allows for instrument-independent physical modeling and analysis. The associated report is a backup source with detailed technical information on the experiment and the role of potential disturbance by, for example, magnetic fluctuations in the environment *etc*. For an example see the supplementary material.

Offsite installation is usually not required. However, if desired it can easily be done on any modern Linux or Mac OSX system. To benefit from all possible diagnostics and plotting features, the installation of the freely available packages TeX, Python and GR-Framework (https://gr-framework.org) is required. A version that works without these packages may be built at the expense of a reduced amount of direct graphical information in the report or online plotting. Suitable Fortran and C compilers, *e.g.* the GNU suite GCC 4.8.5 or newer, are needed in order to build the executable program. The source code is available at https://jugit.fz-juelich.de/nse/drspine.

## Appendix D List of symbols

### d1. List of symbols: main

*t*: time

ω: angular frequency

*Q*: scattering wavevector (length)

dσ/dΩd*E*_f_: double differential scattering cross section

*b*_a_: scattering length of atom

*N*_a_: number of scattering atoms

*E*_i,f_: initial or final neutron energy

*k*_i,f_: initial or final neutron wavevector

*h* = 2πℏ: Planck constant

: cross section prefactor to yield the correct intensity

Φ: volume fraction of labeled molecules in SANS-type description of scattering

Δρ: scattering length density contrast

*S*(*Q*, ω): scattering function

*S*(*Q*, *t*): intermediate scattering function

*F*(*Q*, *t*): normalized intermediate scattering function *S*(*Q*, *t*)/*S*(*Q*)

: uncorrected pixel-bin contribution to *F*(*Q*, *t*)

: resolution-corrected pixel-bin contribution to *S*(*Q*, *t*) or *F*(*Q*, *t*)

λ, λ_0_: neutron wavelength (actual, nominal)

*P*: neutron polarization

*B*: magnetic field

*J*: field integral along a neutron path

*m*_n_: neutron mass

γ_n_: gyromagnetic ratio of the neutron

: resolution (factor)

*I*_Det_: detector (pixel, range of interest) intensity

δ: field integral asymmetry between primary and secondary arm ∝ phase coil current

δ*X*: used as prefix to *X*, δ indicates the (statistical) error of *X*

Δ*J*: field integral difference between paths in the neutron beam

*w*(λ − λ_0_): normalized wavelength distribution of the considered neutron beam

*W*(δ − Δ*J*): distribution of field integral differences in the beam

Λ: width parameter of the λ distribution, Gaussian,

Σ: width parameter of the field integral distribution (inhomogeneity)

η: total efficiency of the polarization analysis

*A*: modification factor due to the λ dependence of ; it is set to 1 in later steps

*p*_*x*_, *p*_*y*_: detector pixel indices

λ_*k*_: nominal wavelength of TOF channel *k*

Δλ: total width of used TOF wavelength frame

: envelope of echo shape

ϕ: phase angle/λ = (δ − δ_0_)γ_n_ *m*_n_/*h*

Ω(ϕ): echo shape as a function of the phase variable ϕ

*z*(ϕ): count rate at phase setting ϕ

: echo amplitude

: average level of echo scan

*I*_*j*_: average detector intensity for TOF channel *j*

*C*: factor between phase-coil current and field integral asymmetry (read from raw data files)

*z*_*i*_ = *z*(ϕ_*i*_): count rate at phase scan point *i* for any pixel bin under consideration

δ*z*_*i*_: statistical error of *z*_*i*_

: transmission of *X* = sample, background *etc.*

: pixel-bin-related absolute calibration factor (future implementation)

### d2. List of symbols: preliminary fitting

*D*_eff_(*Q*): effective diffusion inferred from preliminary stretched exponential fits

β: stretching exponent in stretched exponential fits

*A*(*Q*): amplitude factor in stretched exponential fits

*B*(*Q*): constant base level in stretched exponential fits

### d3. List of symbols: appendix

δ_s,bgr_: statistical error for count rates from sample and background

*t*_s,bgr_: counting time for sample and background

*t*_c_: total (available) counting time

*R*_corr_: resulting count rate after background subtraction

δ_*R*_corr__: error of resulting count rate after background subtraction

: thickness of *X* = sample, background *etc.*
