# Supplementary material for: Efficient data extraction from neutron time-of-flight spin-echo raw data
Source: J Appl Crystallogr. 2019 Aug 29;52(Pt 5):1022–34. doi: 10.1107/S1600576719010847 (PMC6782076; doi:10.1107/S1600576719010847)
Supplement: Supplementary file 1 [file j-52-01022-sup1.pdf]

report20670_707.tex

Jülich Center of Neutron Science, Jülich, Garching, Oak Ridge

DrSpine NSE-data-harvesting Version: 0.42-f1a9f38 (build on Mar 13 2019 10:48:26, GNU Fortran)
Report problems to p.zolnierczuk@fz-juelich.de and/or m.monkenbusch@fz-juelich.de

SDS5wD2O_"SDS_with_NaCl_in_D2O_0.05w_in_refpos,_room_temp"

Macro call:

Content of the macro as executed here can be found at the end of this report!

Table 1: Overview of loaded data files

num	np	name	2θ	$\lambda_{1,n}$ /Å	J_{eff}/milliTm	T/K	up	down	role	mode
20666	20	actC2mm Sat Jun	3.67°	8.00 -8.00	0.9925 - 939.57	0.0000	99.62	14.29	ref	standard
20668	20	actC2mm Sat Jun	5.14°	8.00 -8.00	0.9928 - 939.57	0.0000	34.79	4.771	ref	standard
20669	20	actC2mm Sun Jun	7.29°	8.00 -8.00	0.9932 - 939.55	0.0000	13.11	1.871	ref	standard
20676	18	actC4mm Sun Jun	11.0°	8.00 -8.00	0.9940 - 730.77	0.0000	25.29	2.843	ref	standard
20670	16	SDS5wD2O Sun Ju	3.69°	8.00 -8.00	0.9925 - 730.77	0.0000	51.53	5.682	sam	standard
20672	4	SDS5wD2O Sun Ju	5.13°	8.00 -8.00	0.9928 - 10.388	0.0000	85.66	9.053	sam	standard
20673	16	SDS5wD2O Sun Ju	5.13°	8.00 -8.00	0.9928 - 730.77	0.0000	84.54	8.979	sam	standard
20674	16	SDS5wD2O Sun Ju	7.31°	8.00 -8.00	0.9932 - 730.76	0.0000	70.83	8.155	sam	standard
20675	13	SDS5wD2O Sun Ju	11.0°	8.00 -8.00	0.9940 - 417.56	0.0000	19.36	2.773	sam	standard

Remarks:
Up and down intensity are given in terms of TOF summed, monitor(?) normalized counts in the central binned pixel, intensity units scaled by 1.000×10^3

Binning:
Detector area to 8×8 pixels , λ-bins from 1 TOF channles to 1 λ-bins

Table 2: Active Reduction Parameters:

Parameter	symbol	udf-name	value	remark
minimum counts	c_{min}	r.min_counts	100.00	use only pixels with $c > c_{\text{min}}$
minimum sigma	σ_{min}	r.min_sigma	0.0500	use only pixels with variation larger than sigma
min. average	B_{min}	r.ave_min	0.0000	min. echo average value
max. average dev.	$\Delta B = \frac{2B}{U+D} - 1$	r.ave_maxdev	0.2500	max. echo average deviation from U/D average
min resolution (fqt)	Fqt min	r.fqt_min	0.2000	minimum pixel fqt value (res)
max resolution (fqt) error	max $\sigma(Fqt)$	r.fqt_maxsigma	0.8000	maximum error for pixel fqt value (res)
phase tolerance	ϵ	r.phase_tol	1.0000×10^{-9}	echo phase fitting tolerance
phase limits	$\Delta\phi_{\text{max}}$	r.phase_maxdev	1.2700	echo phase fitting limits [rad]
phase shift limits	$\delta\phi_{\text{min}}$	r.phase_minoff	1.0000×10^{-4}	symmetry phase offset minimum
phase smoothing step	$\Delta\phi_{\text{smooth}}$	r.phase_cstep	1.5700	symmetry phase correction step for smoothing [rad]
max chi**2	χ^2_{max}	r.max_chisq	1.0000×10^3	max. chisquare for echo group fit
intensity factor	f	-	1.0000×10^3	all up, down, average values are scaled by it.
Postcollection q-group	Δq	r.postcoll_qcat	2.5000×10^7	Width for postcollection q-grouping

---

Explanations:

- **num** = run number of *.echo file
- **np** = number of field integral settings.
- 2θ = nominal scattering angle (detector center).
- $\lambda_{1,n}$ = minimal and maximal wavelength in the selected frame.
- J_{eff} = min, max effective field intergral settings.
- T/**K** = sample temperature (reading of sample T-sensor).
- **up** = spin-up intensity at central detector pixel.

- **down** = spin-down intensity at central detector pixel.
- **role** = assigned role as reference, sample or background..
- **mode** = normal=std NSE mode, or shorty for very small field integrals.
- Δq = q-banding width for q-groups in the dtr output files.

..for the tables shown below:

- **#** = number of τ (J_0) setting of that scan
- **J_0 /mTm** = effective field integral in milli Tesla meter
- **theta** = 2θ the nominal scattering angle (detector center).
- **nt** = number of λ-bins to cover the TOF-frame
- **npic** = total number of detector pixels
- **nact** = total number of usable (survived) detector pixels
- **average0** = average intensity at central pixel
- $\langle$**average**$\rangle$ = average intensity over active detector area (per pixel)
- $\langle$**amplitu**$\rangle$ = average echo amplitude over active detector area (per pixel)
- **dj0/muTm** = symmetry deviation ΔJ of central pixel in μTm
- **dja/muTm** = symmetry deviation ΔJ of average pixel in μTm
- **djv/muTm** = variance of symmetry deviation ΔJ over active detector in μTm
- **lam1** = λ_{min}/Å of wavelength bin
- **lam2** = λ_{max}/Å of wavelength bin
- **ref** = run number of matched reference (0=N.A.)
- **pt** = matching scan point of reference
- **bgr** = run number of matched background (0=N.A.)
- **pt** = matching scan point of background

PIXEL STATUS short letter- and (color- codes) in portrait plots:

- **R** : no resolution (1000)
- **B** : no background (2000)
- **O** : offset failed (4000)
- **F** : fit failed (10)
- **Q** : fit - chi2 too large (20)
- **l** : fit - amplitude too small (40)
- **N** : fit - negative amplitude (100)
- **C** : not enough counts (1)
- **a** : raw amplitude (2)(If abundand: reduce *minimum sigma* σ_{min} ; **r.min.sigma**)
- **A** : average is out of bounds (4)
- **U** : average is not consistent with up/down (8)

Detailed list of loaded run files *(report details)*

20666 m20666.echo **actC2mm Sat Jun 23 18:37:55 2018** actC2mm "actC 2mm at

reference 20 pnts

actC2mm "actC 2mm at sampos in spongebob"

actC2mm "actC 2mm at sampos in spongebob"

matching reference: 20666
matching background: –
wavelength $\lambda = 8.0000$ Å
$Q/\text{Å}^{-1} = 0.0503$ (2θ =3.6694 ° Ψ =−88.177 °)
Up = 99.617 Down= 14.289
Normalized echo amplitude (J_{min}, J_{max})
$(\tau_{min} = 0.0947$ ns $\cdots$ $\tau_{max} = 89.647$ ns) at detector center: 0.9929 0.6594

#	J0/muTm	theta	nt	npic	nact	average0	<average>	<amplitu>	dj0/muTm	dja/muTm	djv/muTm	lam1/A	lam2/A	ref	pt	bgr pt	distribution: dj-count per 1muTm
1	992.49	3.67	1	64	55	5.73689E+01	4.39137E+01	3.27160E+01	-0.1060	0.4066	1.2311	8.000	8.000	20666	1	-- --	| 0 0 0 0 0 9141113 8 0 0 0 0 0|
2	2555.64	3.67	1	64	55	5.73661E+01	4.37655E+01	3.17700E+01	2.0418	0.9629	0.9103	8.000	8.000	20666	2	-- --	| 0 0 0 0 0 4122018 1 0 0 0 0 0|
3	5167.14	3.67	1	64	55	5.73920E+01	4.38151E+01	3.05228E+01	2.0377	-0.6814	1.5604	8.000	8.000	20666	3	-- --	| 0 0 0 0 1101311 7 9 4 0 0 0 0|
4	10387.80	3.67	1	64	55	5.77502E+01	4.38357E+01	2.89909E+01	2.0073	-2.6373	2.5767	8.000	8.000	20666	4	-- --	| 0 0 1 2 7 810 4 6 5 5 5 2 0 0|
5	20828.60	3.67	1	64	55	5.76188E+01	4.38942E+01	2.62313E+01	2.3638	-4.3434	3.7086	8.000	8.000	20666	5	-- --	| 1 2 1 7 210 6 4 3 2 6 2 2 4 3|
6	31268.85	3.67	1	64	55	5.78067E+01	4.39953E+01	2.36736E+01	2.8559	-5.0312	4.3748	8.000	8.000	20666	6	-- --	| 3 2 5 2 5 5 8 3 2 3 5 2 2 2 6|
7	52149.95	3.67	1	64	53	5.79779E+01	4.53609E+01	2.03504E+01	3.8519	-5.2373	5.5050	8.000	8.000	20666	7	-- --	| 8 3 1 4 5 1 4 2 2 2 4 6 1 2 8|
8	73030.30	3.67	1	64	55	5.76254E+01	4.39888E+01	1.86736E+01	4.1990	-5.4117	6.2483	8.000	8.000	20666	8	-- --	|12 1 3 3 5 3 1 1 2 2 2 5 1 113|
9	104351.50	3.67	1	64	51	5.78409E+01	4.69354E+01	1.85230E+01	3.7853	-7.4705	6.6768	8.000	8.000	20666	9	-- --	|11 2 3 2 2 5 3 2 0 1 3 2 3 210|
10	156553.50	3.67	1	64	48	5.79636E+01	4.94329E+01	1.84769E+01	3.6255	-7.2474	6.5432	8.000	8.000	20666	10	-- --	| 7 5 3 3 3 2 3 0 1 2 3 3 3 1 9|
11	260957.00	3.67	1	64	49	5.79652E+01	4.85365E+01	1.88011E+01	2.9301	-7.4011	6.5453	8.000	8.000	20666	11	-- --	| 6 2 3 8 1 5 0 2 1 0 6 3 2 1 9|
12	313156.00	3.67	1	64	49	5.79658E+01	4.85239E+01	1.95152E+01	2.9319	-5.6866	5.6910	8.000	8.000	20666	12	-- --	| 5 3 3 4 6 0 2 2 1 5 7 1 0 010|
13	365357.00	3.67	1	64	47	5.82870E+01	4.96845E+01	2.09099E+01	2.9646	-4.8782	5.6239	8.000	8.000	20666	13	-- --	| 5 1 4 2 3 4 2 2 3 5 4 3 0 2 7|
14	417558.00	3.67	1	64	47	5.82872E+01	4.87499E+01	2.11791E+01	2.8250	-3.3473	5.3218	8.000	8.000	20666	14	-- --	| 5 1 4 3 1 2 2 4 5 4 2 4 2 2 6|
15	469759.00	3.67	1	64	46	5.85783E+01	5.06609E+01	2.18474E+01	2.7424	-2.0575	4.9074	8.000	8.000	20666	15	-- --	| 5 2 4 1 3 1 2 2 5 6 1 4 3 5 2|
16	521960.00	3.67	1	64	46	5.84757E+01	5.09441E+01	2.14316E+01	2.7259	-1.2144	5.4557	8.000	8.000	20666	16	-- --	| 7 2 2 3 1 2 1 1 5 6 1 5 4 3 3|
17	626364.00	3.67	1	64	43	5.87149E+01	4.87692E+01	2.02147E+01	2.5790	1.8025	6.6004	8.000	8.000	20666	17	-- --	| 7 1 3 2 4 1 1 3 4 3 3 2 2 1 6|
18	730765.50	3.67	1	64	34	5.88602E+01	5.36053E+01	2.15563E+01	2.4903	2.4282	5.1992	8.000	8.000	20666	18	-- --	| 4 2 1 1 3 2 0 3 1 8 2 1 0 2 4|
19	835167.50	3.67	1	64	29	5.90731E+01	5.49400E+01	2.09141E+01	2.2479	3.4940	5.4596	8.000	8.000	20666	19	-- --	| 3 3 1 2 1 0 2 1 2 3 5 1 1 1 3|
20	939569.50	3.67	1	64	27	5.92515E+01	5.19553E+01	1.80749E+01	2.0020	4.1719	5.2117	8.000	8.000	20666	20	-- --	| 3 2 2 2 1 2 0 1 1 3 2 1 4 1 2|

Table 3: Applied phase offsets and $10^6 \times \chi^2$ of aux. parameters (B_x, B_y,.. And Δ = difference ref-sample in 10^{-3} units (B:milliGauss).

nt	$\Delta J/\mu$Tm	$\simeq \Delta\Phi$	Bx	By	Bz	B2x	B2y	B2z	B1x	B1y	B1z
1	0.000	0.0000°	2.2	1.3	0.0	0.1	0.3	0.0	0.1	0.1	0.1
2	0.000	0.0000°	0.1	0.0	0.0	0.0	0.1	0.0	0.1	0.1	0.0
3	0.000	0.0000°	0.2	0.0	0.0	0.0	0.0	0.0	0.1	0.1	0.0
4	0.000	0.0000°	0.6	0.0	0.0	0.0	0.0	0.0	0.1	0.0	0.1
5	0.000	0.0000°	0.8	0.0	0.0	0.2	0.0	0.0	0.0	0.1	0.1
6	0.000	0.0000°	1.0	0.0	0.0	0.2	0.0	0.0	0.1	0.0	0.1
7	0.000	0.0000°	2.0	0.0	0.0	0.0	0.0	0.1	0.1	0.0	0.1
8	0.000	0.0000°	1.7	0.0	0.1	0.2	0.1	0.1	0.0	0.1	0.1
9	0.000	0.0000°	1.5	1.1	0.0	0.2	0.2	0.0	0.1	0.2	0.1
10	0.000	0.0000°	1.5	0.0	0.0	0.0	0.1	0.0	0.1	0.1	0.1
11	0.000	0.0000°	0.9	0.0	0.0	0.0	0.1	0.0	0.1	0.1	0.1
12	0.000	0.0000°	1.3	0.2	0.0	0.1	0.1	0.1	0.1	0.1	0.1
13	0.000	0.0000°	1.9	1.1	0.0	0.1	0.4	0.1	0.1	0.1	0.1
14	0.000	0.0000°	0.7	0.0	0.0	0.1	0.1	0.0	0.1	0.1	0.1
15	0.000	0.0000°	0.4	0.0	0.0	0.1	0.1	0.2	0.1	0.1	0.1
16	0.000	0.0000°	0.4	0.0	0.0	0.1	0.1	0.1	0.1	0.1	0.1
17	0.000	0.0000°	0.1	0.0	0.0	0.1	0.1	0.0	0.1	0.1	0.1
18	0.000	0.0000°	1.1	1.0	0.0	0.0	0.3	0.0	0.1	0.1	0.1
19	0.000	0.0000°	0.1	0.0	0.0	0.0	0.1	0.1	0.1	0.1	0.1
20	0.000	0.0000°	0.1	0.0	0.1	0.0	0.1	0.1	0.1	0.1	0.1
Ave	0.0000	0.0000°	-	-	-	-	-	-	-	-	-
Var	0.0000	0.0000°	-	-	-	-	-	-	-	-	-

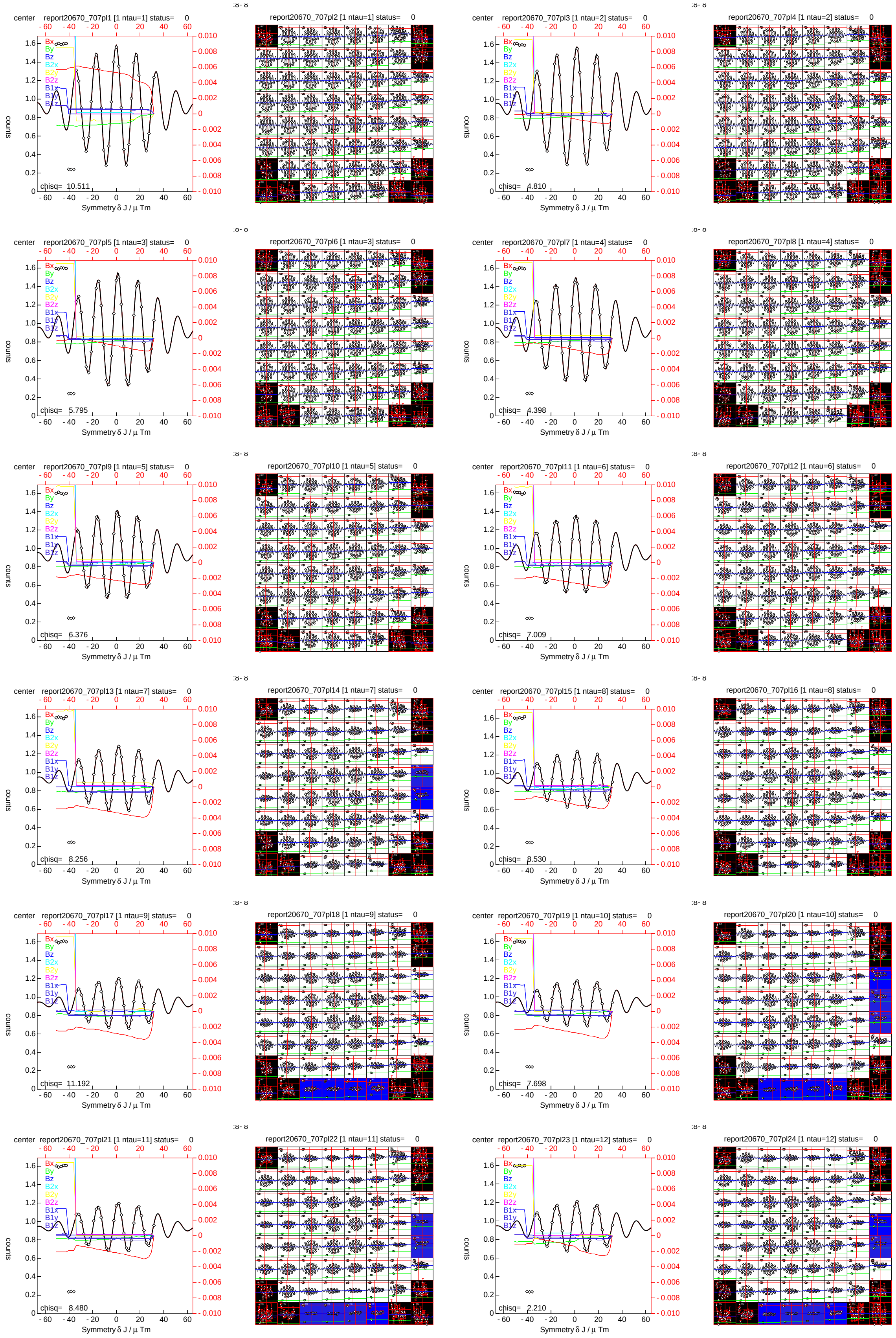

Figure 1: Center phase scan plots. Dashed red line + diamonds: scaled reference.

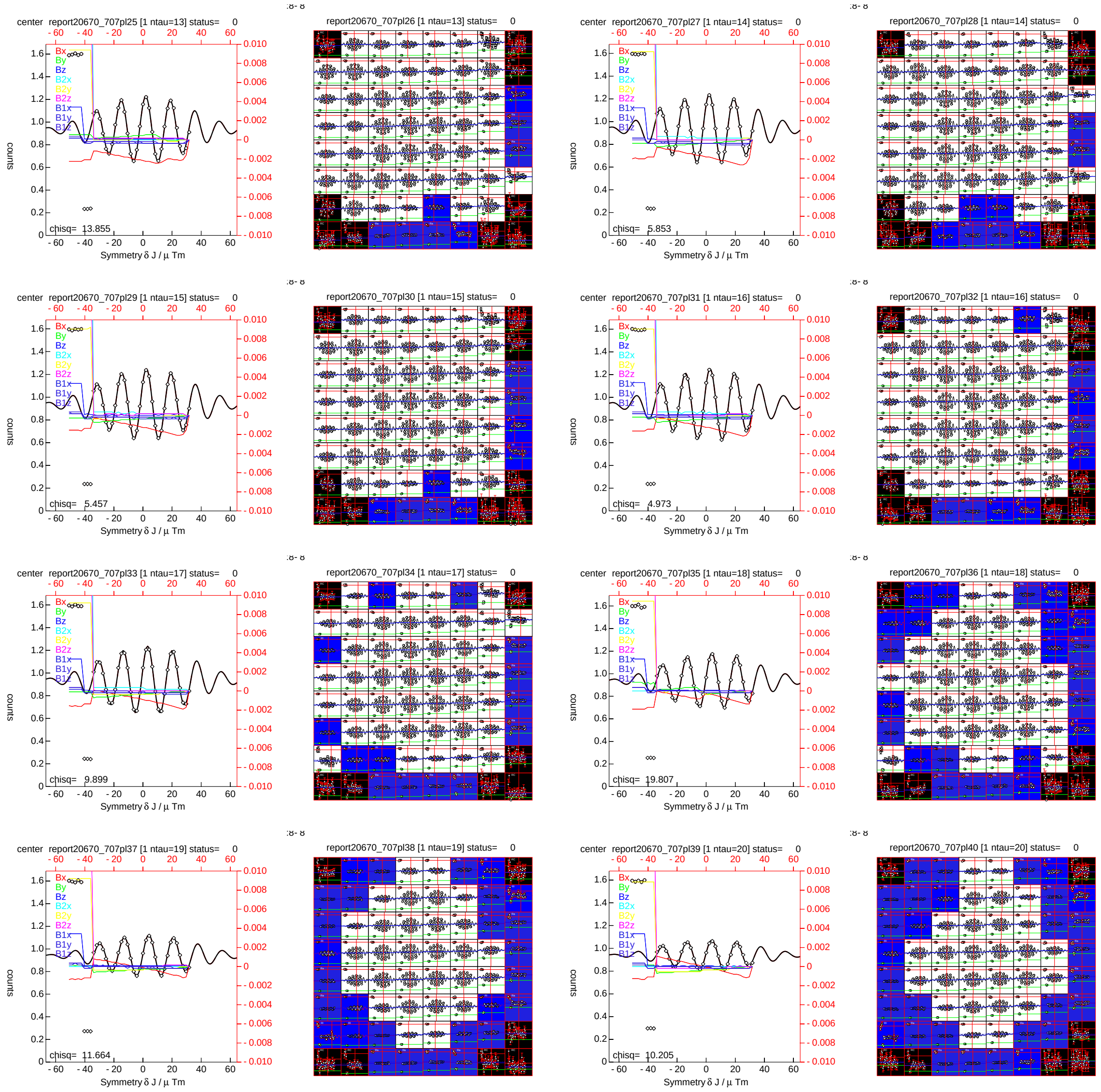

Figure 2: Continuation: Center phase scan plots. Dashed red line + diamonds: scaled reference.

20668 m20668.echo reference 20 pnts

actC2mm Sat Jun 23 23:35:32 2018 actC2mm "actC 2mm at

actC2mm "actC 2mm at sampos in spongebob"

actC2mm "actC 2mm at sampos in spongebob"

matching reference: 20668
matching background: –
wavelength $\lambda = 8.0000$ Å

$Q/\text{Å}^{-1} = 0.0704$ ($2\theta = 5.1361$ ° $\Psi = -87.440$ °)

Up = 34.794 Down= 4.7706

Normalized echo amplitude (J_{min}, J_{max})

$(\tau_{min} = 0.0947$ ns $\cdots$ $\tau_{max} = 89.647$ ns) at detector center: 0.9965 0.6495

#	J0/muTm	theta	nt	npic	nact	average0	<average>	<amplitu>	dj0/muTm	dja/muTm	djv/muTm	lam1/A	lam2/A	ref	pt	bgr	pt	distribution: dj-count per 1muTm
1	992.78	5.14	1	64	53	1.99043E+01	1.46839E+01	1.07440E+01	-0.3326	0.2739	1.2488	8.000	8.000	20668	1	--	--	| 0 0 0 0 0 9131111 9 0 0 0 0 0|
2	2555.62	5.14	1	64	53	1.97367E+01	1.46228E+01	1.04258E+01	2.0853	1.1369	0.8146	8.000	8.000	20668	2	--	--	| 0 0 0 0 0 3122117 0 0 0 0 0 0|
3	5167.15	5.14	1	64	53	1.98578E+01	1.47038E+01	1.00479E+01	1.9238	-0.6020	1.4311	8.000	8.000	20668	3	--	--	| 0 0 0 0 0 91410 9 9 2 0 0 0 0|
4	10387.80	5.14	1	64	53	1.99443E+01	1.47013E+01	9.42770E+00	1.8498	-2.5585	2.4564	8.000	8.000	20668	4	--	--	| 0 0 1 1 6 910 5 5 7 2 6 1 0 0|
5	20828.60	5.14	1	64	53	1.98512E+01	1.47150E+01	8.46855E+00	2.1668	-4.2937	3.6218	8.000	8.000	20668	5	--	--	| 1 1 2 6 4 9 6 3 3 4 3 2 3 4 2|
6	31268.85	5.14	1	64	53	1.99540E+01	1.47073E+01	7.61948E+00	2.6623	-4.9788	4.3291	8.000	8.000	20668	6	--	--	| 2 2 5 3 4 5 7 4 1 3 5 2 2 2 6|
7	52149.95	5.14	1	64	52	1.98128E+01	1.49183E+01	6.53963E+00	3.7189	-5.0996	5.5841	8.000	8.000	20668	7	--	--	| 8 3 1 3 5 4 1 2 2 3 7 2 1 1 9|
8	73030.35	5.14	1	64	53	1.99486E+01	1.46947E+01	6.01409E+00	4.0789	-5.1745	6.5089	8.000	8.000	20668	8	--	--	|11 1 3 5 3 3 1 0 2 3 3 3 0 213|
9	104351.50	5.14	1	64	50	2.01081E+01	1.53936E+01	5.80079E+00	3.8648	-6.9235	6.6429	8.000	8.000	20668	9	--	--	|10 2 3 3 4 3 2 1 0 2 3 3 3 011|
10	156553.50	5.14	1	64	48	2.00913E+01	1.59377E+01	5.69090E+00	3.5699	-6.2763	7.3972	8.000	8.000	20668	10	--	--	|12 2 3 2 2 3 0 0 2 4 2 3 0 112|
11	260957.00	5.14	1	64	46	1.99715E+01	1.58463E+01	6.01734E+00	3.1360	-6.6601	7.1142	8.000	8.000	20668	11	--	--	| 7 3 3 4 3 1 2 1 0 3 4 4 1 010|
12	313156.00	5.14	1	64	46	1.99806E+01	1.59175E+01	6.28476E+00	2.9362	-6.3225	6.4299	8.000	8.000	20668	12	--	--	| 5 2 2 5 5 1 2 2 1 1 6 4 1 0 9|
13	365357.00	5.14	1	64	45	2.01129E+01	1.61484E+01	6.65269E+00	2.9559	-4.2853	5.8699	8.000	8.000	20668	13	--	--	| 5 4 1 3 4 1 1 4 3 3 6 0 1 2 7|
14	417558.00	5.14	1	64	46	2.01473E+01	1.58687E+01	6.72008E+00	2.7517	-3.5454	5.9673	8.000	8.000	20668	14	--	--	| 5 4 3 1 1 3 2 3 5 3 4 2 2 2 6|
15	469759.00	5.14	1	64	44	2.01701E+01	1.64805E+01	7.07439E+00	2.7208	-2.3521	4.9182	8.000	8.000	20668	15	--	--	| 4 4 2 1 2 1 4 1 6 4 2 3 5 2 3|
16	521960.00	5.14	1	64	44	2.02780E+01	1.61395E+01	6.93977E+00	2.6683	-0.8375	5.4906	8.000	8.000	20668	16	--	--	| 6 3 1 2 2 1 1 4 6 3 1 6 1 3 4|
17	626364.00	5.14	1	64	40	2.01450E+01	1.65047E+01	6.95806E+00	2.6409	1.2594	5.7966	8.000	8.000	20668	17	--	--	| 6 0 5 2 2 1 2 1 5 3 3 3 2 2 3|
18	730766.50	5.14	1	64	34	2.04302E+01	1.77566E+01	7.11444E+00	2.4163	3.0952	5.0262	8.000	8.000	20668	18	--	--	| 4 1 1 3 2 1 2 2 7 3 2 0 2 1 3|
19	835168.50	5.14	1	64	28	2.05208E+01	1.87455E+01	7.20477E+00	2.2389	3.5110	5.3087	8.000	8.000	20668	19	--	--	| 4 2 0 3 0 0 2 1 3 5 3 1 0 1 3|
20	939570.50	5.14	1	64	25	2.05438E+01	1.84340E+01	6.54465E+00	2.0678	4.1064	5.1959	8.000	8.000	20668	20	--	--	| 2 3 1 2 1 2 0 1 1 2 3 1 3 1 2|

Table 4: Applied phase offsets and $10^6 \times \chi^2$ of aux. parameters (B_x, B_y,.. And Δ = difference ref-sample in 10^{-3} units (B:milliGauss).

nt	$\Delta J/\mu$Tm	$\simeq \Delta\Phi$	Bx	By	Bz	B2x	B2y	B2z	B1x	B1y	B1z
1	0.000	0.0000°	**0.4**	**0.0**	**0.0**	**0.0**	**0.0**	**0.0**	**0.1**	**0.1**	**0.1**
2	0.000	0.0000°	**1.3**	**0.9**	**0.0**	**0.1**	**0.1**	**0.0**	**0.1**	**0.1**	**0.1**
3	0.000	0.0000°	**0.1**	**0.0**	**0.0**	**0.0**	**0.0**	**0.0**	**0.0**	**0.1**	**0.1**
4	0.000	0.0000°	**0.3**	**0.0**	**0.0**	**0.0**	**0.0**	**0.0**	**0.1**	**0.1**	**0.0**
5	0.000	0.0000°	**0.9**	**0.0**	**0.0**	**0.0**	**0.0**	**0.0**	**0.1**	**0.1**	**0.1**
6	0.000	0.0000°	**0.8**	**0.0**	**0.0**	**0.2**	**0.0**	**0.3**	**0.1**	**0.1**	**0.0**
7	0.000	0.0000°	**1.7**	**0.4**	**0.0**	**0.0**	**0.1**	**0.1**	**0.0**	**0.1**	**0.1**
8	0.000	0.0000°	**1.4**	**1.0**	**0.0**	**0.1**	**0.2**	**0.0**	**0.1**	**0.1**	**0.1**
9	0.000	0.0000°	**1.3**	**0.0**	**0.0**	**0.0**	**0.0**	**0.1**	**0.1**	**0.1**	**0.1**
10	0.000	0.0000°	**1.3**	**0.0**	**0.0**	**0.0**	**0.0**	**0.1**	**0.0**	**0.1**	**0.1**
11	0.000	0.0000°	**0.9**	**0.0**	0.0	**0.1**	**0.1**	**0.0**	**0.1**	**0.1**	**0.1**
12	0.000	0.0000°	**0.5**	**0.2**	**0.0**	**0.1**	**0.1**	**0.0**	**0.1**	**0.0**	**0.1**
13	0.000	0.0000°	**1.2**	**1.0**	**0.0**	**0.0**	**0.4**	**0.1**	**0.1**	**0.1**	**0.1**
14	0.000	0.0000°	**0.4**	**0.0**	**0.0**	**0.1**	**0.1**	**0.1**	**0.1**	**0.1**	**0.1**
15	0.000	0.0000°	**0.3**	**0.0**	**0.0**	**0.1**	**0.1**	**0.0**	**0.1**	**0.1**	**0.1**
16	0.000	0.0000°	**0.2**	**0.0**	**0.0**	**0.1**	**0.1**	**0.1**	**0.1**	**0.1**	**0.1**
17	0.000	0.0000°	**2.9**	**3.0**	**0.0**	**0.4**	**0.6**	**0.1**	**0.1**	**0.1**	**0.1**
18	0.000	0.0000°	**0.1**	**0.0**	**0.0**	**0.0**	**0.1**	**0.1**	**0.1**	**0.1**	**0.1**
19	0.000	0.0000°	**0.0**	**0.0**	**0.0**	**0.0**	**0.1**	**0.1**	**0.1**	**0.1**	**0.1**
20	0.000	0.0000°	**3.2**	**2.9**	**0.0**	**0.0**	**0.9**	**0.1**	**0.1**	**0.1**	**0.1**
Ave	0.0000	0.0000°	-	-	-	-	-	-	-	-	-
Var	0.0000	0.0000°	-	-	-	-	-	-	-	-	-

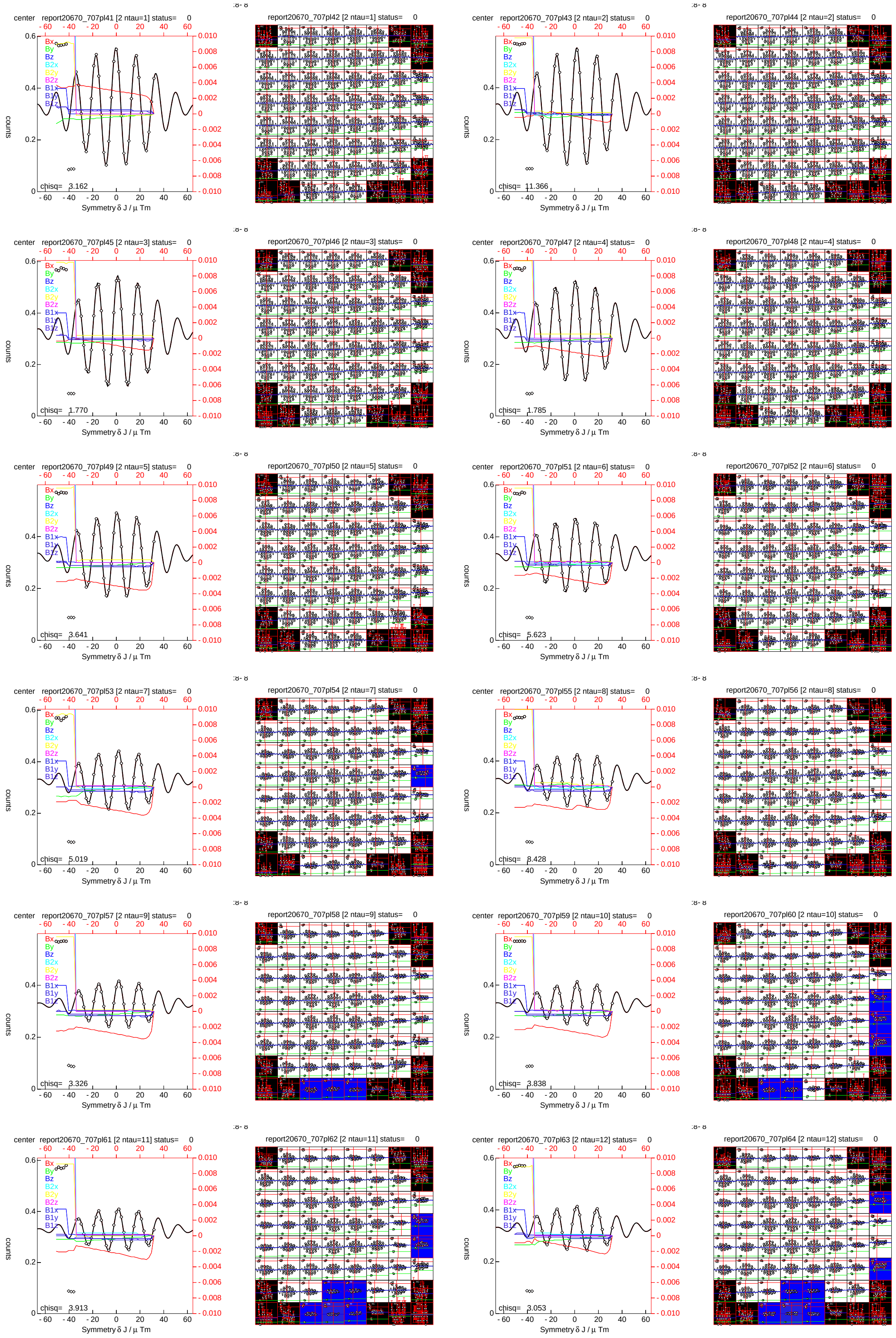

Figure 3: Center phase scan plots. Dashed red line + diamonds: scaled reference.

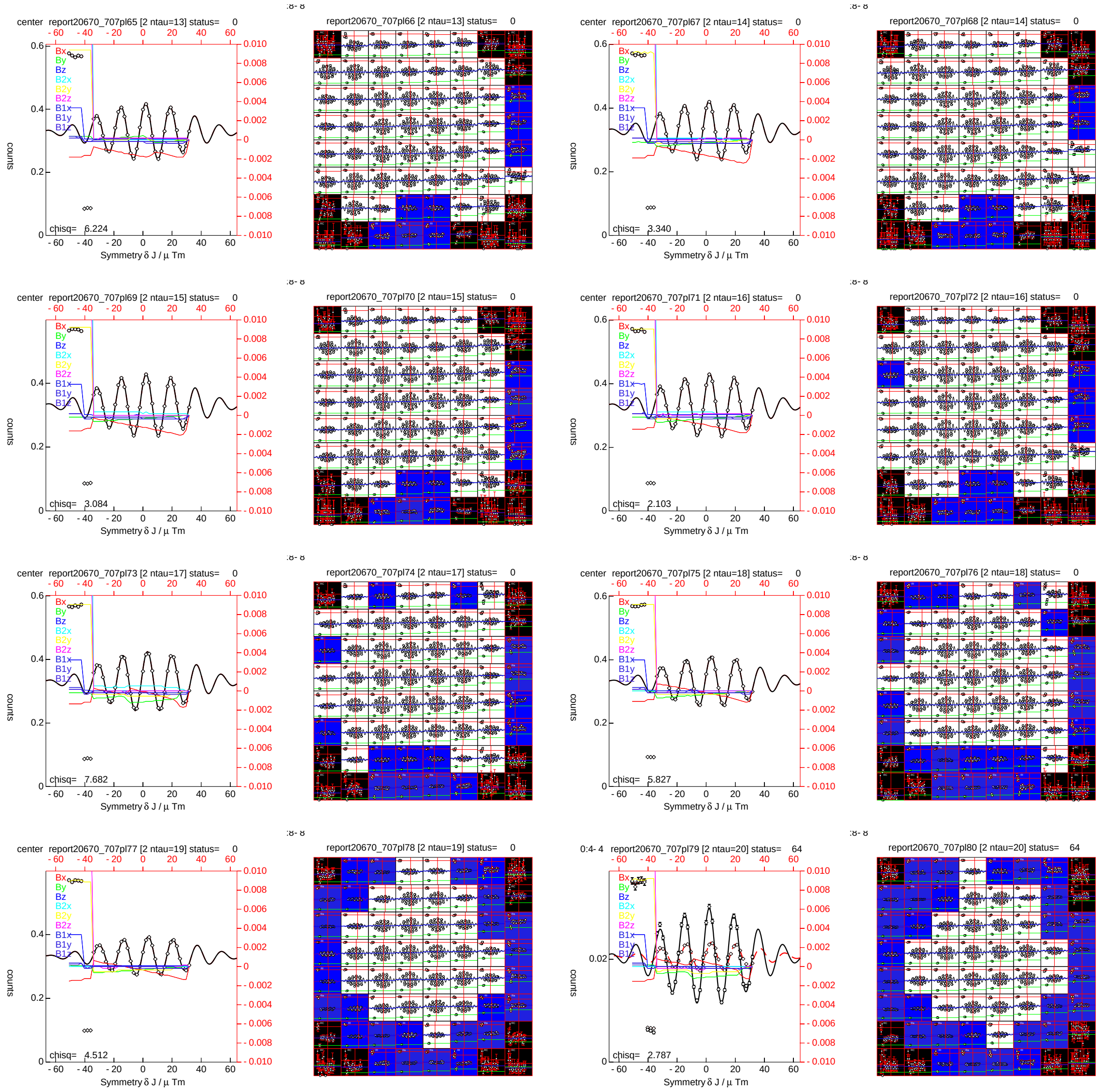

Figure 4: Continuation: Center phase scan plots. Dashed red line + diamonds: scaled reference.

20669 m20669.echo reference 20 pnts

actC2mm Sun Jun 24 04:12:03 2018

actC2mm "actC 2mm at

actC2mm "actC 2mm at sampos in spongebob"

actC2mm "actC 2mm at sampos in spongebob"

matching reference: 20669
matching background: –
wavelength $\lambda = 8.0000$ Å

$Q/\text{Å}^{-1} = 0.0999$ ($2\theta = 7.2949$ ° $\Psi = -86.347$ °)

Up = 13.112 Down= 1.8711

Normalized echo amplitude (J_{min}, J_{max})

(τ_{min} = 0.0948 ns $\cdots$ τ_{max} = 89.645 ns) at detector center: 0.9859 0.6456

#	J0/muTm	theta	nt	npic	nact	average0	<average>	<amplitu>	dj0/muTm	dja/muTm	djv/muTm	lam1/A	lam2/A	ref	pt	bgr	pt	distribution: dj-count per 1muTm
1	993.23	7.29	1	64	51	7.56703E+00	5.80849E+00	4.02429E+00	-0.2979	0.3045	1.2166	8.000	8.000	20669	1	--	--	| 0 0 0 0 0 6141212 7 0 0 0 0 0|
2	2555.59	7.29	1	64	51	7.63509E+00	5.82369E+00	3.93867E+00	2.0744	1.3331	0.7488	8.000	8.000	20669	2	--	--	| 0 0 0 0 0 1132215 0 0 0 0 0 0|
3	5167.16	7.29	1	64	51	7.53346E+00	5.83345E+00	3.78093E+00	2.2272	-0.0501	1.3182	8.000	8.000	20669	3	--	--	| 0 0 0 0 1 6131211 8 0 0 0 0 0|
4	10387.85	7.29	1	64	51	7.57085E+00	5.81040E+00	3.54020E+00	2.0026	-1.7947	3.0613	8.000	8.000	20669	4	--	--	| 0 0 1 3 411 6 6 6 4 5 4 0 0 1|
5	20828.60	7.29	1	64	51	7.60412E+00	5.81805E+00	3.16141E+00	2.3073	-3.8654	3.5445	8.000	8.000	20669	5	--	--	| 1 0 3 5 6 6 6 3 3 5 3 2 3 4 1|
6	31268.85	7.29	1	64	51	7.55660E+00	5.82380E+00	2.86311E+00	2.8171	-4.2989	4.6017	8.000	8.000	20669	6	--	--	| 1 4 4 3 5 7 3 2 3 3 5 1 1 4 5|
7	52150.05	7.29	1	64	49	7.58777E+00	5.98417E+00	2.52157E+00	3.8811	-4.9818	5.3679	8.000	8.000	20669	7	--	--	| 7 3 1 2 5 4 2 2 1 4 5 3 1 1 8|
8	73030.40	7.29	1	64	51	7.61632E+00	5.82331E+00	2.23622E+00	4.2608	-5.6167	6.2599	8.000	8.000	20669	8	--	--	|10 3 1 5 4 2 2 1 0 2 3 4 2 111|
9	104351.50	7.29	1	64	49	7.60200E+00	6.00247E+00	2.12671E+00	4.0669	-4.9047	8.4014	8.000	8.000	20669	9	--	--	|12 4 3 2 2 1 0 2 3 3 3 0 1 112|
10	156553.50	7.29	1	64	45	7.49917E+00	6.28379E+00	2.12162E+00	3.5711	-7.2253	6.8889	8.000	8.000	20669	10	--	--	| 9 3 2 3 3 2 2 0 0 2 4 2 3 010|
11	260957.50	7.29	1	64	47	7.58520E+00	6.07123E+00	2.11135E+00	3.1873	-6.8802	7.1221	8.000	8.000	20669	11	--	--	| 9 3 1 6 2 1 1 2 0 1 6 3 1 110|
12	313156.00	7.29	1	64	43	7.61335E+00	6.18189E+00	2.34544E+00	3.0284	-5.3494	7.5802	8.000	8.000	20669	12	--	--	| 7 3 1 4 2 1 2 1 3 4 4 1 0 1 9|
13	365357.00	7.29	1	64	44	7.65363E+00	6.25503E+00	2.42223E+00	2.9394	-4.4580	5.7699	8.000	8.000	20669	13	--	--	| 5 2 3 3 3 1 2 3 4 2 6 0 1 1 8|
14	417557.50	7.29	1	64	44	7.69668E+00	6.25870E+00	2.50805E+00	2.7731	-3.3389	5.5160	8.000	8.000	20669	14	--	--	| 6 2 3 0 3 3 0 5 3 4 4 2 1 2 6|
15	469758.00	7.29	1	64	45	7.74077E+00	6.13989E+00	2.47060E+00	2.7654	-3.2366	5.6990	8.000	8.000	20669	15	--	--	| 6 1 4 1 2 2 2 2 2 6 4 1 4 2 6|
16	521958.00	7.29	1	64	46	7.75281E+00	6.15183E+00	2.43953E+00	2.7491	-1.5086	6.9071	8.000	8.000	20669	16	--	--	| 7 3 2 0 1 2 1 3 4 6 1 4 3 3 6|
17	626359.00	7.29	1	64	40	7.76297E+00	6.32913E+00	2.54453E+00	2.6528	1.7007	6.5086	8.000	8.000	20669	17	--	--	| 5 4 3 1 1 2 1 4 3 3 3 2 4 0 4|
18	730756.50	7.29	1	64	35	7.81244E+00	6.71041E+00	2.57479E+00	2.5089	1.7449	7.5527	8.000	8.000	20669	18	--	--	| 5 1 2 2 1 2 1 0 4 4 3 2 1 2 5|
19	835151.50	7.29	1	64	30	7.82381E+00	7.18246E+00	2.60827E+00	2.4020	3.4736	6.0583	8.000	8.000	20669	19	--	--	| 4 2 0 2 1 1 3 0 3 5 2 2 0 1 4|
20	939545.00	7.29	1	64	26	7.77473E+00	7.08164E+00	2.41763E+00	2.0578	2.9208	7.3365	8.000	8.000	20669	20	--	--	| 3 0 2 2 2 1 2 0 0 2 1 3 4 1 3|

Table 5: Applied phase offsets and $10^6 \times \chi^2$ of aux. parameters (B_x, B_y,.. And Δ = difference ref-sample in 10^{-3} units (B:milliGauss).

nt	$\Delta J/\mu$Tm	$\simeq \Delta\Phi$	Bx	By	Bz	B2x	B2y	B2z	B1x	B1y	B1z
1	0.000	0.0000°	**0.4**	**0.0**	**0.0**	**0.1**	**0.0**	**0.0**	**0.1**	**0.0**	**0.1**
2	0.000	0.0000°	**0.0**	**0.0**	**0.0**	**0.2**	**0.0**	**0.1**	**0.0**	**0.1**	**0.1**
3	0.000	0.0000°	**1.2**	**1.4**	**0.0**	**0.1**	**0.4**	**0.0**	**0.1**	**0.1**	**0.0**
4	0.000	0.0000°	**0.3**	**0.0**	**0.0**	**0.1**	**0.1**	0.0	**0.0**	**0.0**	**0.1**
5	0.000	0.0000°	**0.5**	**0.0**	**0.0**	**0.0**	**0.0**	**0.1**	**0.1**	**0.1**	**0.0**
6	0.000	0.0000°	**0.6**	**0.0**	**0.0**	**0.1**	**0.0**	**0.0**	**0.1**	**0.1**	**0.0**
7	0.000	0.0000°	**1.1**	**0.0**	**0.0**	**0.0**	**0.1**	**0.3**	**0.0**	**0.1**	**0.1**
8	0.000	0.0000°	**1.4**	**1.6**	**0.0**	**0.1**	**0.3**	**0.0**	**0.1**	**0.0**	**0.1**
9	0.000	0.0000°	**1.2**	**0.0**	**0.1**	**0.1**	**0.0**	**0.0**	**0.1**	**0.3**	**0.1**
10	0.000	0.0000°	**0.8**	**0.0**	**0.0**	**0.1**	**0.1**	**0.0**	**0.1**	**0.0**	**0.1**
11	0.000	0.0000°	**0.6**	**0.0**	**0.0**	**0.1**	**0.1**	**0.1**	**0.1**	**0.1**	**0.0**
12	0.000	0.0000°	**1.6**	**0.9**	**0.1**	**0.1**	**0.2**	**0.2**	**0.2**	**0.0**	**0.0**
13	0.000	0.0000°	**1.1**	**0.5**	**0.0**	**0.1**	**0.1**	**0.1**	**0.1**	**0.1**	**0.1**
14	0.000	0.0000°	**0.3**	**0.0**	**0.0**	**0.1**	**0.1**	**0.2**	**0.1**	**0.1**	**0.1**
15	0.000	0.0000°	**0.2**	**0.0**	**0.1**	**0.1**	**0.1**	**0.1**	**0.1**	**0.1**	**0.1**
16	0.000	0.0000°	**0.2**	**0.0**	**0.0**	**0.1**	**0.1**	**0.0**	**0.1**	**0.1**	**0.1**
17	0.000	0.0000°	**1.0**	**1.5**	**0.0**	**0.0**	**0.2**	**0.1**	**0.1**	**0.1**	**0.1**
18	0.000	0.0000°	**0.0**	**0.0**	**0.0**	**0.0**	**0.1**	**0.1**	**0.1**	**0.1**	**0.1**
19	0.000	0.0000°	**0.6**	**1.1**	**0.0**	**0.0**	**0.2**	**0.1**	**0.1**	**0.1**	**0.0**
20	0.000	0.0000°	**0.1**	**0.0**	0.0	**0.0**	**0.3**	**0.2**	**0.1**	**0.1**	**0.1**
Ave	0.0000	0.0000°	-	-	-	-	-	-	-	-	-
Var	0.0000	0.0000°	-	-	-	-	-	-	-	-	-

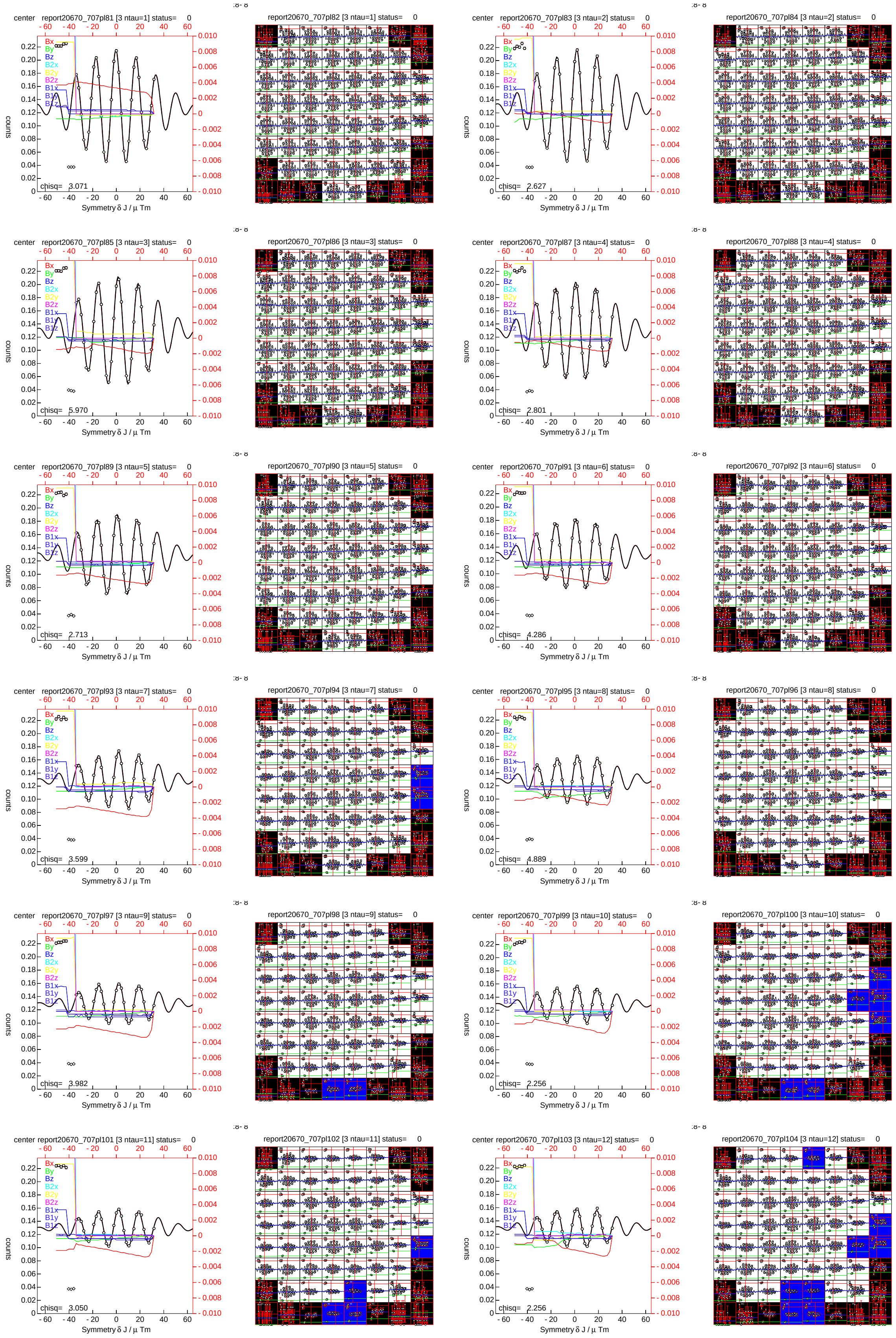

Figure 5: Center phase scan plots. Dashed red line + diamonds: scaled reference.

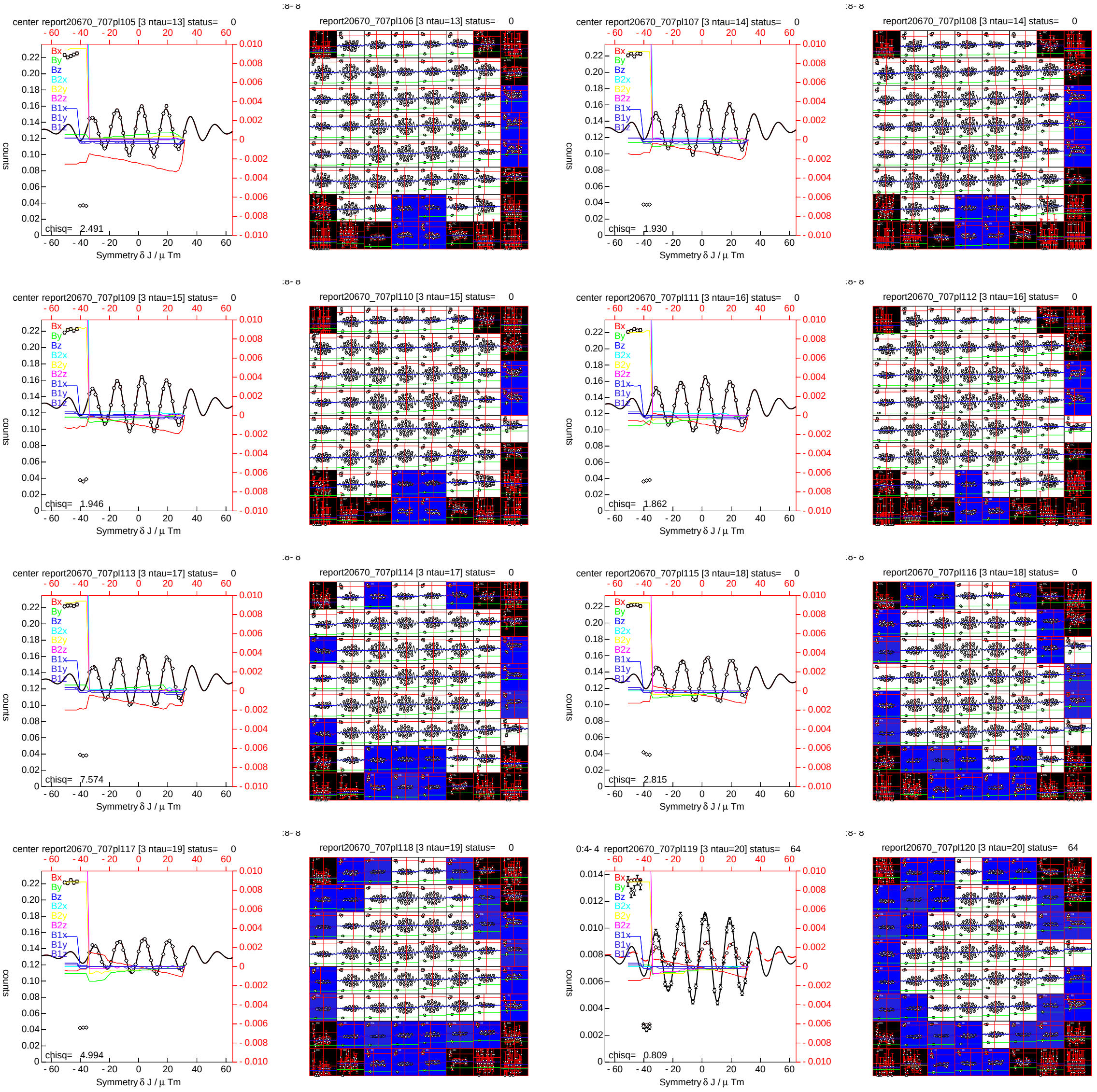

Figure 6: Continuation: Center phase scan plots. Dashed red line + diamonds: scaled reference.

20676 m20676.echo reference 18 pnts

actC4mm Sun Jun 24 21:57:58 2018

actC4mm "actC 4mm at sampos in spongebob"

actC4mm "actC 4mm at sampos in spongebob"

matching reference: 20676

matching background: –

wavelength $\lambda = 8.0000$ Å

$Q/\text{Å}^{-1} = 0.1499$ ($2\theta = 10.953$ ° $\Psi = -84.510$ °)

Up = 25.292 Down= 2.8427

Normalized echo amplitude (J_{min}, J_{max})

($\tau_{min} = 0.0948$ ns $\cdots$ $\tau_{max} = 69.725$ ns) at detector center: 0.9705 0.7035

<table>
<tr><th>#</th><th>J0/muTm</th><th>theta</th><th>nt</th><th>npic</th><th>nact</th><th>average0</th><th><average></th><th><amplitu></th><th>dj0/muTm</th><th>dja/muTm</th><th>djv/muTm</th><th>lam1/A</th><th>lam2/A</th><th>ref</th><th>pt</th><th>bgr</th><th>pt</th><th>distribution: dj-count per 1muTm</th></tr>
<tr><td>1</td><td>994.03</td><td>10.95</td><td>1</td><td>64</td><td>55</td><td>1.38382E+01</td><td>9.79105E+00</td><td>7.42554E+00</td><td>-0.4679</td><td>0.5036</td><td>1.3071</td><td>8.000</td><td>8.000</td><td>20676</td><td>1</td><td>--</td><td>--</td><td>| 0 0 0 0 0 9131212 9 0 0 0 0 0|</td></tr>
<tr><td>2</td><td>2555.51</td><td>10.95</td><td>1</td><td>64</td><td>55</td><td>1.37214E+01</td><td>9.76068E+00</td><td>7.22015E+00</td><td>2.4473</td><td>1.8164</td><td>0.6463</td><td>8.000</td><td>8.000</td><td>20676</td><td>2</td><td>--</td><td>--</td><td>| 0 0 0 0 0 0123013 0 0 0 0 0 0|</td></tr>
<tr><td>3</td><td>5167.14</td><td>10.95</td><td>1</td><td>64</td><td>55</td><td>1.38256E+01</td><td>9.78384E+00</td><td>6.94447E+00</td><td>2.4621</td><td>0.1782</td><td>1.3121</td><td>8.000</td><td>8.000</td><td>20676</td><td>3</td><td>--</td><td>--</td><td>| 0 0 0 0 0 7151212 9 0 0 0 0 0|</td></tr>
<tr><td>4</td><td>10387.85</td><td>10.95</td><td>1</td><td>64</td><td>55</td><td>1.37942E+01</td><td>9.79217E+00</td><td>6.50337E+00</td><td>2.3077</td><td>-1.9426</td><td>2.4590</td><td>8.000</td><td>8.000</td><td>20676</td><td>4</td><td>--</td><td>--</td><td>| 0 0 1 2 6 910 5 4 8 3 6 1 0 0|</td></tr>
<tr><td>5</td><td>20828.60</td><td>10.95</td><td>1</td><td>64</td><td>54</td><td>1.37969E+01</td><td>9.94216E+00</td><td>5.87891E+00</td><td>2.6502</td><td>-3.7143</td><td>3.6974</td><td>8.000</td><td>8.000</td><td>20676</td><td>5</td><td>--</td><td>--</td><td>| 2 0 3 3 9 5 7 4 1 5 5 1 3 4 2|</td></tr>
<tr><td>6</td><td>31268.85</td><td>10.95</td><td>1</td><td>64</td><td>55</td><td>1.36367E+01</td><td>9.75819E+00</td><td>5.18672E+00</td><td>3.1693</td><td>-4.5818</td><td>4.4330</td><td>8.000</td><td>8.000</td><td>20676</td><td>6</td><td>--</td><td>--</td><td>| 2 3 2 7 3 7 7 3 0 5 2 4 1 2 7|</td></tr>
<tr><td>7</td><td>52150.00</td><td>10.95</td><td>1</td><td>64</td><td>52</td><td>1.36878E+01</td><td>1.02034E+01</td><td>4.60005E+00</td><td>4.2611</td><td>-4.5496</td><td>6.2834</td><td>8.000</td><td>8.000</td><td>20676</td><td>7</td><td>--</td><td>--</td><td>| 8 4 2 2 5 3 4 0 0 7 2 4 0 2 9|</td></tr>
<tr><td>8</td><td>73030.30</td><td>10.95</td><td>1</td><td>64</td><td>55</td><td>1.37080E+01</td><td>9.75920E+00</td><td>4.03343E+00</td><td>4.6080</td><td>-5.1323</td><td>6.6503</td><td>8.000</td><td>8.000</td><td>20676</td><td>8</td><td>--</td><td>--</td><td>|10 5 0 4 5 2 3 0 0 4 2 4 1 213|</td></tr>
<tr><td>9</td><td>104351.50</td><td>10.95</td><td>1</td><td>64</td><td>53</td><td>1.36464E+01</td><td>1.00298E+01</td><td>3.76896E+00</td><td>4.3990</td><td>-5.4867</td><td>8.0578</td><td>8.000</td><td>8.000</td><td>20676</td><td>9</td><td>--</td><td>--</td><td>|14 1 3 3 4 1 2 0 0 4 2 6 0 112|</td></tr>
<tr><td>10</td><td>156553.50</td><td>10.95</td><td>1</td><td>64</td><td>49</td><td>1.38237E+01</td><td>1.06727E+01</td><td>3.76406E+00</td><td>3.9868</td><td>-6.4167</td><td>7.7692</td><td>8.000</td><td>8.000</td><td>20676</td><td>10</td><td>--</td><td>--</td><td>|13 1 3 3 3 2 1 0 0 5 1 4 1 111|</td></tr>
<tr><td>11</td><td>260957.50</td><td>10.95</td><td>1</td><td>64</td><td>44</td><td>1.39368E+01</td><td>1.09032E+01</td><td>4.14399E+00</td><td>3.1970</td><td>-6.7456</td><td>6.9156</td><td>8.000</td><td>8.000</td><td>20676</td><td>11</td><td>--</td><td>--</td><td>| 7 3 2 5 2 2 0 2 0 3 4 2 2 1 9|</td></tr>
<tr><td>12</td><td>313156.00</td><td>10.95</td><td>1</td><td>64</td><td>45</td><td>1.38407E+01</td><td>1.08383E+01</td><td>4.25468E+00</td><td>3.0945</td><td>-5.8028</td><td>6.2611</td><td>8.000</td><td>8.000</td><td>20676</td><td>12</td><td>--</td><td>--</td><td>| 5 4 2 3 4 3 0 2 2 4 3 3 1 0 9|</td></tr>
<tr><td>13</td><td>365357.50</td><td>10.95</td><td>1</td><td>64</td><td>45</td><td>1.39059E+01</td><td>1.08403E+01</td><td>4.42543E+00</td><td>3.0824</td><td>-4.7514</td><td>5.8150</td><td>8.000</td><td>8.000</td><td>20676</td><td>13</td><td>--</td><td>--</td><td>| 5 2 4 3 2 0 5 2 4 1 4 4 0 2 7|</td></tr>
<tr><td>14</td><td>417559.00</td><td>10.95</td><td>1</td><td>64</td><td>45</td><td>1.39225E+01</td><td>1.10185E+01</td><td>4.61669E+00</td><td>3.1370</td><td>-3.1325</td><td>5.7710</td><td>8.000</td><td>8.000</td><td>20676</td><td>14</td><td>--</td><td>--</td><td>| 7 1 1 4 2 2 1 4 3 5 3 3 1 1 7|</td></tr>
<tr><td>15</td><td>469760.00</td><td>10.95</td><td>1</td><td>64</td><td>46</td><td>1.40915E+01</td><td>1.07789E+01</td><td>4.61226E+00</td><td>3.0847</td><td>-2.1874</td><td>5.8995</td><td>8.000</td><td>8.000</td><td>20676</td><td>15</td><td>--</td><td>--</td><td>| 8 2 0 1 3 3 3 2 1 5 3 5 2 3 5|</td></tr>
<tr><td>16</td><td>521961.00</td><td>10.95</td><td>1</td><td>64</td><td>45</td><td>1.40553E+01</td><td>1.09926E+01</td><td>4.74909E+00</td><td>2.9539</td><td>-0.9569</td><td>5.4048</td><td>8.000</td><td>8.000</td><td>20676</td><td>16</td><td>--</td><td>--</td><td>| 7 2 1 2 1 2 3 4 3 4 2 3 4 2 5|</td></tr>
<tr><td>17</td><td>626366.50</td><td>10.95</td><td>1</td><td>64</td><td>41</td><td>1.41124E+01</td><td>1.12709E+01</td><td>4.84959E+00</td><td>2.7423</td><td>1.9035</td><td>6.0902</td><td>8.000</td><td>8.000</td><td>20676</td><td>17</td><td>--</td><td>--</td><td>| 8 1 2 2 0 1 4 1 4 4 4 3 2 0 5|</td></tr>
<tr><td>18</td><td>730769.50</td><td>10.95</td><td>1</td><td>64</td><td>33</td><td>1.41743E+01</td><td>1.23428E+01</td><td>5.27913E+00</td><td>2.6495</td><td>3.1324</td><td>5.2175</td><td>8.000</td><td>8.000</td><td>20676</td><td>18</td><td>--</td><td>--</td><td>| 4 3 1 0 3 1 2 3 2 4 3 1 2 2 2|</td></tr>
</table>

Table 6: Applied phase offsets and $10^6 \times \chi^2$ of aux. parameters (B_x, B_y,.. And Δ = difference ref-sample in 10^{-3} units (B:milliGauss).

nt	$\Delta J/\mu$Tm	$\simeq \Delta\Phi$	Bx	By	Bz	B2x	B2y	B2z	B1x	B1y	B1z
1	0.000	0.0000°	**0.2**	**0.0**	**0.0**	**0.1**	**0.1**	**0.1**	**0.0**	**0.0**	**0.1**
2	0.000	0.0000°	**0.1**	**0.0**	**0.0**	**0.0**	**0.0**	**0.1**	**0.0**	**0.1**	**0.1**
3	0.000	0.0000°	**0.1**	**0.0**	**0.0**	**0.0**	**0.0**	**0.0**	**0.1**	**0.1**	**0.1**
4	0.000	0.0000°	**0.4**	**0.0**	**2.4**	**0.1**	**0.0**	**0.3**	**0.0**	**0.2**	**0.2**
5	0.000	0.0000°	**0.4**	**0.0**	**0.1**	**0.0**	**0.0**	**0.2**	**0.0**	**0.2**	**0.1**
6	0.000	0.0000°	**0.4**	**0.0**	**0.0**	**0.0**	**0.1**	**0.1**	**0.2**	**0.0**	**0.0**
7	0.000	0.0000°	**0.5**	**0.0**	**0.1**	**0.1**	**0.0**	**0.0**	**0.1**	**0.1**	**0.0**
8	0.000	0.0000°	**0.7**	**0.0**	**0.0**	**0.0**	**0.1**	**0.1**	**0.1**	**0.0**	**0.0**
9	0.000	0.0000°	**0.7**	**0.0**	**0.0**	**0.1**	**0.1**	**0.0**	**0.1**	**0.1**	**0.0**
10	0.000	0.0000°	**0.4**	**0.1**	**0.0**	**0.1**	**0.1**	**0.0**	**0.0**	**0.2**	**0.0**
11	0.000	0.0000°	**0.3**	**0.0**	**0.0**	**0.1**	**0.1**	**0.1**	**0.0**	**0.1**	**0.1**
12	0.000	0.0000°	**0.4**	**0.0**	**0.0**	**0.1**	**0.1**	**0.0**	**0.1**	**0.1**	**0.1**
13	0.000	0.0000°	**0.3**	**0.1**	**0.0**	**0.1**	**0.1**	**0.2**	**0.1**	**0.1**	**0.1**
14	0.000	0.0000°	**0.1**	**0.0**	**0.0**	**0.1**	**0.1**	**0.0**	**0.1**	**0.1**	**0.1**
15	0.000	0.0000°	**0.2**	**0.0**	**0.0**	**0.0**	**0.1**	**0.2**	**0.1**	**0.1**	**0.1**
16	0.000	0.0000°	**0.1**	**0.0**	**0.1**	**0.1**	**0.1**	**0.1**	**0.1**	**0.1**	**0.0**
17	0.000	0.0000°	**0.1**	**0.0**	**0.0**	**0.1**	**0.1**	**0.0**	**0.1**	**0.1**	**0.1**
18	0.000	0.0000°	**0.1**	**0.1**	**0.0**	**0.0**	**0.1**	**0.1**	**0.1**	**0.1**	**0.1**
Ave	0.0000	0.0000°	-	-	-	-	-	-	-	-	-
Var	0.0000	0.0000°	-	-	-	-	-	-	-	-	-

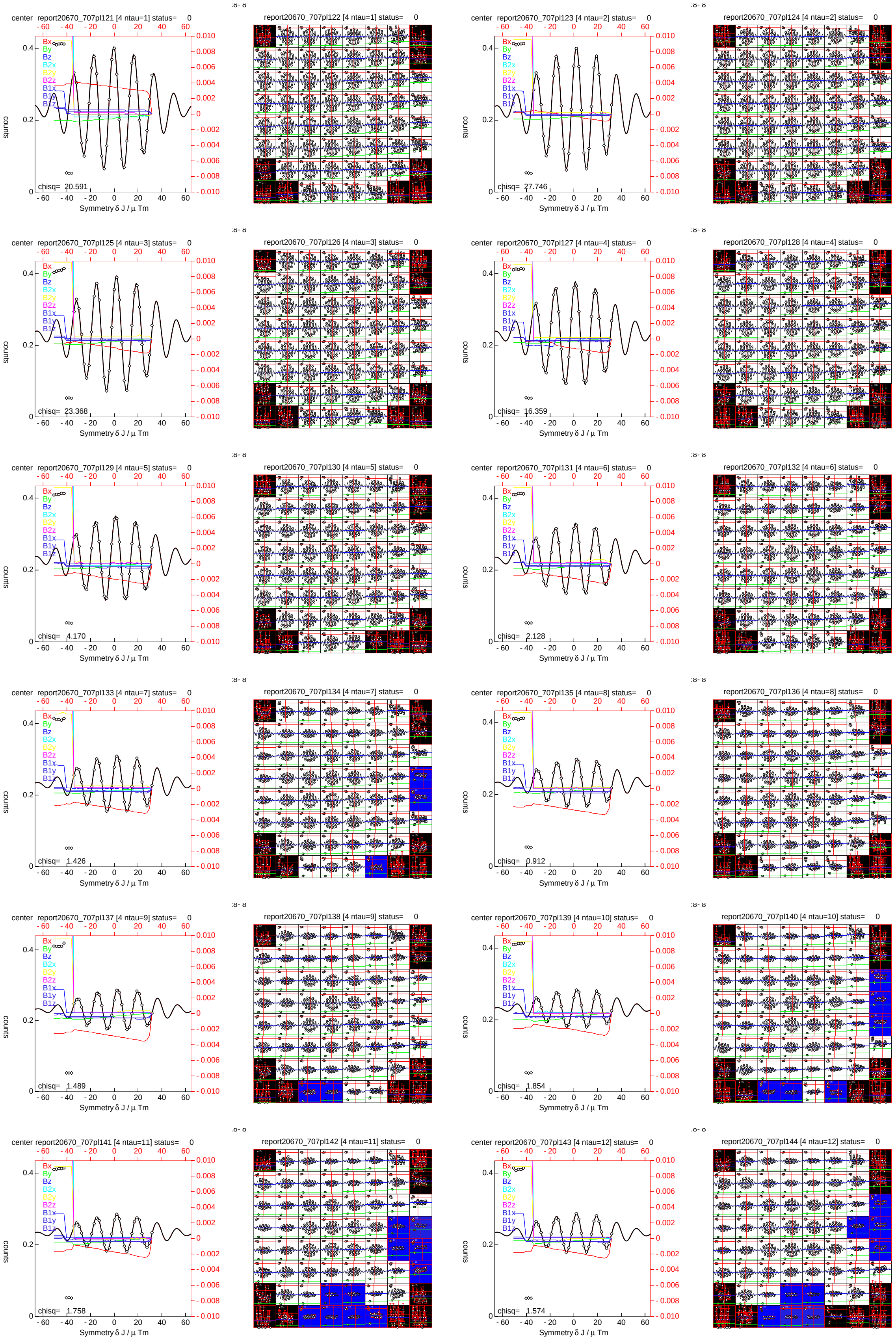

Figure 7: Center phase scan plots. Dashed red line + diamonds: scaled reference.

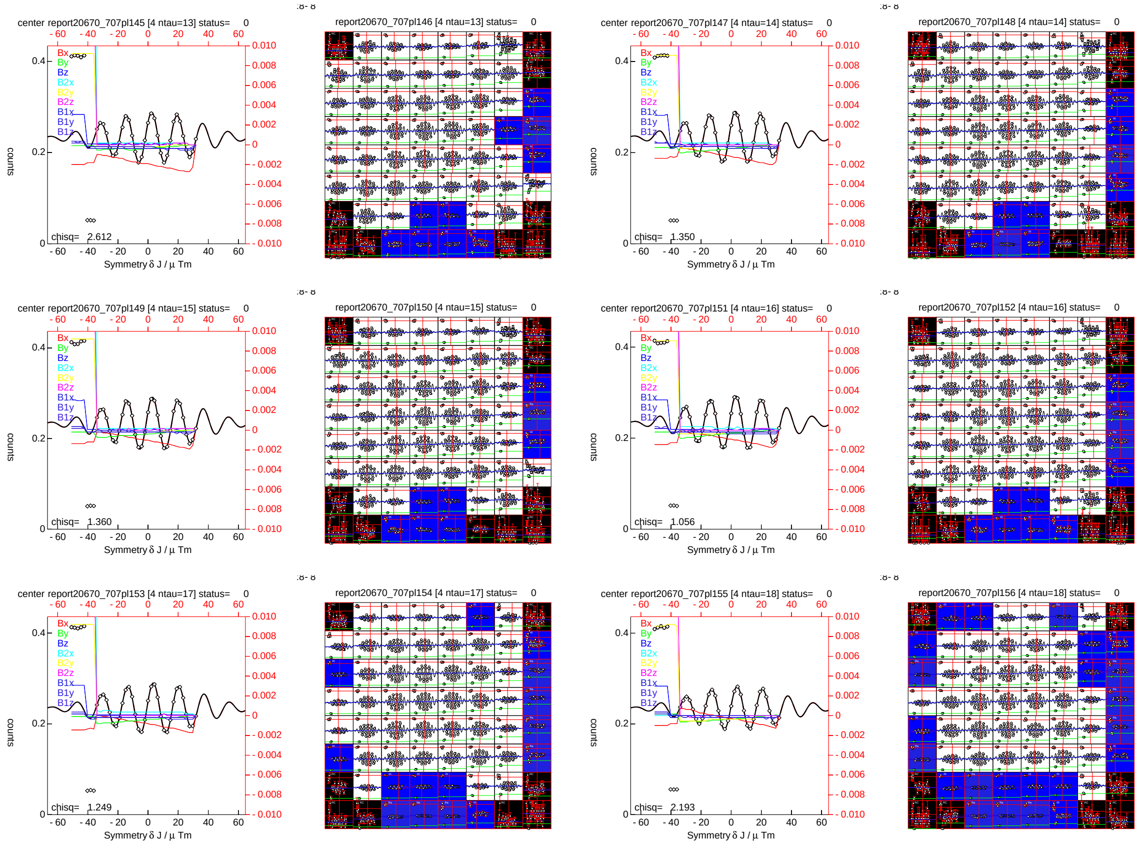

Figure 8: Continuation: Center phase scan plots. Dashed red line + diamonds: scaled reference.

20670 m20670.echo **SDS5wD2O Sun Jun 24 09:31:34 2018** SDS5wD2O "SDS with N

sample 16 pnts

SDS5wD2O "SDS with NaCl in D2O 0.05w in refpos, room temp"

SDS5wD2O "SDS with NaCl in D2O 0.05w in refpos, room temp"

matching reference: 20666
matching background: –
wavelength $\lambda = 8.0000$ Å

$Q/\text{Å}^{-1} = 0.0505$ ($2\theta = 3.6859$ ° $\Psi = -88.185$ °)

Up = 51.525 Down= 5.6819

Normalized echo amplitude (J_{min}, J_{max})

($\tau_{min} = 0.0947$ ns $\cdots$ $\tau_{max} = 69.724$ ns) at detector center: 0.9623 0.1925

<table>
<tr><th>#</th><th>J0/muTm</th><th>theta</th><th>nt</th><th>npic</th><th>nact</th><th>average0</th><th><average></th><th><amplitu></th><th>dj0/muTm</th><th>dja/muTm</th><th>djv/muTm</th><th>lam1/A</th><th>lam2/A</th><th>ref</th><th>pt</th><th>bgr</th><th>pt</th><th>distribution: dj-count per 1muTm</th></tr>
<tr><td>1</td><td>992.49</td><td>3.69</td><td>1</td><td>64</td><td>55</td><td>2.80896E+01</td><td>2.57611E+01</td><td>1.80748E+01</td><td>-0.4717</td><td>0.0409</td><td>1.2311</td><td>8.000</td><td>8.000</td><td>20666</td><td>1</td><td>--</td><td>--</td><td>| 0 0 0 0 0 9141113 8 0 0 0 0 0|</td></tr>
<tr><td>2</td><td>2555.64</td><td>3.69</td><td>1</td><td>64</td><td>55</td><td>2.83162E+01</td><td>2.58594E+01</td><td>1.74217E+01</td><td>1.7685</td><td>0.6896</td><td>0.9103</td><td>8.000</td><td>8.000</td><td>20666</td><td>2</td><td>--</td><td>--</td><td>| 0 0 0 0 0 4122018 1 0 0 0 0 0|</td></tr>
<tr><td>3</td><td>5167.15</td><td>3.69</td><td>1</td><td>64</td><td>55</td><td>2.82693E+01</td><td>2.58702E+01</td><td>1.67510E+01</td><td>1.6465</td><td>-1.0726</td><td>1.5604</td><td>8.000</td><td>8.000</td><td>20666</td><td>3</td><td>--</td><td>--</td><td>| 0 0 0 0 1101311 7 9 4 0 0 0 0|</td></tr>
<tr><td>4</td><td>10387.80</td><td>3.69</td><td>1</td><td>64</td><td>55</td><td>2.85126E+01</td><td>2.60216E+01</td><td>1.56997E+01</td><td>1.7843</td><td>-2.8603</td><td>2.5767</td><td>8.000</td><td>8.000</td><td>20666</td><td>4</td><td>--</td><td>--</td><td>| 0 0 1 2 7 810 4 6 5 5 5 2 0 0|</td></tr>
<tr><td>5</td><td>20828.60</td><td>3.69</td><td>1</td><td>64</td><td>55</td><td>2.85164E+01</td><td>2.60610E+01</td><td>1.36302E+01</td><td>2.0536</td><td>-4.6536</td><td>3.7086</td><td>8.000</td><td>8.000</td><td>20666</td><td>5</td><td>--</td><td>--</td><td>| 1 2 1 7 210 6 4 3 2 6 2 2 4 3|</td></tr>
<tr><td>6</td><td>31268.85</td><td>3.69</td><td>1</td><td>64</td><td>55</td><td>2.85018E+01</td><td>2.60676E+01</td><td>1.20423E+01</td><td>2.7065</td><td>-5.1806</td><td>4.3748</td><td>8.000</td><td>8.000</td><td>20666</td><td>6</td><td>--</td><td>--</td><td>| 3 2 5 2 5 5 8 3 2 3 5 2 2 2 6|</td></tr>
<tr><td>7</td><td>52149.95</td><td>3.69</td><td>1</td><td>64</td><td>53</td><td>2.86085E+01</td><td>2.62573E+01</td><td>1.00505E+01</td><td>3.5965</td><td>-5.4928</td><td>5.5050</td><td>8.000</td><td>8.000</td><td>20666</td><td>7</td><td>--</td><td>--</td><td>| 8 3 1 4 5 1 4 2 2 2 4 6 1 2 8|</td></tr>
<tr><td>8</td><td>73030.30</td><td>3.69</td><td>1</td><td>64</td><td>55</td><td>2.83144E+01</td><td>2.60702E+01</td><td>8.62696E+00</td><td>4.0715</td><td>-5.5393</td><td>6.2483</td><td>8.000</td><td>8.000</td><td>20666</td><td>8</td><td>--</td><td>--</td><td>|12 1 3 3 5 3 1 1 2 2 2 5 1 113|</td></tr>
<tr><td>9</td><td>104351.50</td><td>3.69</td><td>1</td><td>64</td><td>51</td><td>2.86520E+01</td><td>2.76164E+01</td><td>7.82476E+00</td><td>3.8972</td><td>-7.3585</td><td>6.6768</td><td>8.000</td><td>8.000</td><td>20666</td><td>9</td><td>--</td><td>--</td><td>|11 2 3 2 2 5 3 2 0 1 3 2 3 210|</td></tr>
<tr><td>10</td><td>156553.50</td><td>3.69</td><td>1</td><td>64</td><td>48</td><td>2.86799E+01</td><td>2.81396E+01</td><td>6.85538E+00</td><td>3.4961</td><td>-7.3768</td><td>6.5432</td><td>8.000</td><td>8.000</td><td>20666</td><td>10</td><td>--</td><td>--</td><td>| 7 5 3 3 3 2 3 0 1 2 3 3 3 1 9|</td></tr>
<tr><td>11</td><td>260957.00</td><td>3.69</td><td>1</td><td>64</td><td>49</td><td>2.87695E+01</td><td>2.79268E+01</td><td>5.66141E+00</td><td>3.1313</td><td>-7.1999</td><td>6.5453</td><td>8.000</td><td>8.000</td><td>20666</td><td>11</td><td>--</td><td>--</td><td>| 6 2 3 8 1 5 0 2 1 0 6 3 2 1 9|</td></tr>
<tr><td>12</td><td>313156.00</td><td>3.69</td><td>1</td><td>64</td><td>49</td><td>2.87502E+01</td><td>2.79075E+01</td><td>5.33536E+00</td><td>2.7523</td><td>-5.8662</td><td>5.6910</td><td>8.000</td><td>8.000</td><td>20666</td><td>12</td><td>--</td><td>--</td><td>| 5 3 3 4 6 0 2 2 1 5 7 1 0 010|</td></tr>
<tr><td>13</td><td>417560.00</td><td>3.69</td><td>1</td><td>64</td><td>47</td><td>2.85612E+01</td><td>2.75106E+01</td><td>4.74368E+00</td><td>2.7866</td><td>-3.3857</td><td>5.3218</td><td>8.000</td><td>8.000</td><td>20666</td><td>14</td><td>--</td><td>--</td><td>| 5 1 4 3 1 2 2 4 5 4 2 4 2 2 6|</td></tr>
<tr><td>14</td><td>521962.00</td><td>3.69</td><td>1</td><td>64</td><td>46</td><td>2.92092E+01</td><td>2.85397E+01</td><td>4.11802E+00</td><td>2.3719</td><td>-1.5684</td><td>5.4557</td><td>8.000</td><td>8.000</td><td>20666</td><td>16</td><td>--</td><td>--</td><td>| 7 2 2 3 1 2 1 1 5 6 1 5 4 3 3|</td></tr>
<tr><td>15</td><td>626364.00</td><td>3.69</td><td>1</td><td>64</td><td>43</td><td>2.89366E+01</td><td>2.92106E+01</td><td>3.43893E+00</td><td>2.3007</td><td>1.5241</td><td>6.6004</td><td>8.000</td><td>8.000</td><td>20666</td><td>17</td><td>--</td><td>--</td><td>| 7 1 3 2 4 1 1 3 4 3 3 2 2 1 6|</td></tr>
<tr><td>16</td><td>730765.50</td><td>3.69</td><td>1</td><td>64</td><td>34</td><td>2.91007E+01</td><td>3.17962E+01</td><td>3.15239E+00</td><td>1.8778</td><td>1.8157</td><td>5.1992</td><td>8.000</td><td>8.000</td><td>20666</td><td>18</td><td>--</td><td>--</td><td>| 4 2 1 1 3 2 0 3 1 8 2 1 0 2 4|</td></tr>
</table>

Table 7: Applied phase offsets and $10^6 \times \chi^2$ of aux. parameters (B_x, B_y,.. And Δ = difference ref-sample in 10^{-3} units (B:milliGauss).

nt	$\Delta J/\mu$Tm	$\simeq \Delta\Phi$	Bx	By	Bz	B2x	B2y	B2z	B1x	B1y	B1z
1	−0.366	−7.7645°	**0.4Δ 1.4**	**0.0Δ 0.2**	**0.0Δ 0.8**	**0.0Δ 0.4**	**0.1Δ -0.8**	**0.0Δ -0.2**	**0.0Δ 0.4**	0.0Δ 0.3	**0.0**
2	−0.273	−5.8023°	**0.0Δ 1.8**	**0.0Δ 0.5**	**0.0Δ 1.0**	**0.0Δ 0.4**	**0.0Δ -0.6**	**0.0Δ -0.2**	**0.0Δ 0.5**	**0.0Δ 0.5**	**0.1**
3	−0.391	−8.3065°	**0.1Δ 2.0**	**0.0Δ 0.5**	**0.0Δ 0.9**	**0.1Δ 0.5**	**0.0Δ -0.7**	**0.0Δ -0.2**	**0.1Δ 0.5**	**0.0Δ 0.5**	**0.0**
4	−0.223	−4.7352°	**0.5Δ 2.2**	**0.0Δ 0.5**	**0.0Δ 0.9**	**0.0Δ 0.5**	**0.1Δ -0.8**	**0.0Δ -0.4**	**0.0Δ 0.5**	**0.0Δ 0.5**	**0.0**
5	−0.310	−6.5877°	**0.7Δ 2.5**	**0.0Δ 0.6**	**0.0Δ 0.9**	**0.1Δ 0.7**	**0.0Δ -1.1**	**0.0Δ -0.4**	**0.0Δ 0.6**	**0.0Δ 0.6**	**0.1**
6	−0.149	−3.1713°	**0.7Δ 2.4**	**0.0Δ 0.6**	**0.0Δ 0.9**	**0.0Δ 0.7**	**0.0Δ -1.1**	0.0Δ -0.4	**0.1Δ 0.6**	**0.0Δ 0.6**	**0.0**
7	−0.255	−5.4237°	**0.5Δ 2.8**	**0.4Δ -0.0**	**0.0Δ 0.9**	**0.0Δ 0.9**	**0.1Δ -1.3**	**0.1Δ -0.5**	**0.1Δ 0.8**	**0.1Δ 0.6**	**0.1**
8	−0.128	−2.7078°	**3.3Δ 2.4**	**0.5Δ 0.3**	**0.0Δ 0.8**	**0.0Δ 0.9**	**0.2Δ -1.2**	**0.0Δ -0.4**	**0.1Δ 0.5**	**0.1Δ 0.5**	**0.1**
9	0.112	2.3771°	**1.3Δ 2.5**	**0.0Δ 0.1**	**0.0Δ 0.9**	**0.0Δ 0.8**	**0.1Δ -1.2**	**0.0Δ -0.4**	**0.0Δ 0.5**	**0.1Δ 0.5**	**0.1**
10	−0.129	−2.7477°	**0.9Δ 2.2**	**0.0Δ -0.1**	**0.0Δ 0.8**	**0.0Δ 0.8**	**0.1Δ -1.2**	**0.0Δ -0.4**	**0.1Δ 0.4**	**0.0Δ 0.5**	**0.1**
11	0.201	4.2708°	**0.8Δ 1.7**	**0.0Δ 0.2**	**0.0Δ 0.9**	**0.0Δ 0.7**	**0.0Δ -1.0**	0.0Δ -0.4	**0.0Δ 0.4**	**0.1Δ 0.4**	**0.1**
12	−0.180	−3.8130°	**0.6Δ 1.2**	**0.1Δ 0.4**	**0.0Δ 0.9**	**0.1Δ 0.6**	**0.0Δ -1.0**	**0.0Δ -0.3**	**0.1Δ 0.4**	**0.1Δ 0.3**	**0.0**
13	−0.038	−0.8151°	**0.5Δ 1.6**	**0.4Δ -0.7**	**0.0Δ 0.9**	**0.1Δ 0.7**	**0.2Δ -1.3**	**0.0Δ -0.2**	**0.0Δ 0.3**	**0.1Δ 0.3**	**0.1**
14	−0.354	−7.5147°	**0.4Δ 0.5**	**0.0Δ -0.0**	**0.0Δ 1.0**	**0.0Δ 0.5**	**0.0Δ -1.0**	**0.0Δ -0.2**	**0.0Δ 0.2**	**0.0Δ 0.3**	**0.1**
15	−0.278	−5.9103°	**0.1Δ -0.1**	**0.0Δ -0.1**	**0.0Δ 1.0**	**0.0Δ 0.3**	**0.0Δ -0.9**	**0.0Δ -0.2**	**0.0Δ 0.1**	**0.0Δ 0.1**	**0.0**
16	−0.613	−13.005°	**0.1Δ -0.9**	**0.0Δ 0.3**	**0.0Δ 1.0**	**0.0Δ 0.0**	**0.0Δ -0.8**	**0.0Δ -0.2**	**0.1Δ 0.1**	**0.0Δ 0.1**	**0.0**
Ave	−0.2109	−4.4786°	-	-	-	-	-	-	-	-	-
Var	0.1905	4.0453°	-	-	-	-	-	-	-	-	-

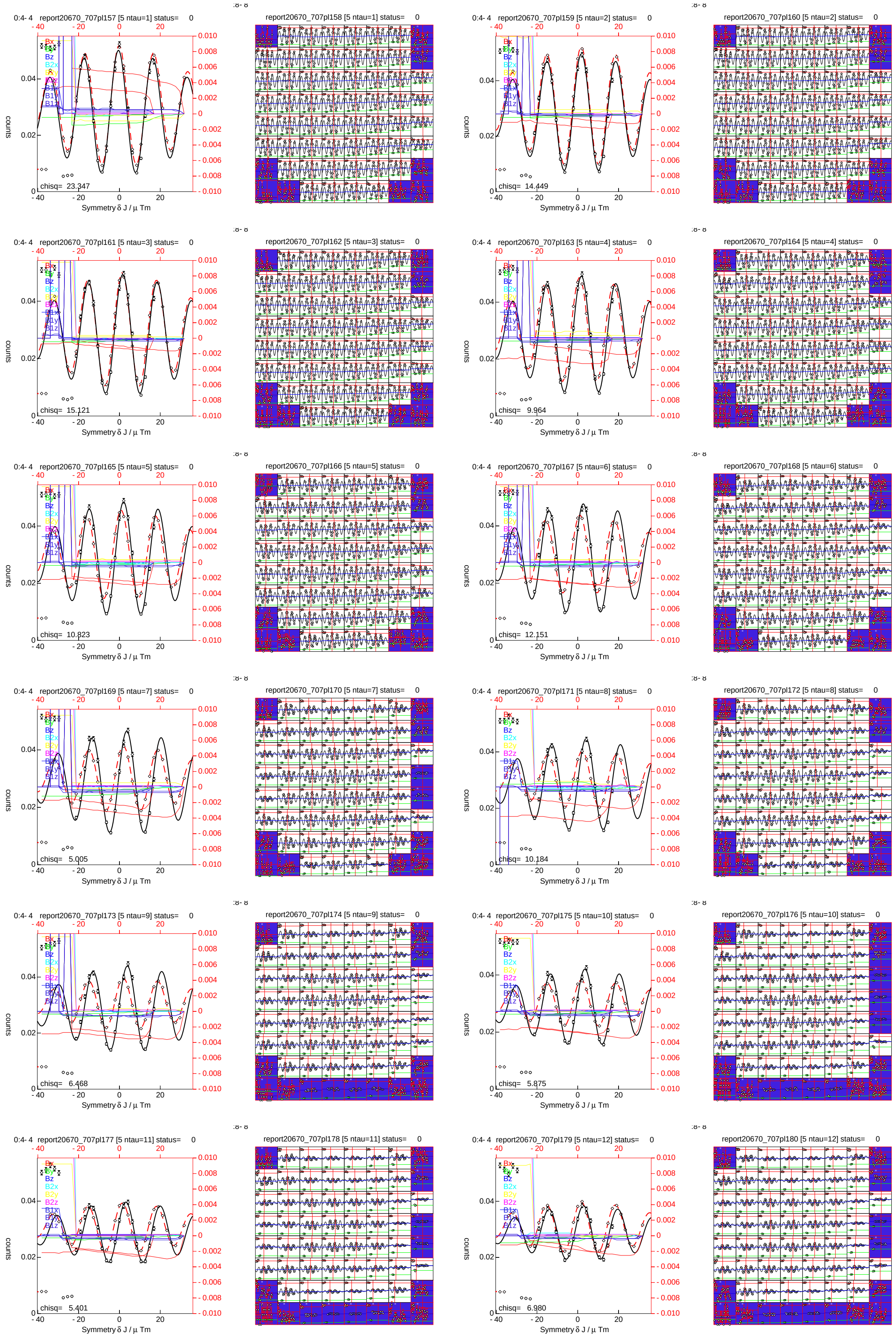

Figure 9: Center phase scan plots. Dashed red line + diamonds: scaled reference.

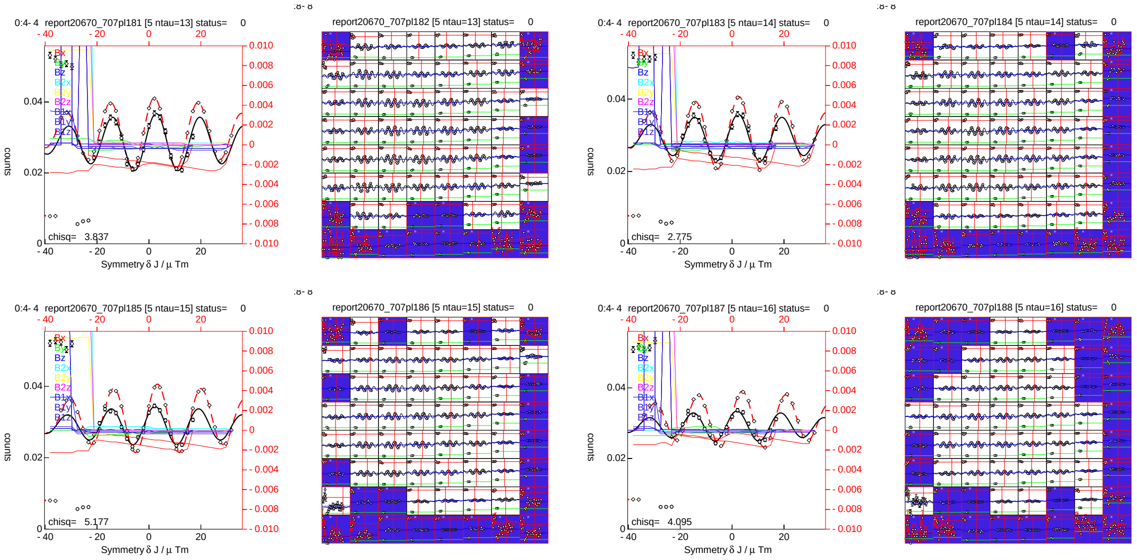

Figure 10: Continuation: Center phase scan plots. Dashed red line + diamonds: scaled reference.

20672 m20672.echo **SDS5wD2O Sun Jun 24 13:07:03 2018** SDS5wD2O "SDS with N

sample 4 pnts

SDS5wD2O "SDS with NaCl in D2O 0.05w in refpos, room temp" SDS5wD2O "SDS with NaCl in D2O 0.05w in refpos, room temp"

matching reference: 20668
matching background: –
wavelength $\lambda = 8.0000$ Å

$Q/\text{Å}^{-1} = 0.0703$ ($2\theta = 5.1279$ ° $\Psi = -87.440$ °)

Up = 85.661 Down= 9.0535

Normalized echo amplitude (J_{min}, J_{max})

($\tau_{min} = 0.0947$ ns $\cdots$ $\tau_{max} = 0.9911$ ns) at detector center: 0.9710 0.9132

```
 #      J0/muTm   theta  nt npic nact      average0    <average>    <amplitu> dj0/muTm dja/muTm djv/muTm    lam1/A    lam2/A     ref  pt      bgr pt    distribution: dj-count per 1muTm
 1       992.78    5.13   1   64   53  4.64187E+01  3.53414E+01  2.57136E+01  -0.2415   0.3650   1.2488    8.000     8.000   20668   1      -- --    | 0 0 0 0 0 9131111 9 0 0 0 0 0|
 2      2555.63    5.13   1   64   53  4.70485E+01  3.54392E+01  2.44720E+01   1.6433   0.6948   0.8146    8.000     8.000   20668   2      -- --    | 0 0 0 0 0 3122117 0 0 0 0 0 0|
 3      5167.16    5.13   1   64   53  4.73313E+01  3.57198E+01  2.37779E+01   1.8602  -0.6656   1.4311    8.000     8.000   20668   3      -- --    | 0 0 0 0 0 91410 9 9 2 0 0 0 0|
 4     10387.80    5.13   1   64   53  4.68750E+01  3.57892E+01  2.21164E+01   1.7058  -2.7025   2.4564    8.000     8.000   20668   4      -- --    | 0 0 1 1 6 910 5 5 7 2 6 1 0 0|
```

Table 8: Applied phase offsets and $10^6 \times \chi^2$ of aux. parameters (B_x, B_y,.. And Δ = difference ref-sample in 10^{-3} units (B:milliGauss).

nt	$\Delta J/\mu$Tm	$\simeq \Delta\Phi$	Bx	By	Bz	B2x	B2y	B2z	B1x	B1y	B1z
1	0.091	1.9350°	1.4Δ 0.6	0.5Δ -0.8	0.0Δ 0.7	0.0Δ -0.0	0.1Δ -0.1	0.0	0.0Δ 0.0	0.0Δ 0.1	0.0
2	−0.442	−9.3860°	0.1Δ -0.4	0.0Δ 0.6	0.0Δ 0.8	0.0Δ -0.3	0.1Δ 0.5	0.0Δ 0.0	0.1Δ 0.2	0.0Δ 0.1	0.0
3	−0.064	−1.3514°	0.1Δ 0.2	0.0Δ -0.1	0.0Δ 0.8	0.0Δ -0.2	0.0Δ 0.2	0.0Δ 0.2	0.0Δ 0.2	0.1Δ 0.1	0.0
4	−0.144	−3.0574°	0.3Δ 0.4	0.0Δ -0.1	0.5Δ 0.8	0.0Δ 0.0	0.0	0.1Δ -0.0	0.1Δ 0.2	0.1Δ 0.1	0.0
Ave	−0.1396	−2.9650°	-	-	-	-	-	-	-	-	-
Var	0.1940	4.1186°	-	-	-	-	-	-	-	-	-

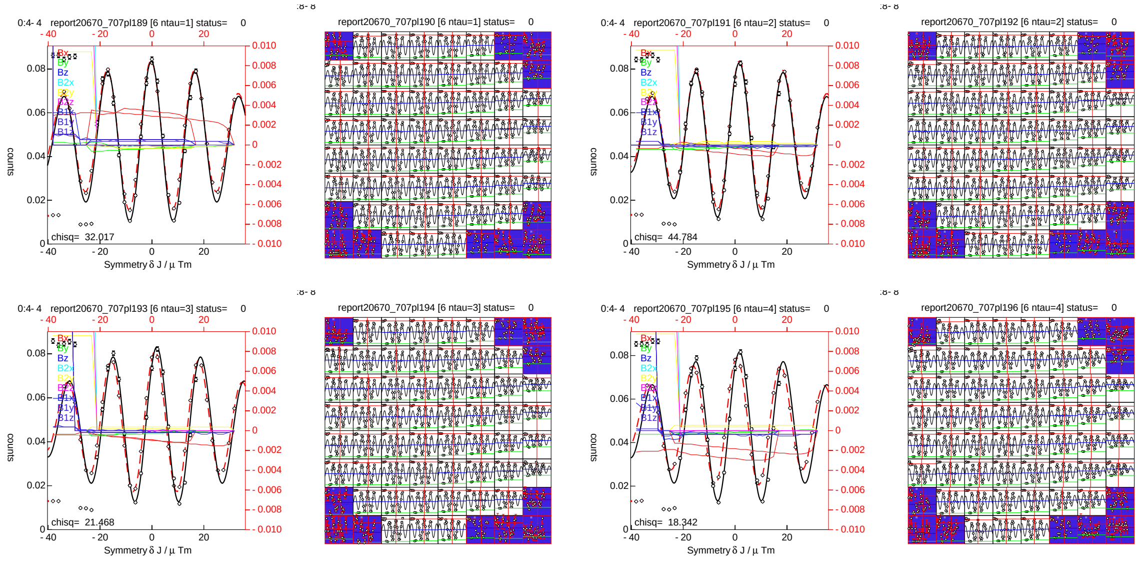

Figure 11: Center phase scan plots. Dashed red line + diamonds: scaled reference.

20673 m20673.echo **SDS5wD2O Sun Jun 24 13:50:38 2018** SDS5wD2O "SDS with N

sample 16 pnts

SDS5wD2O "SDS with NaCl in D2O 0.05w in refpos, room temp"

SDS5wD2O "SDS with NaCl in D2O 0.05w in refpos, room temp"

matching reference: 20668
matching background: –
wavelength $\lambda = 8.0000$ Å

$Q/\text{Å}^{-1} = 0.0703$ ($2\theta = 5.1279$ ° $\Psi = -87.440$ °)

Up = 84.543 Down= 8.9789

Normalized echo amplitude (J_{min}, J_{max})

($\tau_{min} = 0.0947$ ns $\cdots$ $\tau_{max} = 69.725$ ns) at detector center: 0.9724 0.1296

#	J0/muTm	theta	nt	npic	nact	average0	<average>	<amplitu>	dj0/muTm	dja/muTm	djv/muTm	lam1/A	lam2/A	ref	pt	bgr	pt	distribution: dj-count per 1muTm
1	992.78	5.13	1	64	53	4.64408E+01	3.54838E+01	2.55422E+01	-0.2488	0.3577	1.2488	8.000	8.000	20668	1	--	--	| 0 0 0 0 0 9131111 9 0 0 0 0 0|
2	2555.63	5.13	1	64	53	4.66659E+01	3.55006E+01	2.44815E+01	1.7120	0.7635	0.8146	8.000	8.000	20668	2	--	--	| 0 0 0 0 0 3122117 0 0 0 0 0 0|
3	5167.16	5.13	1	64	53	4.69963E+01	3.56547E+01	2.37290E+01	1.8990	-0.6268	1.4311	8.000	8.000	20668	3	--	--	| 0 0 0 0 0 91410 9 9 2 0 0 0 0|
4	10387.80	5.13	1	64	53	4.73788E+01	3.58689E+01	2.21500E+01	1.7667	-2.6416	2.4564	8.000	8.000	20668	4	--	--	| 0 0 1 1 6 910 5 5 7 2 6 1 0 0|
5	20828.60	5.13	1	64	53	4.74861E+01	3.59895E+01	1.93206E+01	2.2487	-4.2118	3.6218	8.000	8.000	20668	5	--	--	| 1 1 2 6 4 9 6 3 3 4 3 2 3 4 2|
6	31268.85	5.13	1	64	53	4.71933E+01	3.59907E+01	1.70136E+01	2.5499	-5.0912	4.3291	8.000	8.000	20668	6	--	--	| 2 2 5 3 4 5 7 4 1 3 5 2 2 2 6|
7	52149.95	5.13	1	64	52	4.75725E+01	3.63402E+01	1.39391E+01	3.7614	-5.0571	5.5841	8.000	8.000	20668	7	--	--	| 8 3 1 3 5 4 1 2 2 3 7 2 1 1 9|
8	73030.35	5.13	1	64	53	4.75236E+01	3.60579E+01	1.19990E+01	3.8461	-5.4072	6.5089	8.000	8.000	20668	8	--	--	|11 1 3 5 3 3 1 0 2 3 3 3 0 213|
9	104351.50	5.13	1	64	50	4.76617E+01	3.75539E+01	1.05181E+01	3.8932	-6.8951	6.6429	8.000	8.000	20668	9	--	--	|10 2 3 3 4 3 2 1 0 2 3 3 3 011|
10	156553.50	5.13	1	64	48	4.75506E+01	3.84867E+01	8.82869E+00	3.4293	-6.4170	7.3972	8.000	8.000	20668	10	--	--	|12 2 3 2 2 3 0 0 2 4 2 3 0 112|
11	260957.00	5.13	1	64	46	4.78091E+01	3.78408E+01	7.01052E+00	3.1473	-6.6488	7.1142	8.000	8.000	20668	11	--	--	| 7 3 3 4 3 1 2 1 0 3 4 4 1 010|
12	313156.00	5.13	1	64	46	4.80181E+01	3.81355E+01	6.33053E+00	2.7492	-6.5095	6.4299	8.000	8.000	20668	12	--	--	| 5 2 2 5 5 1 2 2 1 1 6 4 1 0 9|
13	417560.00	5.13	1	64	46	4.79155E+01	3.78575E+01	5.23650E+00	2.6776	-3.6194	5.9673	8.000	8.000	20668	14	--	--	| 5 4 3 1 1 3 2 3 5 3 4 2 2 2 6|
14	521962.00	5.13	1	64	44	4.83860E+01	3.90896E+01	4.24596E+00	2.2776	-1.2283	5.4906	8.000	8.000	20668	16	--	--	| 6 3 1 2 2 1 1 4 6 3 1 6 1 3 4|
15	626364.00	5.13	1	64	40	4.85500E+01	4.03624E+01	3.54736E+00	2.3212	0.9397	5.7966	8.000	8.000	20668	17	--	--	| 6 0 5 2 2 1 2 1 5 3 3 3 2 2 3|
16	730766.50	5.13	1	64	34	4.85518E+01	4.37050E+01	2.87154E+00	1.9113	2.5902	5.0262	8.000	8.000	20668	18	--	--	| 4 1 1 3 2 1 2 2 7 3 2 0 2 1 3|

Table 9: Applied phase offsets and $10^6 \times \chi^2$ of aux. parameters (B_x, B_y,.. And Δ = difference ref-sample in 10^{-3} units (B:milliGauss).

nt	$\Delta J/\mu$Tm	$\simeq \Delta\Phi$	Bx	By	Bz	B2x	B2y	B2z	B1x	B1y	B1z
1	0.084	1.7794°	**2.1Δ -0.1**	**0.5Δ -0.6**	**0.0Δ -0.2**	**0.0Δ -0.4**	**0.0Δ 0.4**	**0.0Δ -0.2**	**0.0Δ -0.1**	**0.0Δ -0.1**	**0.1**
2	−0.373	−7.9275°	**0.1Δ -1.0**	**0.0Δ 0.7**	**0.0Δ -0.1**	**0.0Δ -0.7**	**0.0Δ 0.9**	**0.0Δ -0.2**	**0.1Δ -0.0**	**0.0Δ -0.1**	**0.0**
3	−0.025	−0.5266°	**0.1Δ -0.4**	**0.0Δ 0.0**	**0.0Δ -0.1**	**0.1Δ -0.5**	**0.1Δ 0.6**	**0.0Δ -0.2**	**0.0Δ 0.0**	**0.1Δ -0.1**	**0.0**
4	−0.083	−1.7630°	**0.4Δ -0.2**	**0.0Δ -0.0**	**0.0Δ -0.1**	**0.0Δ -0.4**	**0.0Δ 0.5**	**0.0Δ -0.2**	**0.1Δ -0.1**	**0.0Δ -0.1**	**0.0**
5	0.082	1.7373°	**0.5Δ -0.0**	**0.0Δ 0.0**	**0.0Δ -0.1**	**0.1Δ -0.2**	**0.0Δ 0.4**	**0.0Δ -0.2**	**0.0Δ 0.0**	**0.1Δ -0.0**	**0.0**
6	−0.112	−2.3867°	**0.7Δ -0.2**	**0.0Δ 0.0**	**0.0Δ -0.2**	**0.0Δ -0.2**	**0.0Δ 0.5**	**0.0Δ -0.3**	**0.0Δ -0.1**	**0.0Δ -0.0**	**0.1**
7	0.042	0.9017°	**0.9Δ -0.2**	**0.0Δ 0.0**	**0.0Δ -0.2**	**0.0Δ -0.6**	**0.1Δ 0.7**	**0.0Δ -0.2**	**0.0Δ 0.0**	**0.1Δ -0.1**	**0.0**
8	−0.233	−4.9413°	**1.0Δ -0.6**	**0.0Δ 0.3**	**1.5Δ 0.5**	**0.0Δ -0.3**	**0.0Δ 0.6**	**0.5Δ -0.0**	**0.1Δ 0.0**	**0.1Δ 0.0**	**0.1**
9	0.028	0.6017°	**1.1Δ -0.1**	**0.0Δ -0.2**	**0.0Δ 0.7**	**0.0Δ 0.0**	**0.0Δ 0.4**	**0.1Δ 0.0**	**0.0Δ 0.0**	**0.0Δ 0.1**	**0.1**
10	−0.141	−2.9860°	**1.1Δ 0.0**	**0.0Δ -0.3**	**0.0Δ 0.7**	**0.0Δ 0.2**	**0.0Δ 0.0**	**0.0Δ -0.1**	**0.1Δ 0.0**	**0.0Δ 0.1**	**0.0**
11	0.011	0.2398°	**0.7Δ 0.0**	**0.0Δ -0.3**	**0.0Δ 0.7**	**0.0Δ 0.2**	**0.0Δ -0.1**	**0.0Δ -0.2**	**0.0Δ 0.1**	**0.1Δ 0.0**	**0.0**
12	−0.187	−3.9713°	**0.7Δ -0.2**	**0.0Δ -0.3**	**0.0Δ 0.7**	**0.1Δ 0.1**	**0.0Δ 0.1**	**0.0Δ -0.2**	**0.1Δ 0.0**	**0.1Δ 0.0**	**0.0**
13	−0.074	−1.5720°	**0.4Δ -0.4**	**0.0Δ -0.2**	**0.0Δ 0.7**	**0.1Δ -0.1**	**0.0Δ 0.1**	**0.0Δ -0.1**	**0.1Δ 0.0**	**0.0Δ 0.0**	**0.0**
14	−0.391	−8.2958°	**0.2Δ -0.3**	**0.0Δ -0.2**	**0.0Δ 0.7**	**0.1Δ 0.1**	**0.0Δ -0.2**	**0.1Δ -0.2**	**0.1Δ 0.0**	**0.0Δ 0.0**	**0.1**
15	−0.320	−6.7881°	**0.1Δ -0.9**	**0.0Δ 0.3**	**0.0Δ 0.7**	**0.0Δ -0.2**	**0.1Δ -0.2**	**0.0Δ -0.2**	**0.0Δ 0.0**	**0.0Δ 0.0**	**0.0**
16	−0.505	−10.722°	**0.0Δ -0.7**	**0.0Δ 0.0**	**0.0Δ 0.7**	**0.0Δ 0.0**	**0.0Δ -0.2**	**0.0Δ -0.0**	**0.0Δ 0.0**	**0.0Δ -0.0**	**0.0**
Ave	−0.1372	−2.9138°	-	-	-	-	-	-	-	-	-
Var	0.1766	3.7494°	-	-	-	-	-	-	-	-	-

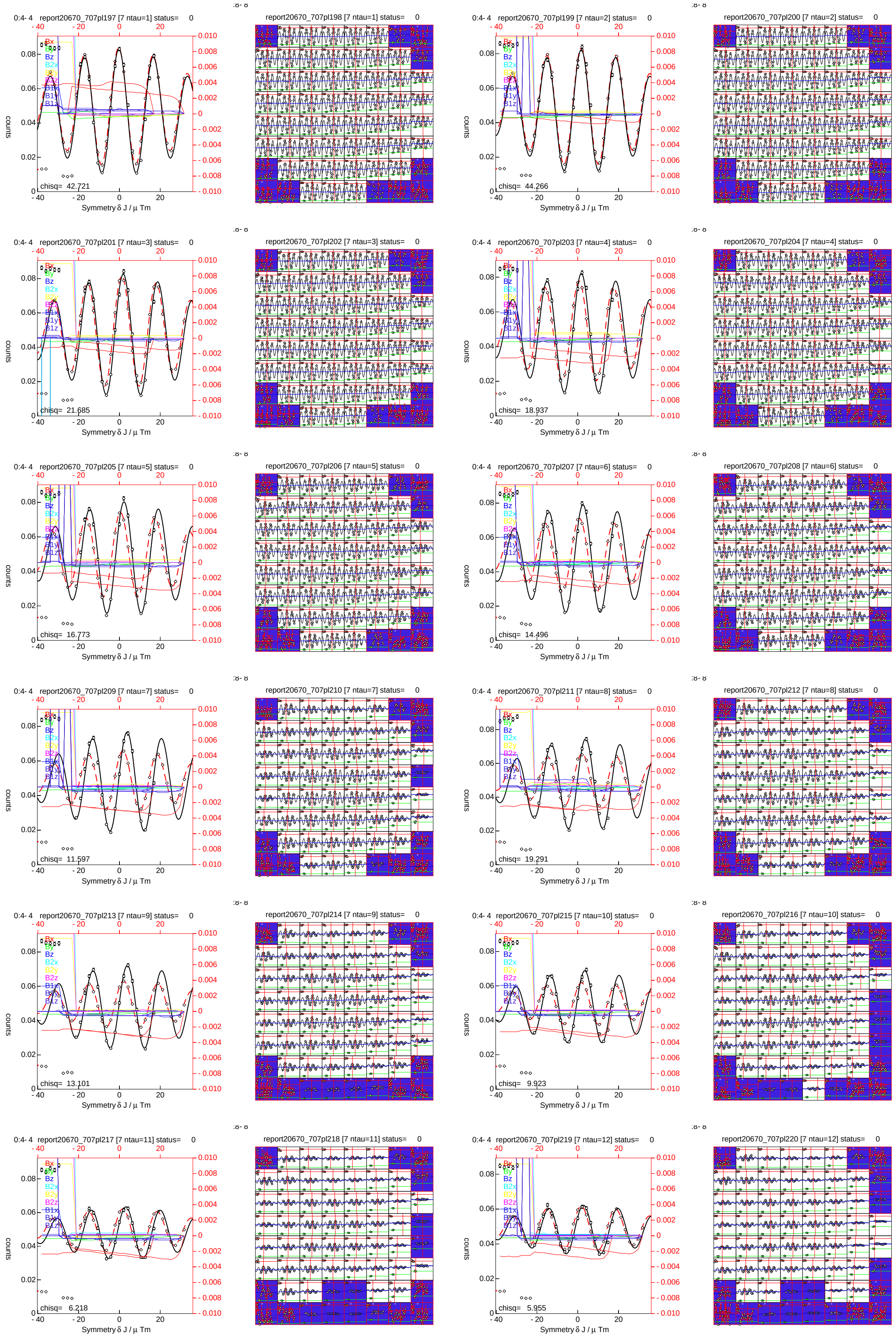

Figure 12: Center phase scan plots. Dashed red line + diamonds: scaled reference.

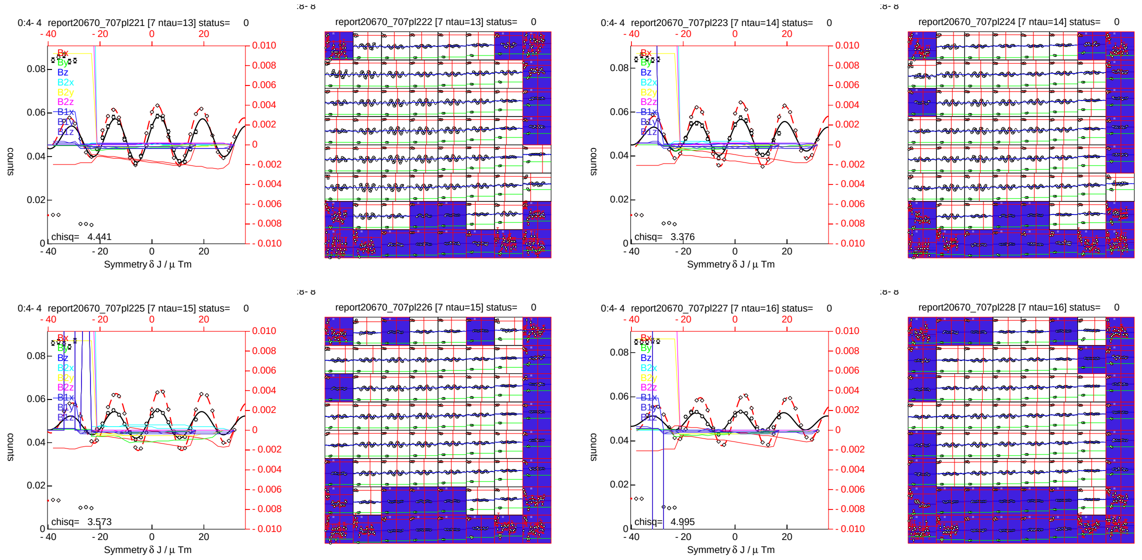

Figure 13: Continuation: Center phase scan plots. Dashed red line + diamonds: scaled reference.

20674 m20674.echo **SDS5wD2O Sun Jun 24 16:23:23 2018** SDS5wD2O "SDS with N

sample 16 pnts

SDS5wD2O "SDS with NaCl in D2O 0.05w in refpos, room temp"

SDS5wD2O "SDS with NaCl in D2O 0.05w in refpos, room temp"

matching reference: 20669

matching background: –

wavelength $\lambda = 8.0000$ Å

$Q/\text{Å}^{-1} = 0.1002$ ($2\theta = 7.3114$ ° $\Psi = -86.347$ °)

Up = 70.828 Down= 8.1545

Normalized echo amplitude (J_{min}, J_{max})

($\tau_{min} = 0.0948$ ns $\cdots$ $\tau_{max} = 69.724$ ns) at detector center: 0.9719 0.0269

#	J0/muTm	theta	nt	npic	nact	average0	<average>	<amplitu>	dj0/muTm	dja/muTm	djv/muTm	lam1/A	lam2/A	ref	pt	bgr	pt	distribution: dj-count per 1muTm
1	993.24	7.31	1	64	51	3.92899E+01	2.85032E+01	2.10444E+01	-0.4986	0.1038	1.2166	8.000	8.000	20669	1	--	--	| 0 0 0 0 0 6141212 7 0 0 0 0 0|
2	2555.60	7.31	1	64	51	3.91736E+01	2.85758E+01	2.04032E+01	1.9827	1.2415	0.7488	8.000	8.000	20669	2	--	--	| 0 0 0 0 0 1132215 0 0 0 0 0 0|
3	5167.16	7.31	1	64	51	3.91481E+01	2.85571E+01	1.92093E+01	1.8389	-0.4384	1.3182	8.000	8.000	20669	3	--	--	| 0 0 0 0 1 6131211 8 0 0 0 0 0|
4	10387.85	7.31	1	64	51	3.96114E+01	2.87158E+01	1.78125E+01	1.9496	-1.8477	3.0613	8.000	8.000	20669	4	--	--	| 0 0 1 3 411 6 6 6 4 5 4 0 0 1|
5	20828.60	7.31	1	64	51	3.92431E+01	2.87898E+01	1.50875E+01	2.1599	-4.0128	3.5445	8.000	8.000	20669	5	--	--	| 1 0 3 5 6 6 6 3 3 5 3 2 3 4 1|
6	31268.85	7.31	1	64	51	3.93433E+01	2.87907E+01	1.29329E+01	2.8149	-4.3010	4.6017	8.000	8.000	20669	6	--	--	| 1 4 4 3 5 7 3 2 3 3 5 1 1 4 5|
7	52150.05	7.31	1	64	49	3.94142E+01	2.96184E+01	1.01796E+01	3.6945	-5.1685	5.3679	8.000	8.000	20669	7	--	--	| 7 3 1 2 5 4 2 2 1 4 5 3 1 1 8|
8	73030.40	7.31	1	64	51	3.94896E+01	2.88153E+01	8.21676E+00	3.9705	-5.9071	6.2599	8.000	8.000	20669	8	--	--	|10 3 1 5 4 2 2 1 0 2 3 4 2 111|
9	104351.50	7.31	1	64	49	3.96023E+01	2.95755E+01	6.75732E+00	4.0655	-4.9061	8.4014	8.000	8.000	20669	9	--	--	|12 4 3 2 2 1 0 2 3 3 3 0 1 112|
10	156553.50	7.31	1	64	45	3.98960E+01	3.11980E+01	5.31246E+00	3.6423	-7.1541	6.8889	8.000	8.000	20669	10	--	--	| 9 3 2 3 3 2 2 0 0 2 4 2 3 010|
11	260957.50	7.31	1	64	47	3.99851E+01	2.99784E+01	3.22555E+00	2.9934	-7.0741	7.1221	8.000	8.000	20669	11	--	--	| 9 3 1 6 2 1 1 2 0 1 6 3 1 110|
12	313156.00	7.31	1	64	43	4.01789E+01	3.05942E+01	2.91273E+00	3.0160	-5.3619	7.5802	8.000	8.000	20669	12	--	--	| 7 3 1 4 2 1 2 1 3 4 4 1 0 1 9|
13	417560.00	7.31	1	64	44	3.97907E+01	3.08185E+01	2.00658E+00	2.5890	-3.5230	5.5160	8.000	8.000	20669	14	--	--	| 6 2 3 0 3 3 0 5 3 4 4 2 1 2 6|
14	521960.00	7.31	1	64	46	4.04407E+01	3.03098E+01	1.22310E+00	2.6819	-1.5758	6.9071	8.000	8.000	20669	16	--	--	| 7 3 2 0 1 2 1 3 4 6 1 4 3 3 6|
15	626359.00	7.31	1	64	40	4.04699E+01	3.12473E+01	8.53881E-01	2.6686	1.7165	6.5086	8.000	8.000	20669	17	--	--	| 5 4 3 1 1 2 1 4 3 3 3 2 4 0 4|
16	730756.50	7.31	1	64	35	4.07326E+01	3.33633E+01	6.08846E-01	2.2725	1.5086	7.5527	8.000	8.000	20669	18	--	--	| 5 1 2 2 1 2 1 0 4 4 3 2 1 2 5|

Table 10: Applied phase offsets and $10^6 \times \chi^2$ of aux. parameters (B_x, B_y,.. And Δ = difference ref-sample in 10^{-3} units (B:milliGauss).

nt	$\Delta J/\mu$Tm	$\simeq \Delta\Phi$	Bx	By	Bz	B2x	B2y	B2z	B1x	B1y	B1z
1	-0.201	$-4.2601°$	0.3∆ -0.6	0.0∆ -0.3	0.0∆ 0.5	0.0∆ 0.0	0.0∆ 0.2	0.0∆ -0.2	0.0∆ -0.0	0.0∆ -0.0	0.1
2	-0.092	$-1.9459°$	0.1∆ -0.4	0.0∆ -0.3	0.0∆ 0.6	0.0∆ 0.1	0.0∆ 0.0	0.0∆ -0.1	0.0∆ -0.0	0.0∆ 0.0	0.0
3	-0.388	$-8.2441°$	0.1∆ -0.8	0.0∆ 0.4	0.0∆ 0.6	0.0∆ 0.0	0.0∆ 0.3	0.1∆ -0.1	0.1∆ 0.1	0.0∆ -0.0	0.0
4	-0.053	$-1.1249°$	0.3∆ -0.2	0.0∆ -0.3	0.0∆ 0.6	0.0∆ 0.2	0.1∆ 0.0	0.0∆ -0.0	0.0∆ 0.0	0.0∆ 0.0	0.0
5	-0.147	$-3.1293°$	0.4∆ 0.0	0.0∆ -0.3	0.0∆ 0.6	0.1∆ 0.2	0.1∆ -0.0	0.0∆ -0.2	0.0∆ 0.1	0.1∆ 0.1	0.1
6	-0.213×10^{-2}	$-0.0453°$	0.5∆ -0.1	0.0∆ -0.3	0.0∆ 0.6	0.0∆ 0.3	0.0∆ -0.0	0.0∆ -0.0	0.0∆ 0.1	0.1∆ 0.0	0.0
7	-0.187	$-3.9631°$	0.8∆ -0.2	0.0∆ -0.3	0.0∆ 0.6	0.1∆ 0.2	0.0∆ -0.1	0.0∆ -0.1	0.0∆ 0.0	0.0∆ 0.0	0.1
8	-0.290	$-6.1648°$	0.9∆ -0.8	0.0∆ 0.3	0.0∆ 0.6	0.1∆ -0.2	0.0∆ 0.4	0.0	0.1∆ 0.0	0.0∆ 0.0	0.0
9	-0.143×10^{-2}	$-0.0303°$	1.2∆ -0.3	0.0∆ -0.3	0.0∆ 0.7	0.0∆ 0.2	0.1∆ 0.1	0.0∆ -0.0	0.1∆ -0.0	0.1∆ -0.0	0.0
10	0.071	$1.5104°$	1.0∆ -0.5	0.0∆ -0.3	0.0∆ 0.6	0.0∆ 0.1	0.0∆ 0.1	0.0∆ -0.2	0.0∆ -0.0	0.0∆ 0.0	0.1
11	-0.194	$-4.1175°$	0.7∆ -0.5	0.0∆ -0.3	0.0∆ 0.6	0.0∆ -0.1	0.0∆ 0.2	0.0∆ -0.0	0.1∆ -0.1	0.0∆ -0.0	0.0
12	-0.012	$-0.2650°$	0.3∆ -0.7	0.0∆ -0.1	0.0∆ 0.6	0.0∆ -0.0	0.0∆ 0.2	0.0∆ -0.1	0.1∆ -0.0	0.0∆ -0.0	0.0
13	-0.184	$-3.9079°$	0.3∆ -0.5	0.0∆ -0.2	0.0∆ 0.5	0.0∆ 0.0	0.0∆ 0.2	0.1∆ -0.1	0.0∆ 0.0	0.1∆ -0.0	0.0
14	-0.067	$-1.4267°$	0.2∆ -0.5	0.0∆ -0.2	0.0∆ 0.5	0.0∆ -0.0	0.1∆ 0.3	0.0∆ -0.2	0.0∆ -0.0	0.0∆ 0.0	0.1
15	0.016	$0.3349°$	0.0∆ -0.8	0.0∆ 0.1	0.0∆ 0.6	0.1∆ -0.4	0.0∆ 0.4	0.0∆ -0.0	0.1∆ -0.0	0.0∆ -0.0	0.0
16	-0.236	$-5.0190°$	0.0∆ -0.6	0.0∆ 0.1	0.0∆ 0.5	0.0∆ 0.0	0.0∆ 0.3	0.1∆ 0.0	0.1∆ 0.0	0.0∆ 0.0	0.1
Ave	-0.1230	$-2.6124°$	-	-	-	-	-	-	-	-	-
Var	0.1220	$2.5907°$	-	-	-	-	-	-	-	-	-

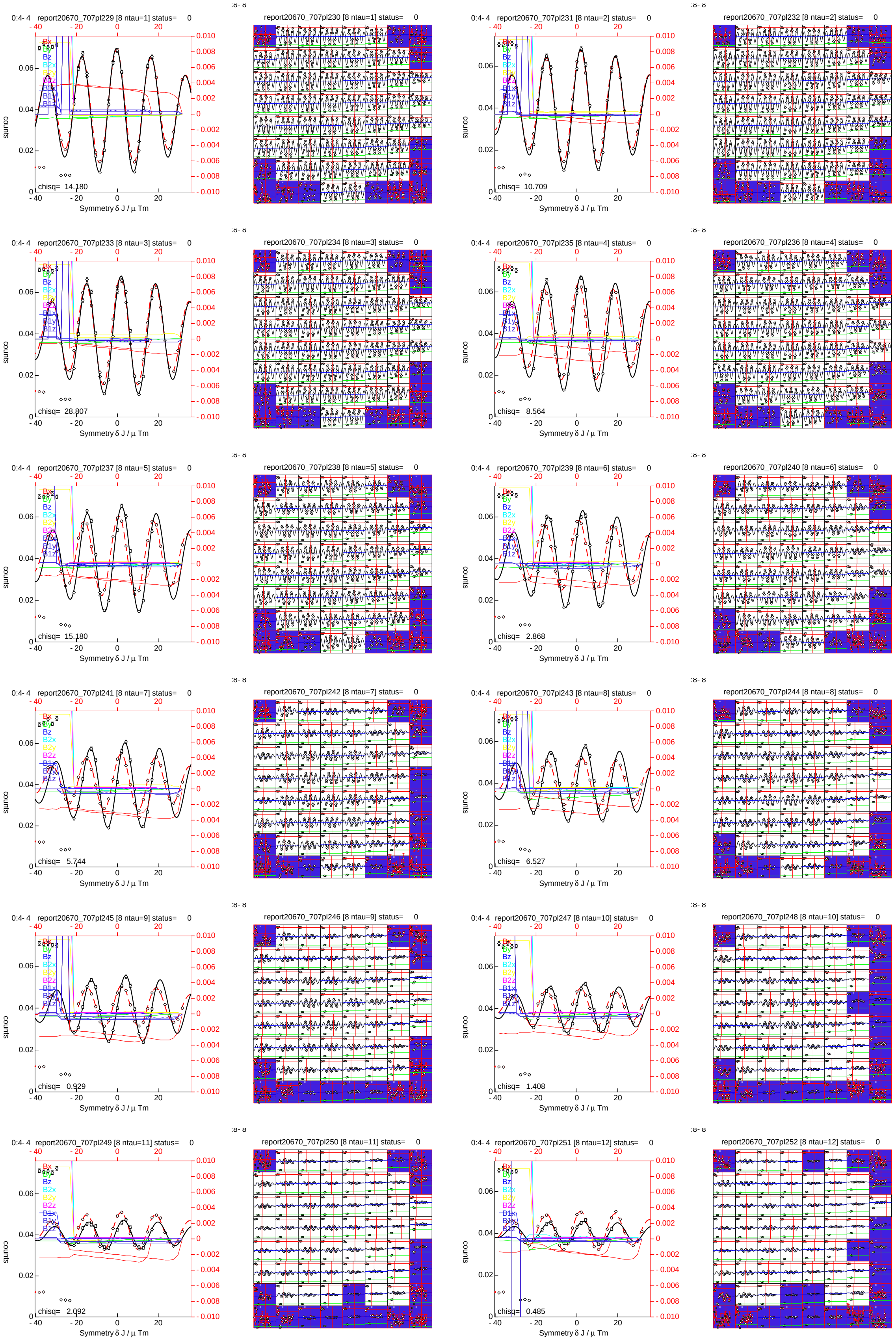

Figure 14: Center phase scan plots. Dashed red line + diamonds: scaled reference.

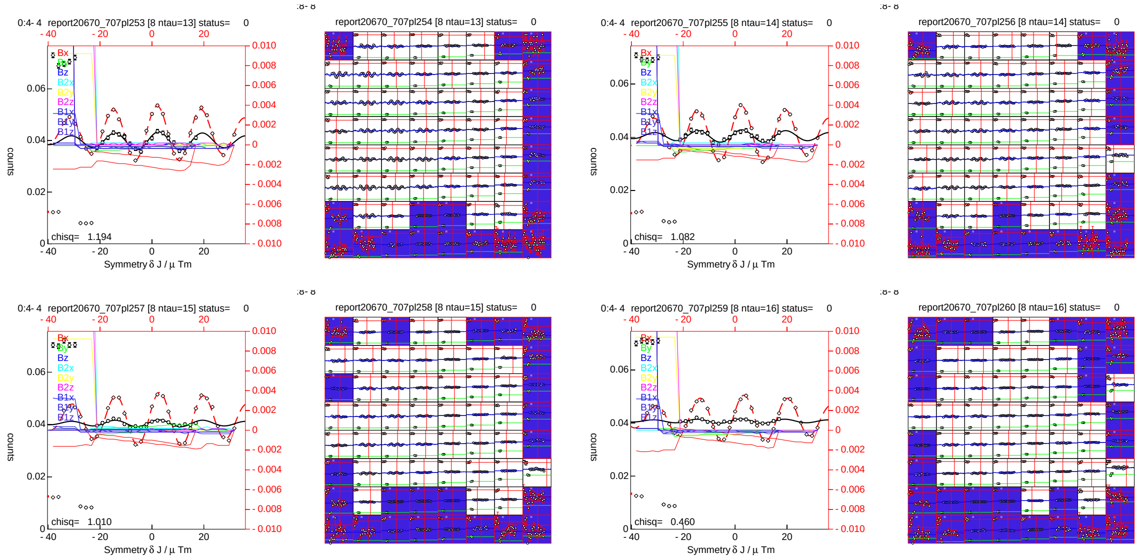

Figure 15: Continuation: Center phase scan plots. Dashed red line + diamonds: scaled reference.

20675 m20675.echo **SDS5wD2O Sun Jun 24 19:04:37 2018** SDS5wD2O "SDS with N

sample 13 pnts

SDS5wD2O "SDS with NaCl in D2O 0.05w in refpos, room temp"

SDS5wD2O "SDS with NaCl in D2O 0.05w in refpos, room temp"

matching reference: 20676

matching background: –

wavelength $\lambda = 8.0000$ Å

$Q/\text{Å}^{-1} = 0.1501$ ($2\theta = 10.967$ ° $\Psi = -84.510$ °)

Up = 19.363 Down= 2.7725

Normalized echo amplitude (J_{min}, J_{max})

($\tau_{min} = 0.0948$ ns $\cdots$ $\tau_{max} = 39.841$ ns) at detector center: 0.9471 0.0164

```
 #      J0/muTm   theta  nt npic nact      average0     <average>     <amplitu> dj0/muTm dja/muTm djv/muTm    lam1/A     lam2/A    ref pt    bgr pt   distribution: dj-count per 1muTm
 1       994.03   10.97   1   64   55  1.09562E+01  7.66671E+00  5.25088E+00  -0.6361   0.3354   1.3071     8.000      8.000  20676  1     -- --   | 0 0 0 0 0 9131212 9 0 0 0 0 0|
 2      2555.51   10.97   1   64   55  1.09085E+01  7.66580E+00  5.03753E+00   2.3647   1.7338   0.6463     8.000      8.000  20676  2     -- --   | 0 0 0 0 0 0123013 0 0 0 0 0 0|
 3      5167.14   10.97   1   64   55  1.08146E+01  7.66202E+00  4.59975E+00   2.1899  -0.0940   1.3121     8.000      8.000  20676  3     -- --   | 0 0 0 0 0 7151212 9 0 0 0 0 0|
 4     10387.85   10.97   1   64   55  1.09156E+01  7.69952E+00  3.99634E+00   2.1950  -2.0552   2.4590     8.000      8.000  20676  4     -- --   | 0 0 1 2 6 910 5 4 8 3 6 1 0 0|
 5     20828.60   10.97   1   64   54  1.09361E+01  7.84026E+00  3.12318E+00   2.1913  -4.1732   3.6974     8.000      8.000  20676  5     -- --   | 2 0 3 3 9 5 7 4 1 5 5 1 3 4 2|
 6     31268.85   10.97   1   64   55  1.09794E+01  7.71220E+00  2.40433E+00   3.0070  -4.7441   4.4330     8.000      8.000  20676  6     -- --   | 2 3 2 7 3 7 7 3 0 5 2 4 1 2 7|
 7     52150.00   10.97   1   64   52  1.09741E+01  8.03749E+00  1.59681E+00   3.7734  -5.0374   6.2834     8.000      8.000  20676  7     -- --   | 8 4 2 2 5 3 4 0 0 7 2 4 0 2 9|
 8     73030.30   10.97   1   64   55  1.10228E+01  7.70218E+00  1.09171E+00   4.3430  -5.3973   6.6503     8.000      8.000  20676  8     -- --   |10 5 0 4 5 2 3 0 0 4 2 4 1 213|
 9    104351.50   10.97   1   64   53  1.09871E+01  7.90832E+00  6.91168E-01   3.8912  -5.9945   8.0578     8.000      8.000  20676  9     -- --   |14 1 3 3 4 1 2 0 0 4 2 6 0 112|
10    156553.50   10.97   1   64   49  1.11701E+01  8.44432E+00  3.87476E-01   3.7178  -6.6857   7.7692     8.000      8.000  20676 10     -- --   |13 1 3 3 3 2 1 0 0 5 1 4 1 111|
11    260957.50   10.97   1   64   44  1.10131E+01  8.58897E+00  1.39264E-01   3.6079  -6.3347   6.9156     8.000      8.000  20676 11     -- --   | 7 3 2 5 2 2 0 2 0 3 4 2 2 1 9|
12    313156.00   10.97   1   64   45  1.12247E+01  8.54841E+00  7.57074E-02   3.7602  -5.1371   6.2611     8.000      8.000  20676 12     -- --   | 5 4 2 3 4 3 0 2 2 4 3 3 1 0 9|
13    417561.00   10.97   1   64   45  1.12174E+01  8.66767E+00  1.40534E-02   4.1378  -2.1317   5.7710     8.000      8.000  20676 14     -- --   | 7 1 1 4 2 2 1 4 3 5 3 3 1 1 7|
```

Table 11: Applied phase offsets and $10^6 \times \chi^2$ of aux. parameters (B_x, B_y,.. And Δ = difference ref-sample in 10^{-3} units (B:milliGauss).

nt	$\Delta J/\mu$Tm	$\simeq \Delta\Phi$	Bx	By	Bz	B2x	B2y	B2z	B1x	B1y	B1z
1	−0.168	−3.5719°	0.1Δ -0.4	0.0Δ -0.1	0.0Δ -0.1	0.0Δ -0.7	0.0Δ 1.1	0.0Δ -0.1	0.0Δ -0.0	0.0Δ -0.1	0.0
2	−0.083	−1.7545°	0.0Δ 0.2	0.0Δ -0.2	0.0Δ -0.2	0.0Δ -0.7	0.0Δ 1.1	0.0Δ -0.0	0.0Δ -0.0	0.1Δ -0.0	0.0
3	−0.272	−5.7799°	0.0Δ 0.4	0.0Δ -0.2	0.0Δ -0.2	0.0Δ -0.9	0.1Δ 1.0	0.0Δ -0.2	0.0Δ 0.0	0.1Δ 0.0	0.0
4	−0.113	−2.3918°	0.1Δ 0.4	0.0Δ -0.2	0.0Δ 0.1	0.0Δ -0.7	0.0Δ 0.9	0.0Δ 0.1	0.0Δ -0.0	0.0Δ 0.0	0.1
5	−0.459	−9.7438°	0.3Δ 0.4	0.5Δ -0.5	0.0Δ 0.8	0.1Δ -0.6	0.1Δ 0.8	0.0Δ 0.3	0.0Δ 0.0	0.0Δ 0.1	0.0
6	−0.162	−3.4459°	0.2Δ 0.5	0.0Δ -0.4	0.0Δ 0.7	0.0Δ -0.7	0.0Δ 0.9	0.0Δ 0.2	0.0Δ 0.1	0.0Δ 0.2	0.1
7	−0.488	−10.356°	0.6Δ 0.6	0.0Δ -0.4	0.0Δ 0.8	0.1Δ -0.3	0.0Δ 0.5	0.0Δ 0.2	0.1Δ 0.1	0.0Δ 0.1	0.0
8	−0.265	−5.6262°	0.4Δ 0.8	0.0Δ -0.4	0.0Δ 0.7	0.1Δ -0.1	0.0Δ 0.5	0.1Δ 0.1	0.0Δ 0.1	0.0Δ 0.2	0.0
9	−0.508	−10.781°	0.4Δ 1.1	0.0Δ -0.4	0.0Δ 0.7	0.1Δ -0.3	0.1Δ 0.4	0.0	0.0Δ 0.2	0.0Δ 0.2	0.1
10	−0.269	−5.7103°	0.4Δ 1.3	0.0Δ -0.2	0.0Δ 0.7	0.0Δ -0.1	0.0Δ 0.2	0.0Δ -0.0	0.0Δ 0.2	0.0Δ 0.3	0.1
11	0.411	8.7248°	0.3Δ 1.2	0.0Δ 0.0	0.0Δ 0.7	0.0Δ 0.1	0.0Δ 0.0	0.0Δ -0.2	0.0Δ 0.4	0.1Δ 0.3	0.0
12	0.666	14.134°	0.2Δ 1.0	0.0Δ 0.0	0.0Δ 0.7	0.1Δ 0.0	0.1Δ 0.1	0.0Δ -0.2	0.0Δ 0.3	0.1Δ 0.3	0.1
13	1.001	21.249°	0.1Δ 0.6	0.0Δ -0.3	0.0Δ 0.8	0.0Δ 0.3	0.0Δ -0.2	0.0Δ -0.4	0.1Δ 0.2	0.0Δ 0.1	0.0
Ave	−0.0545	−1.1579°	-	-	-	-	-	-	-	-	-
Var	0.4448	9.4442°	-	-	-	-	-	-	-	-	-

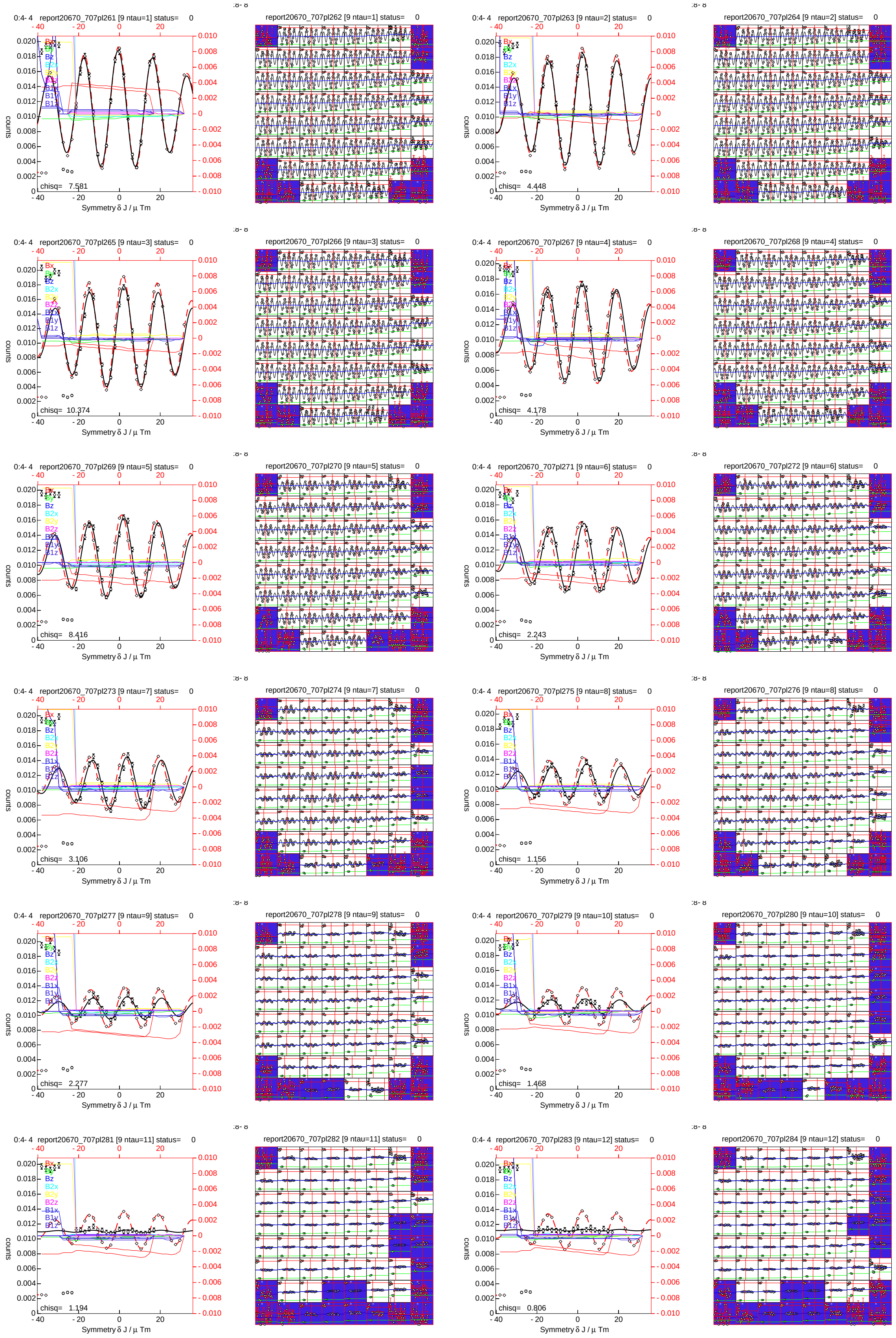

Figure 16: Center phase scan plots. Dashed red line + diamonds: scaled reference.

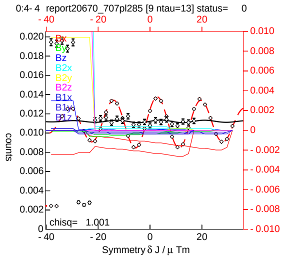

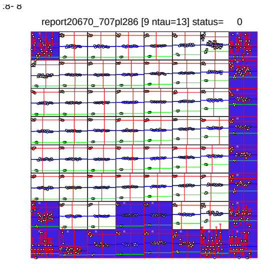

Figure 17: Continuation: Center phase scan plots. Dashed red line + diamonds: scaled reference.

SDS5wD2O_"SDS_with_NaCl_in_D2O [6, 800]
Without background subtraction

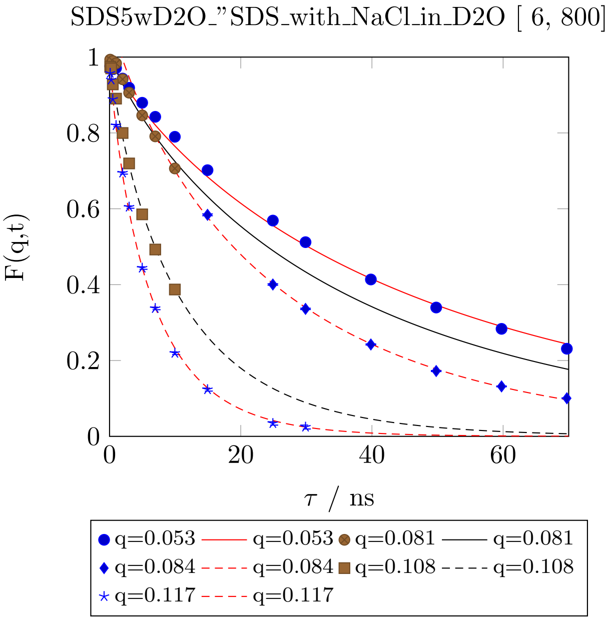

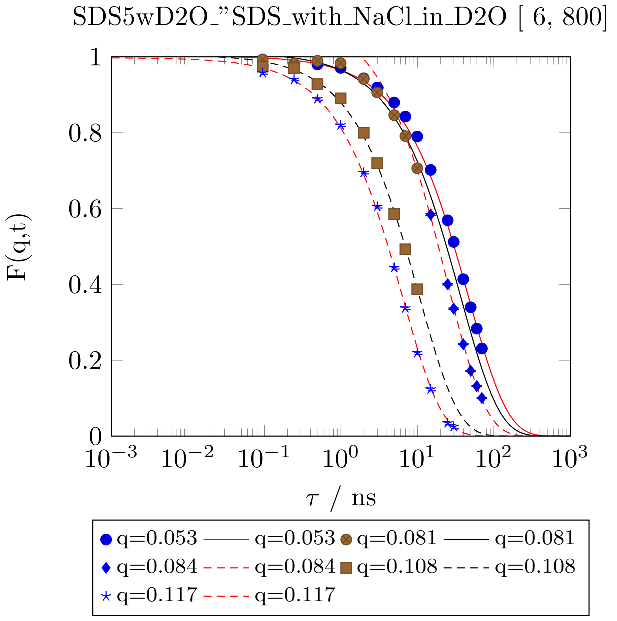

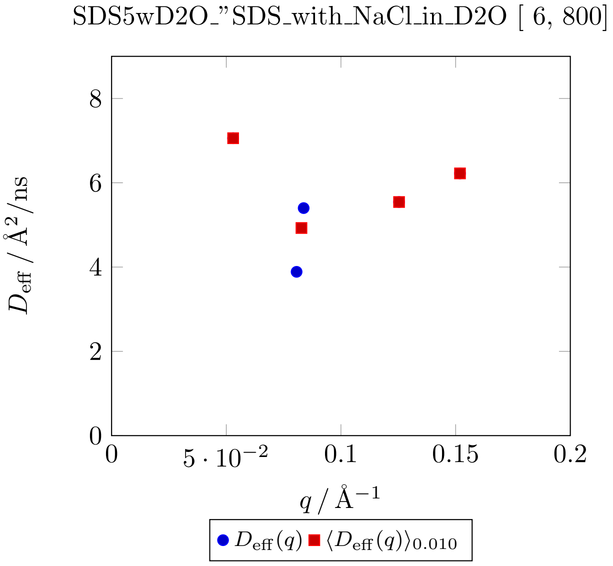

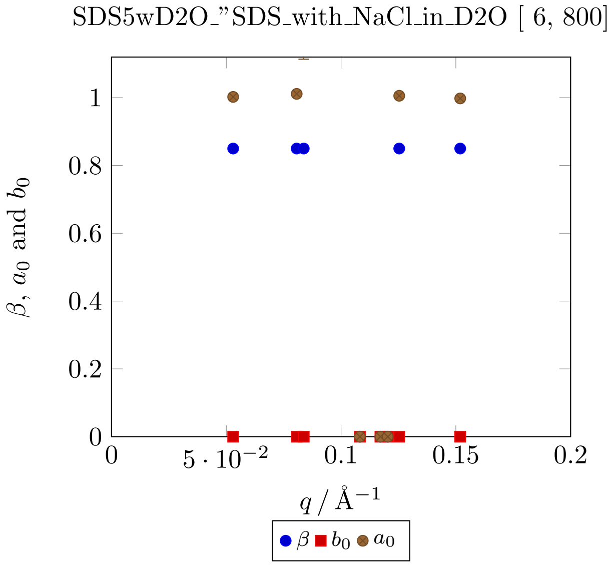

Fitting model:

$$F(q,t) = a_0\ \left[\exp\{-(t/\tau)^{\beta}\}\right] + b_0 \qquad (1)$$

Expression for the effective diffusion constant:

$$D_{\mathrm{eff}}(q)\ =\ \frac{1}{(\tau_0/\beta)\Gamma(1/\beta)\ q^2} \qquad (2)$$

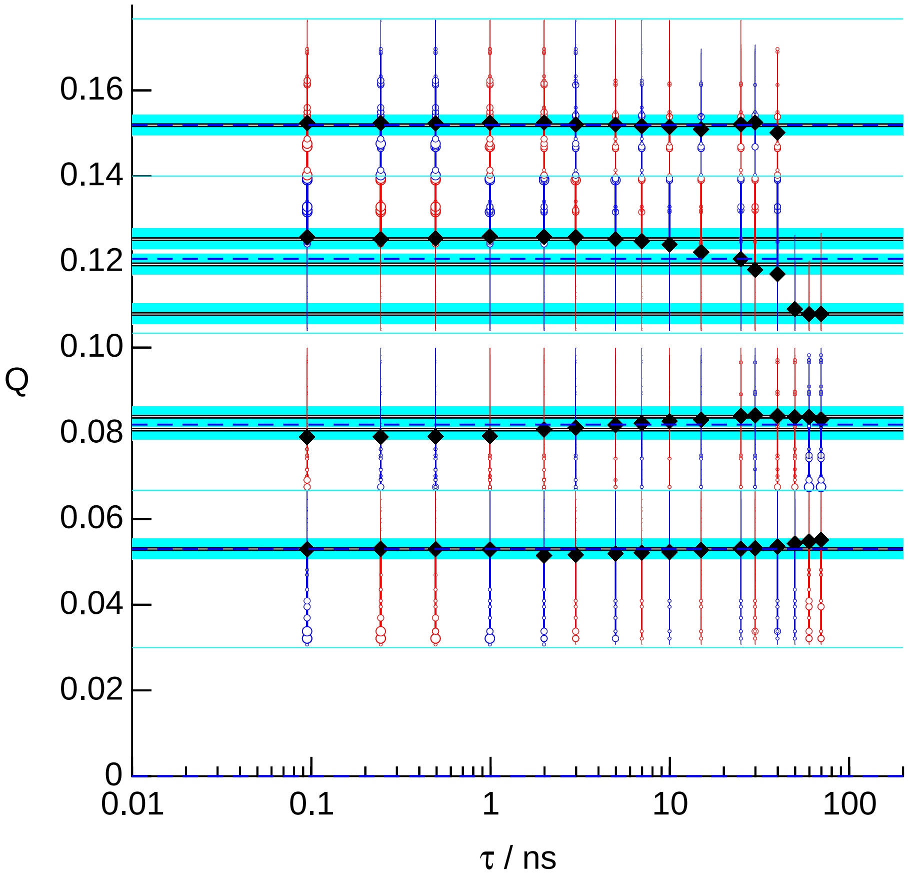

Figure 18: Mapping of single pixel contributions to $S(q,t)$ points: black diamonds. Dashed lines indicate the average q values in the q-binning zones. Size of star rays end symbols (circles) indicate the weight of the contribution from the end symbol (q,t). Cyan stripes indicate the catch zone for postcollection used in order to yield curves with consistent q-labels (controlled by parameter *r.postcoll_qcat*.

All underlying data of this plot group are preset as ASCII tables in file report20670_707.tex The corresponding sections start with a comment line: %%+++DRSPINE-PLOT:0: ...
Data in datreat format are copied to: report20670_707.1.dtr .

SDS5wD2O_"SDS_with_NaCl_in_D2O [8, 800]
Without background subtraction

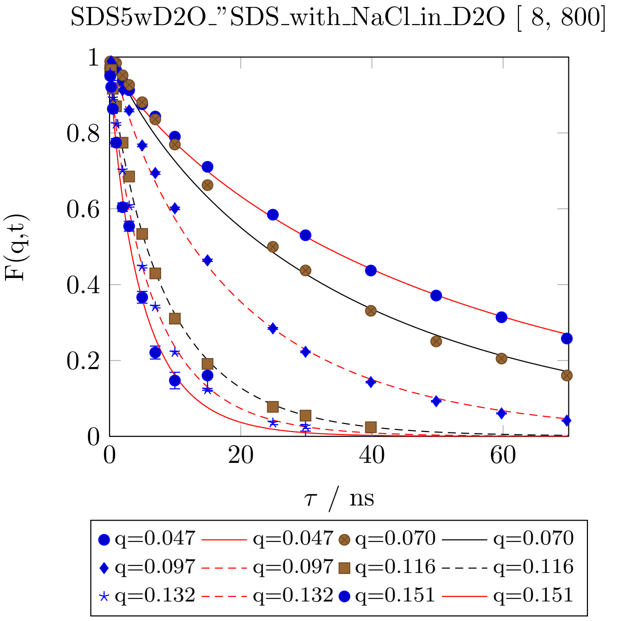

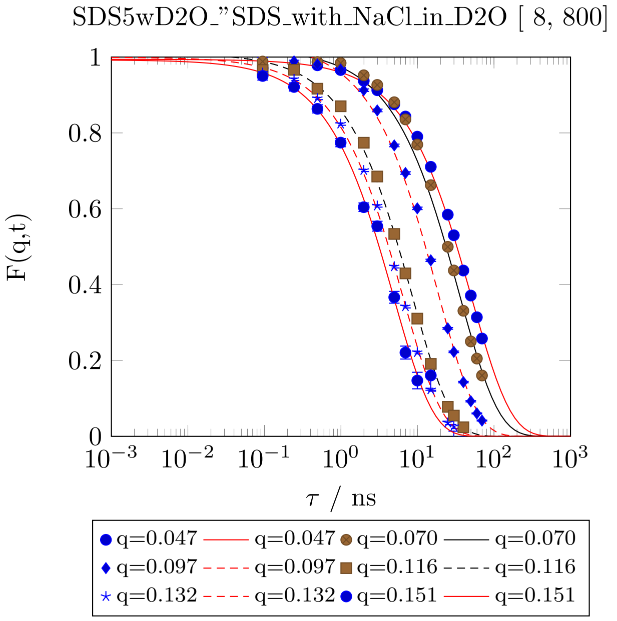

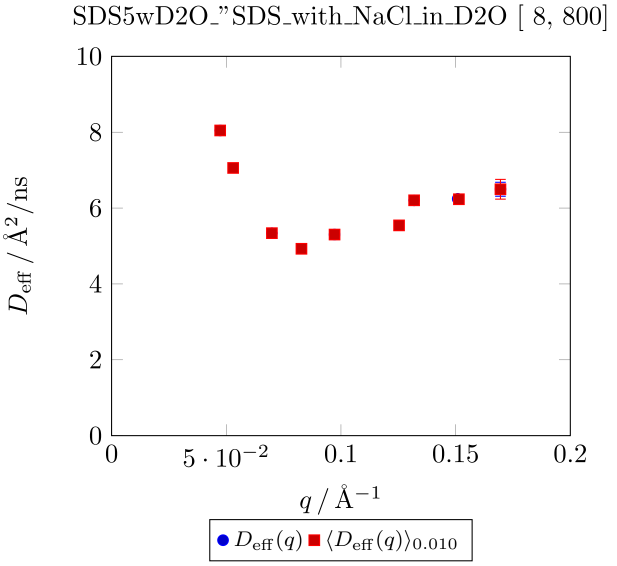

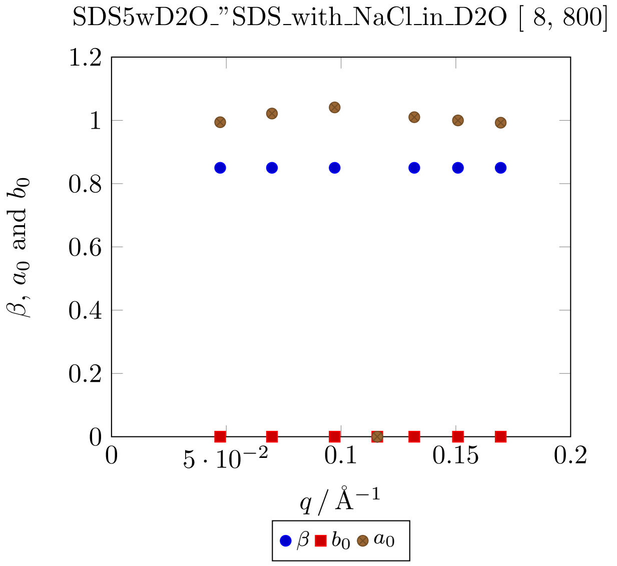

Fitting model:

$$F(q,t) = a_0 \ \left[\exp\{-(t/\tau)^{\beta}\}\right] + b_0 \tag{3}$$

Expression for the effective diffusion constant:

$$D_{\text{eff}}(q) \ = \ \frac{1}{(\tau_0/\beta)\Gamma(1/\beta)\ q^2} \tag{4}$$

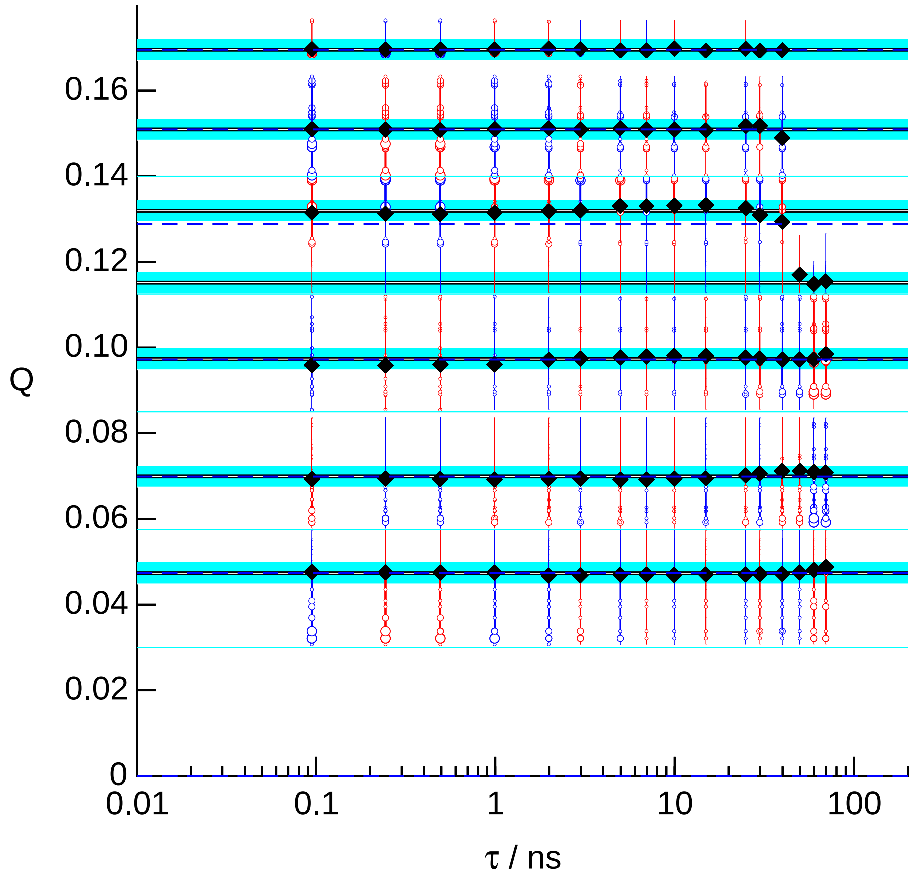

Figure 19: Mapping of single pixel contributions to $S(q,t)$ points: black diamonds. Dashed lines indicate the average q values in the q-binning zones. Size of star rays end symbols (circles) indicate the weight of the contribution from the end symbol (q,t). Cyan stripes indicate the catch zone for postcollection used in order to yield curves with consistent q-labels (controlled by parameter *r.postcoll_qcat*.

All underlying data of this plot group are preset as ASCII tables in file report20670_707.tex The corresponding sections start with a comment line: %%+++DRSPINE-PLOT:1: ...
Data in datreat format are copied to: report20670_707.2.dtr .

SDS5wD2O_"SDS_with_NaCl_in_D2O [10, 800]
Without background subtraction

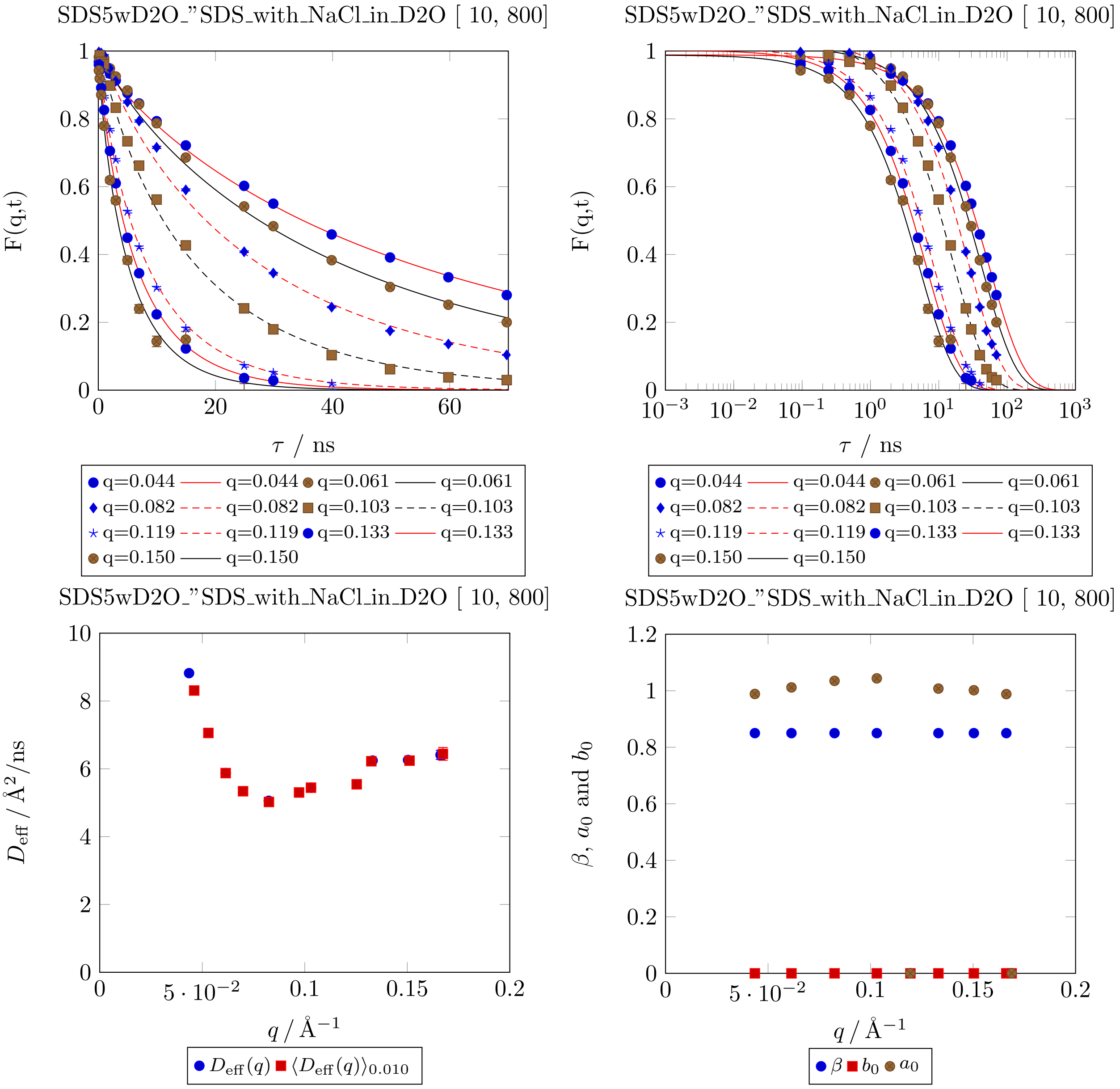

Fitting model:

$$F(q,t) = a_0 \; \left[\exp\{-(t/\tau)^{\beta}\}\right] + b_0 \tag{5}$$

Expression for the effective diffusion constant:

$$D_{\mathrm{eff}}(q) \;=\; \frac{1}{(\tau_0/\beta)\Gamma(1/\beta)\; q^2} \tag{6}$$

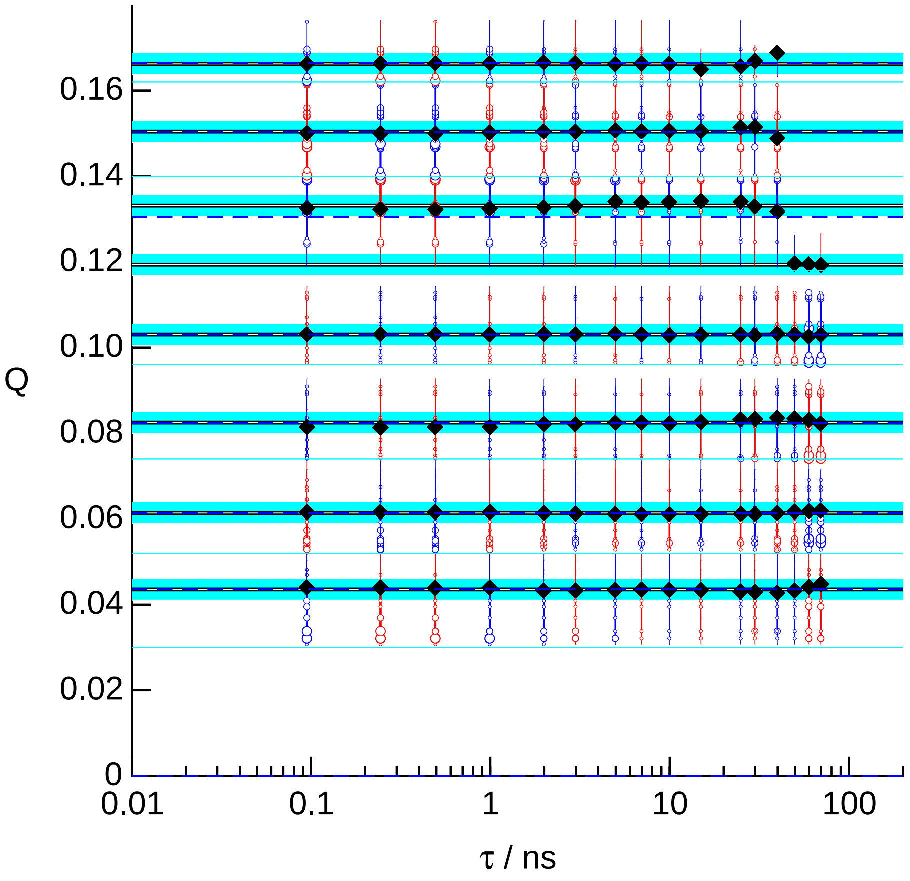

Figure 20: Mapping of single pixel contributions to $S(q,t)$ points: black diamonds. Dashed lines indicate the average q values in the q-binning zones. Size of star rays end symbols (circles) indicate the weight of the contribution from the end symbol (q,t). Cyan stripes indicate the catch zone for postcollection used in order to yield curves with consistent q-labels (controlled by parameter *r.postcoll_qcat*.

All underlying data of this plot group are preset as ASCII tables in file report20670_707.tex The corresponding sections start with a comment line: %%+++DRSPINE-PLOT:2: ...
Data in datreat format are copied to: report20670_707.3.dtr .

SDS5wD2O_"SDS_with_NaCl_in_D2O [12, 800]
Without background subtraction

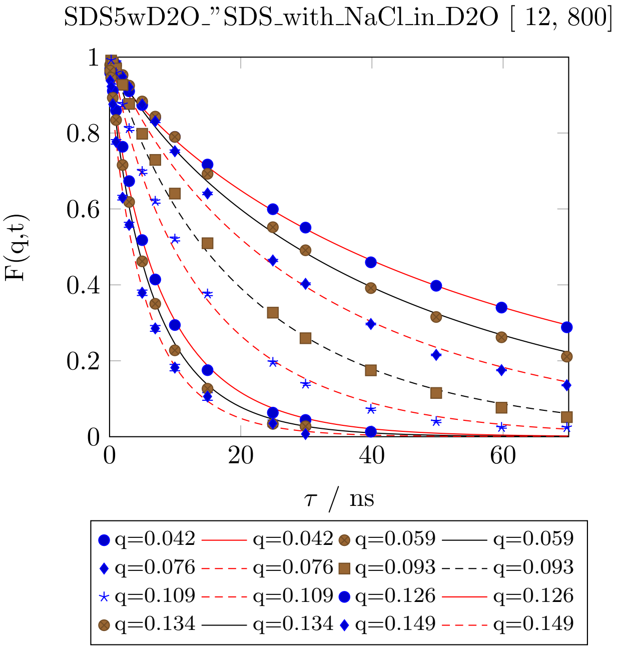

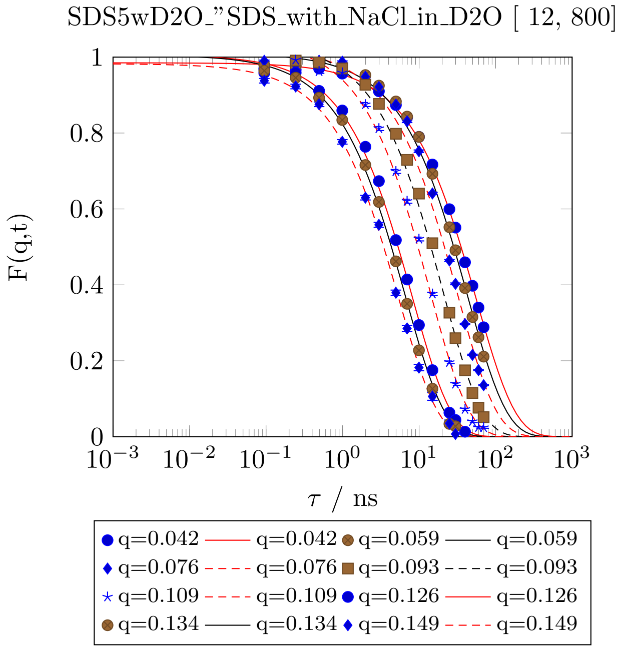

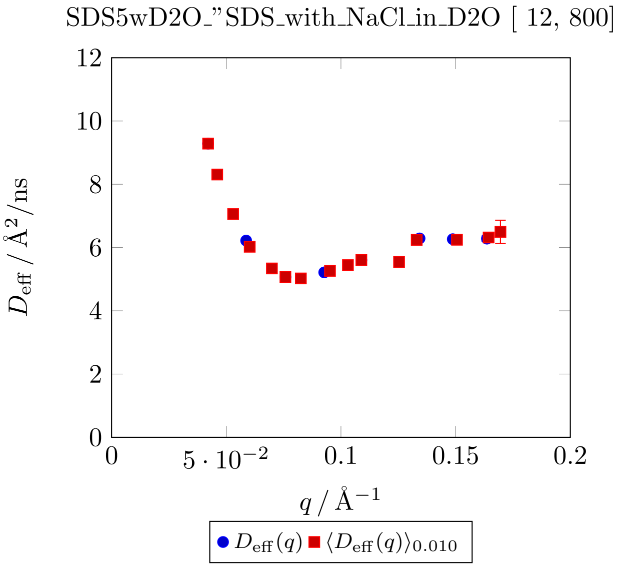

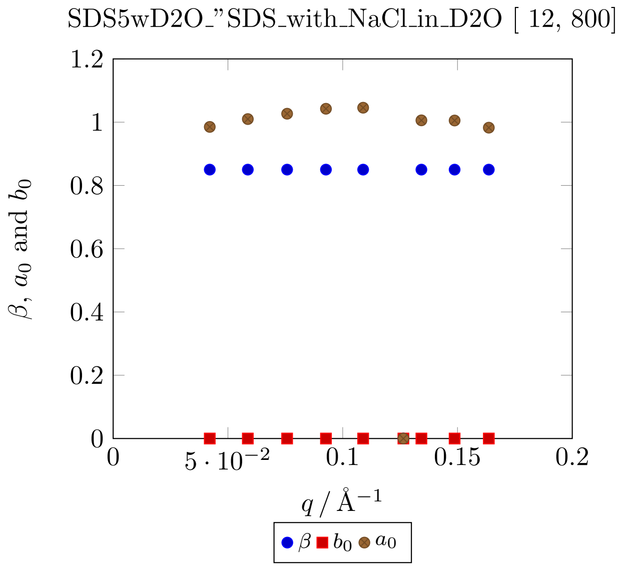

Fitting model:

$$F(q,t) = a_0\ \left[\exp\{-(t/\tau)^{\beta}\}\right] + b_0 \tag{7}$$

Expression for the effective diffusion constant:

$$D_{\mathrm{eff}}(q)\ =\ \frac{1}{(\tau_0/\beta)\Gamma(1/\beta)\ q^2} \tag{8}$$

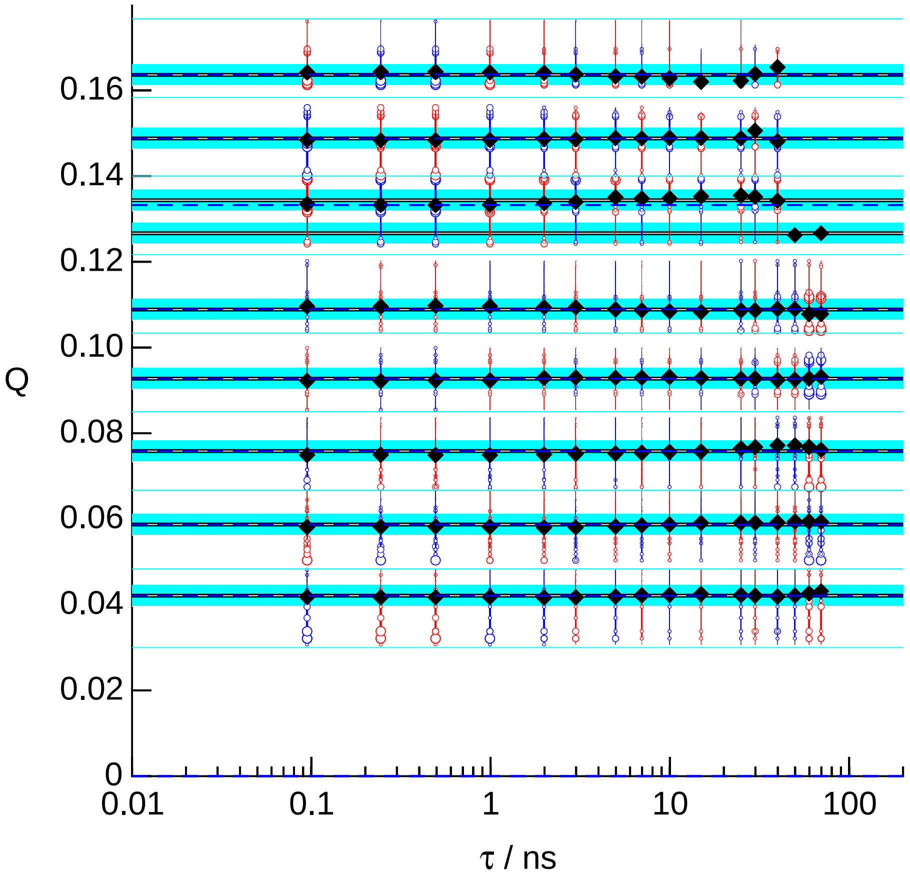

Figure 21: Mapping of single pixel contributions to $S(q,t)$ points: black diamonds. Dashed lines indicate the average q values in the q-binning zones. Size of star rays end symbols (circles) indicate the weight of the contribution from the end symbol (q,t). Cyan stripes indicate the catch zone for postcollection used in order to yield curves with consistent q-labels (controlled by parameter *r.postcoll_qcat*.

All underlying data of this plot group are preset as ASCII tables in file report20670_707.tex The corresponding sections start with a comment line: %%+++DRSPINE-PLOT:3: ...
Data in datreat format are copied to: report20670_707.4.dtr .

SDS5wD2O_"SDS_with_NaCl_in_D2O [14, 800]
Without background subtraction

SDS5wD2O_"SDS_with_NaCl_in_D2O [14, 800]

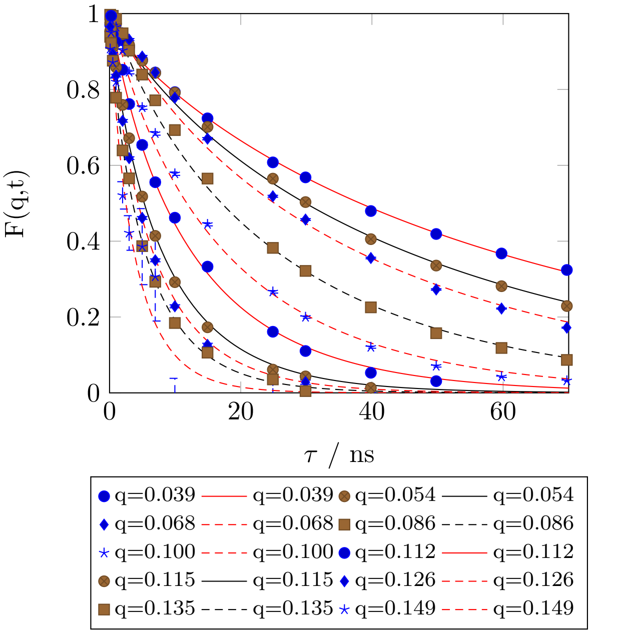

SDS5wD2O_"SDS_with_NaCl_in_D2O [14, 800]

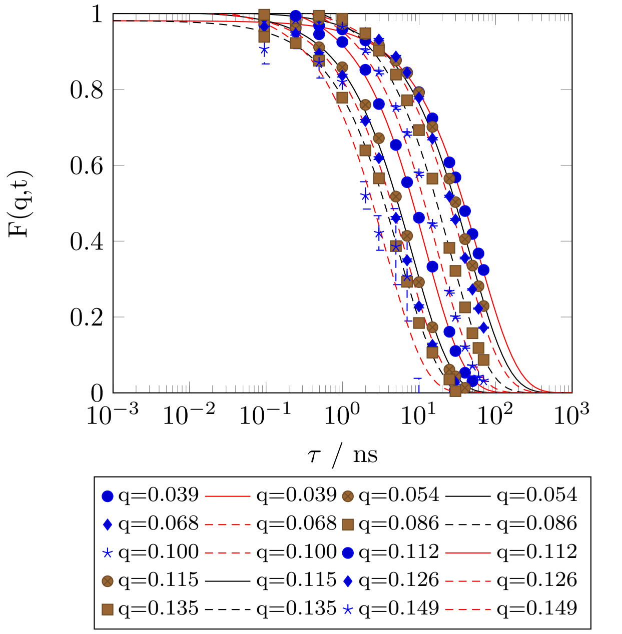

SDS5wD2O_"SDS_with_NaCl_in_D2O [14, 800]

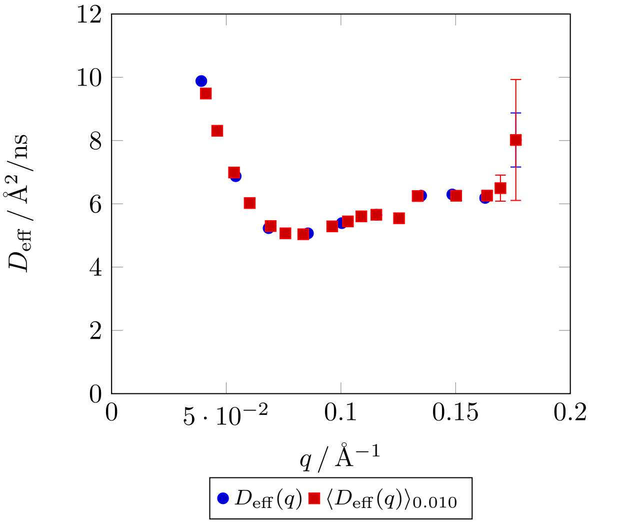

SDS5wD2O_"SDS_with_NaCl_in_D2O [14, 800]

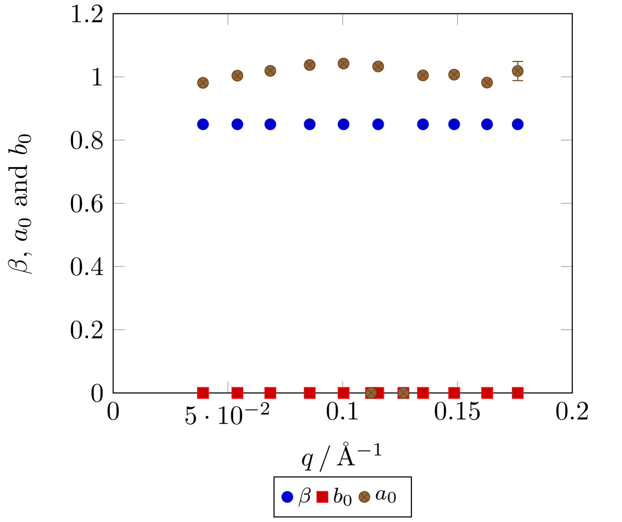

Fitting model:

$$F(q,t) = a_0 \ \left[\exp\{-(t/\tau)^{\beta}\}\right] + b_0 \tag{9}$$

Expression for the effective diffusion constant:

$$D_{\mathrm{eff}}(q) \ = \ \frac{1}{(\tau_0/\beta)\Gamma(1/\beta)\ q^2} \tag{10}$$

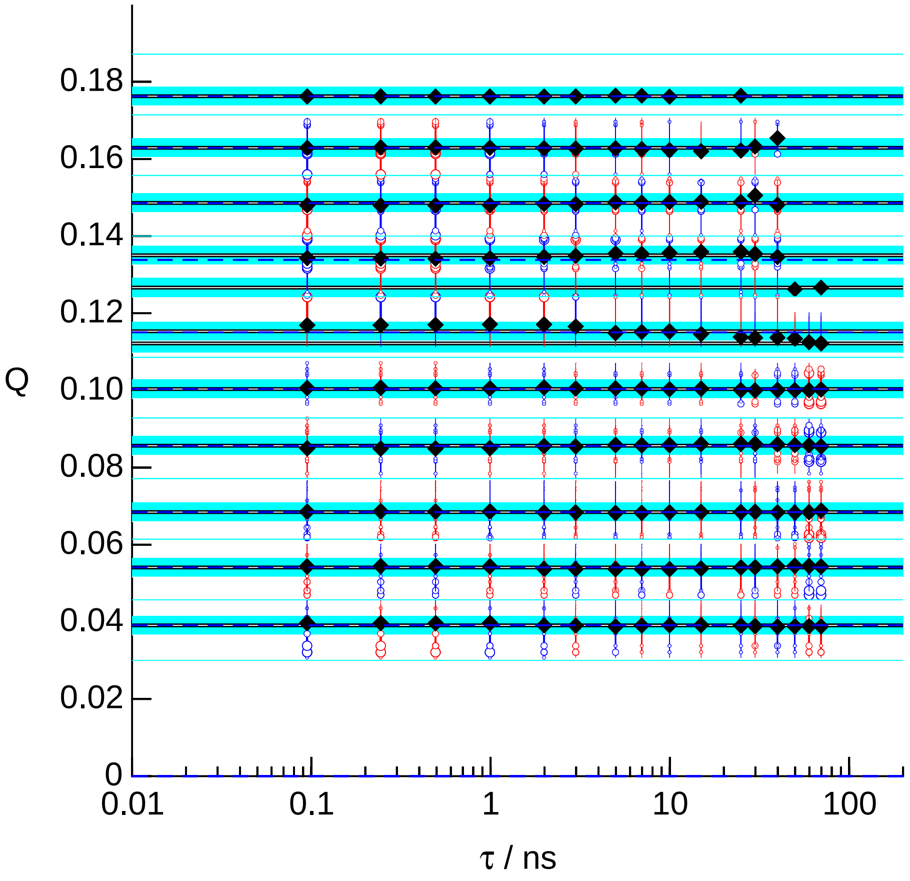

Figure 22: Mapping of single pixel contributions to $S(q,t)$ points: black diamonds. Dashed lines indicate the average q values in the q-binning zones. Size of star rays end symbols (circles) indicate the weight of the contribution from the end symbol (q,t). Cyan stripes indicate the catch zone for postcollection used in order to yield curves with consistent q-labels (controlled by parameter *r.postcoll_qcat*.

All underlying data of this plot group are preset as ASCII tables in file report20670_707.tex The corresponding sections start with a comment line: %%+++DRSPINE-PLOT:4: ...
Data in datreat format are copied to: report20670_707.5.dtr .

SDS5wD2O_"SDS_with_NaCl_in_D2O [16, 800]
Without background subtraction

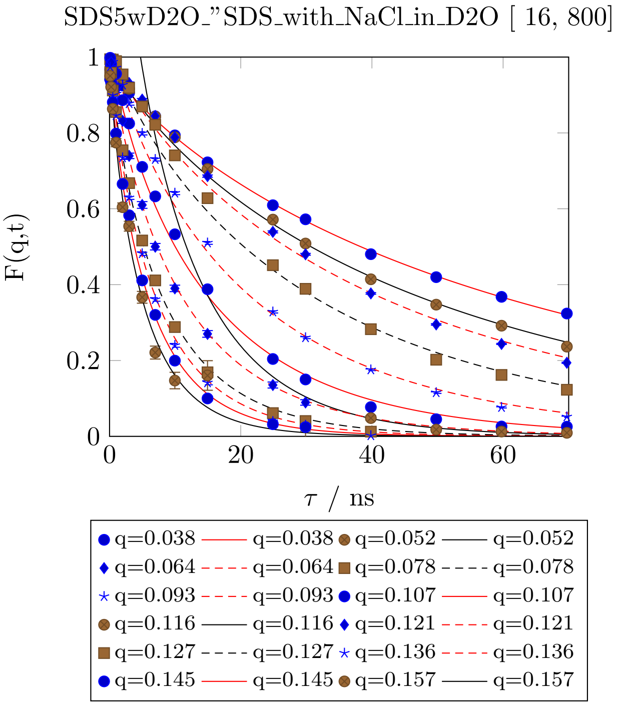

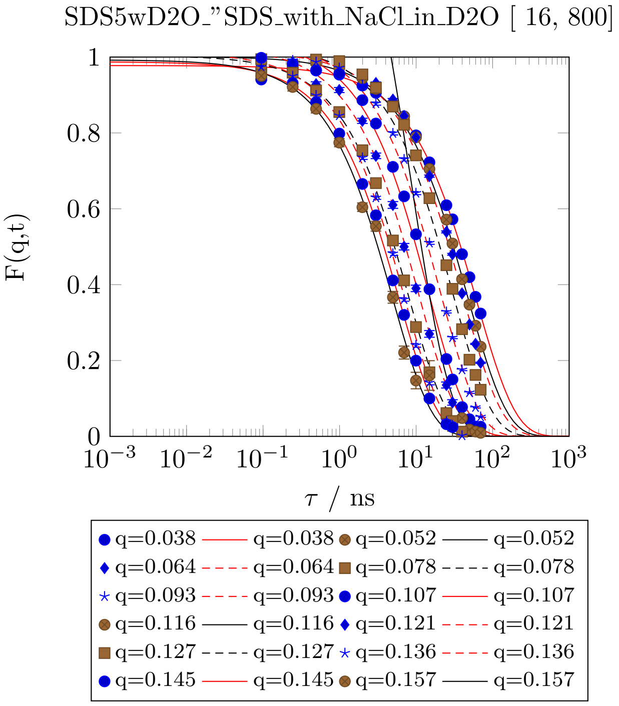

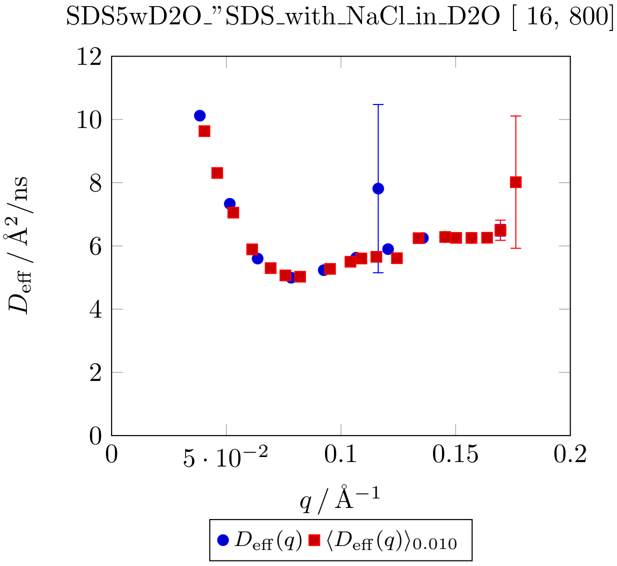

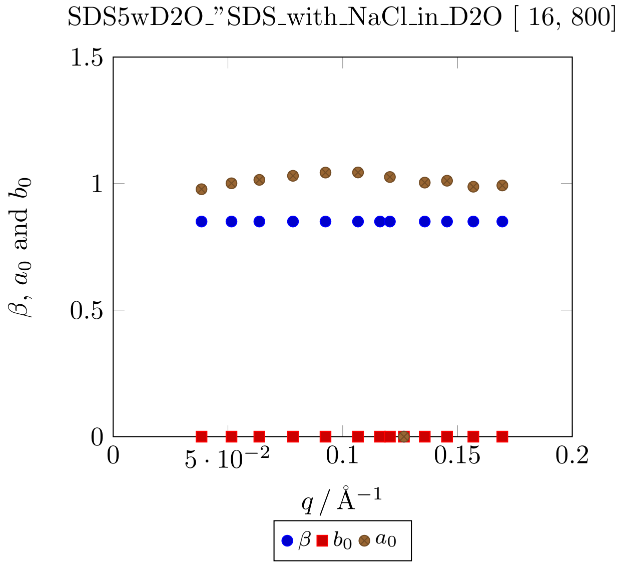

Fitting model:

$$F(q,t) = a_0 \left[\exp\{-(t/\tau)^{\beta}\}\right] + b_0 \tag{11}$$

Expression for the effective diffusion constant:

$$D_{\mathrm{eff}}(q) = \frac{1}{(\tau_0/\beta)\Gamma(1/\beta)\, q^2} \tag{12}$$

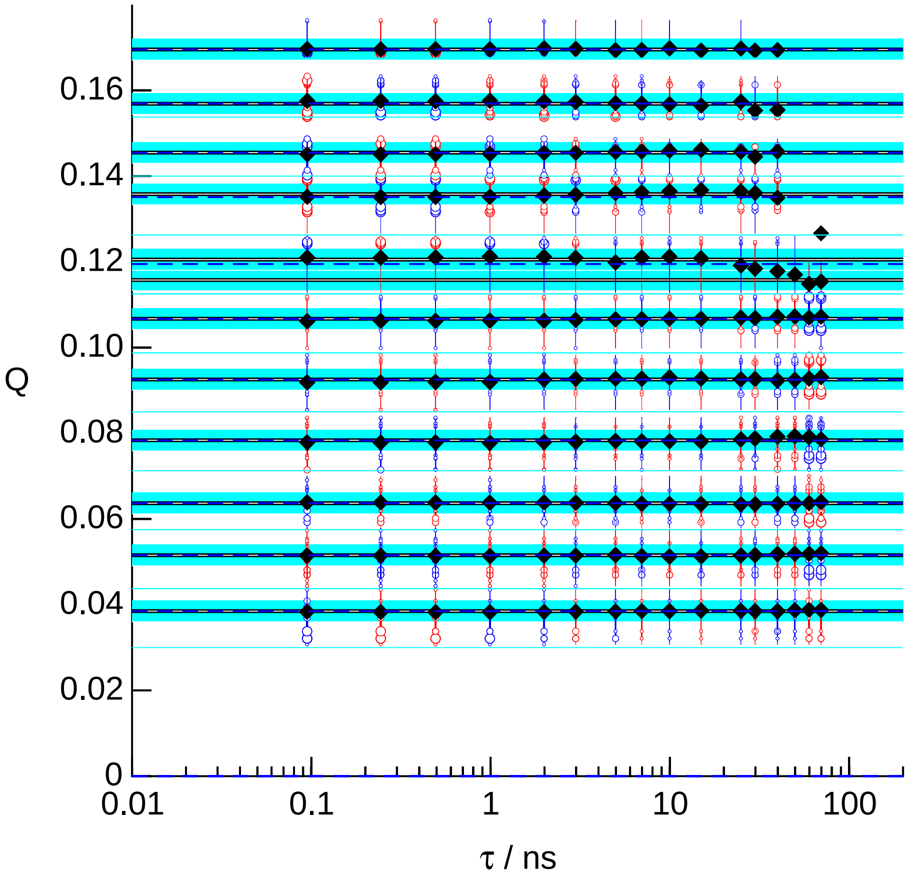

Figure 23: Mapping of single pixel contributions to $S(q,t)$ points: black diamonds. Dashed lines indicate the average q values in the q-binning zones. Size of star rays end symbols (circles) indicate the weight of the contribution from the end symbol (q,t). Cyan stripes indicate the catch zone for postcollection used in order to yield curves with consistent q-labels (controlled by parameter *r.postcoll_qcat*.

All underlying data of this plot group are preset as ASCII tables in file report20670_707.tex The corresponding sections start with a comment line: %%+++DRSPINE-PLOT:5: ...
Data in datreat format are copied to: report20670_707.6.dtr .

SDS5wD2O_"SDS_with_NaCl_in_D2O [18, 800]
Without background subtraction

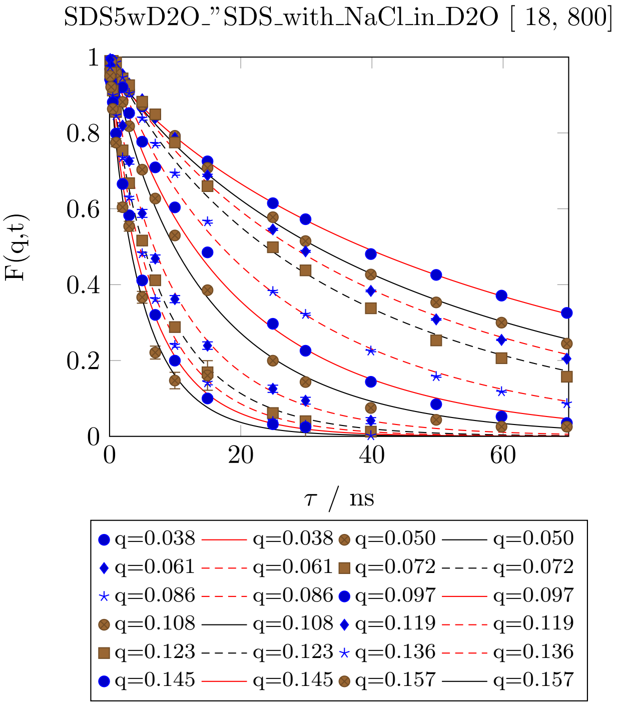

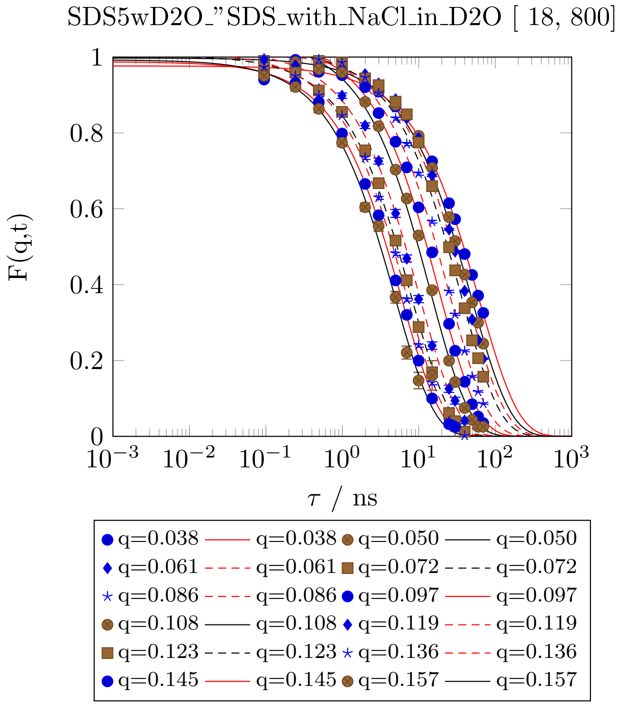

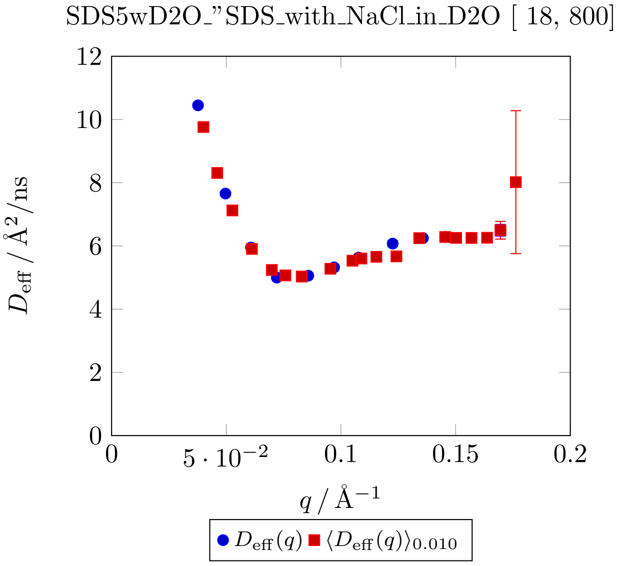

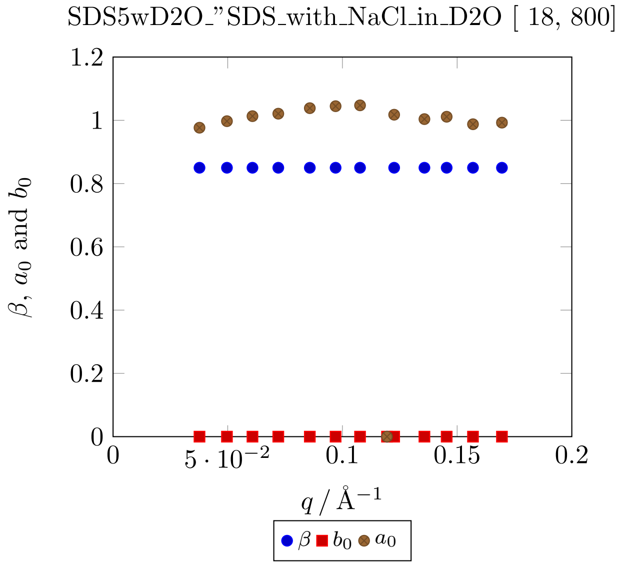

Fitting model:

$$F(q,t) = a_0 \ \left[\exp\{-(t/\tau)^{\beta}\}\right] + b_0 \tag{13}$$

Expression for the effective diffusion constant:

$$D_{\mathrm{eff}}(q) \ = \ \frac{1}{(\tau_0/\beta)\Gamma(1/\beta)\ q^2} \tag{14}$$

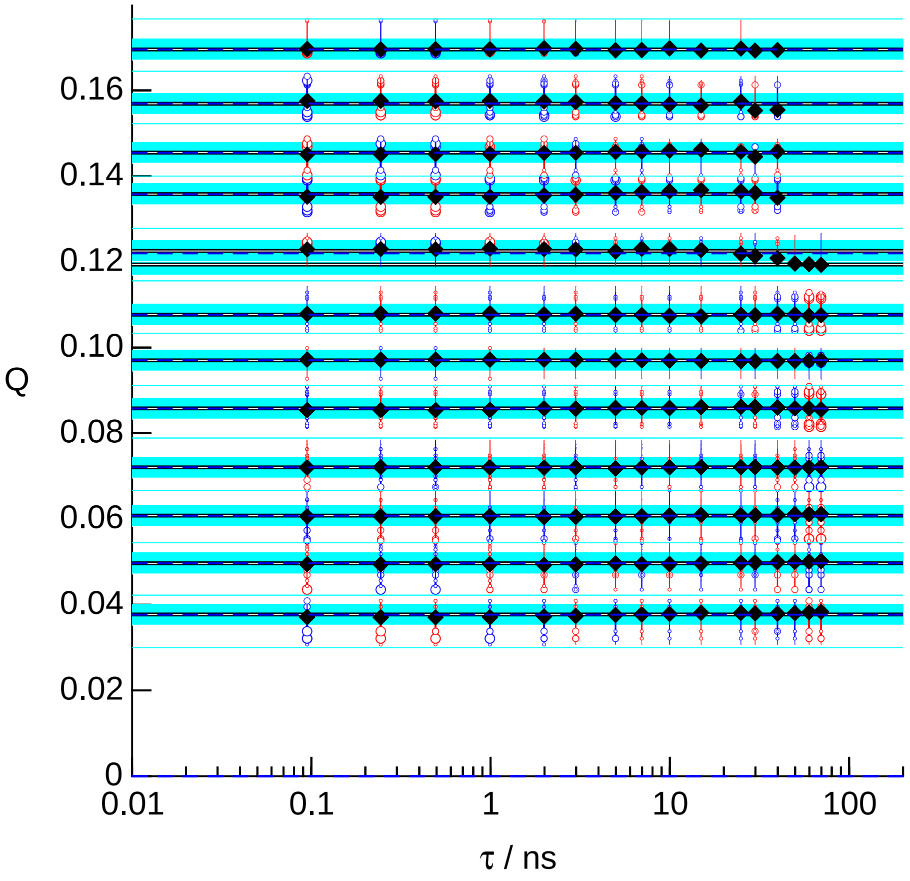

Figure 24: Mapping of single pixel contributions to $S(q,t)$ points: black diamonds. Dashed lines indicate the average q values in the q-binning zones. Size of star rays end symbols (circles) indicate the weight of the contribution from the end symbol (q,t). Cyan stripes indicate the catch zone for postcollection used in order to yield curves with consistent q-labels (controlled by parameter *r.postcoll_qcat*.

All underlying data of this plot group are preset as ASCII tables in file report20670_707.tex The corresponding sections start with a comment line: %%+++DRSPINE-PLOT:6: ...
Data in datreat format are copied to: report20670_707.7.dtr .

SDS5wD2O_"SDS_with_NaCl_in_D2O [20, 800]
Without background subtraction

SDS5wD2O_"SDS_with_NaCl_in_D2O [20, 800]

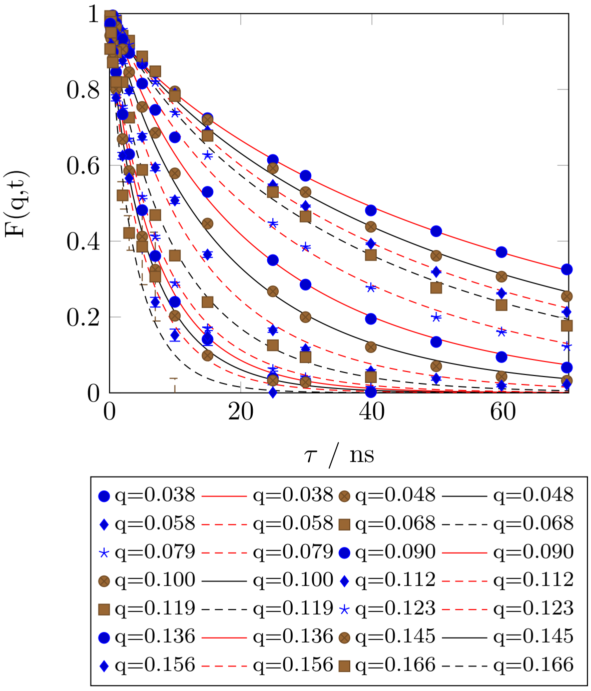

SDS5wD2O_"SDS_with_NaCl_in_D2O [20, 800]

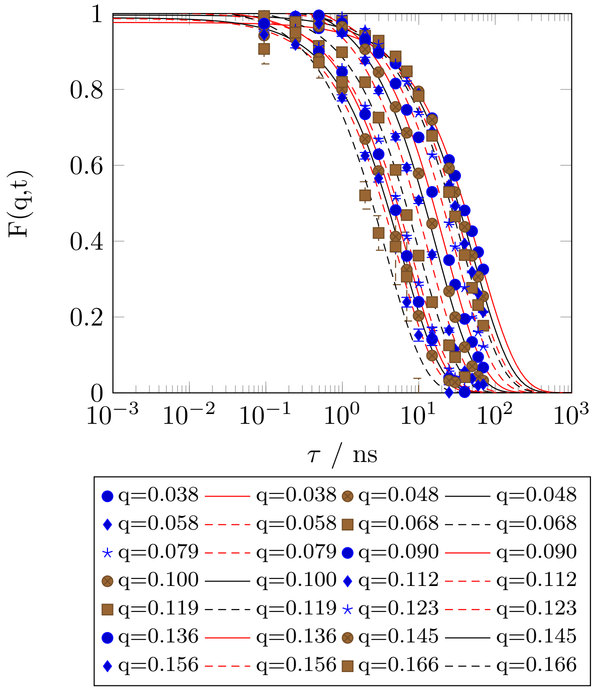

SDS5wD2O_"SDS_with_NaCl_in_D2O [20, 800]

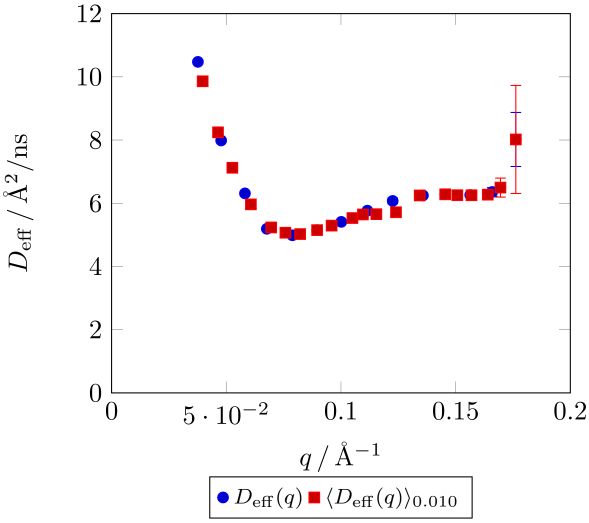

SDS5wD2O_"SDS_with_NaCl_in_D2O [20, 800]

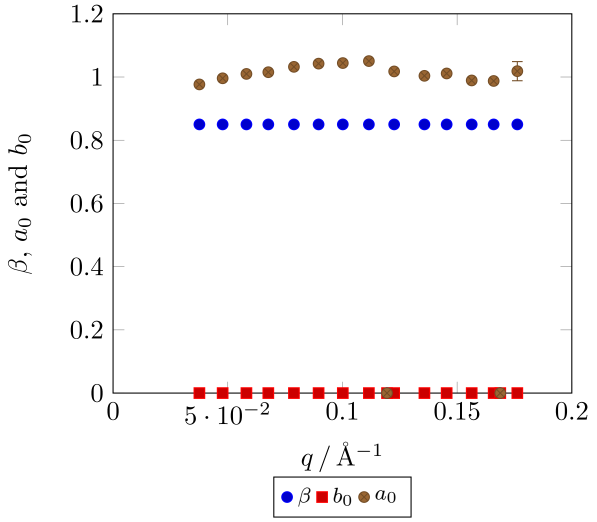

Fitting model:

$$F(q,t) = a_0 \, \left[\exp\{-(t/\tau)^{\beta}\}\right] + b_0 \tag{15}$$

Expression for the effective diffusion constant:

$$D_{\mathrm{eff}}(q) \;=\; \frac{1}{(\tau_0/\beta)\Gamma(1/\beta)\; q^2} \tag{16}$$

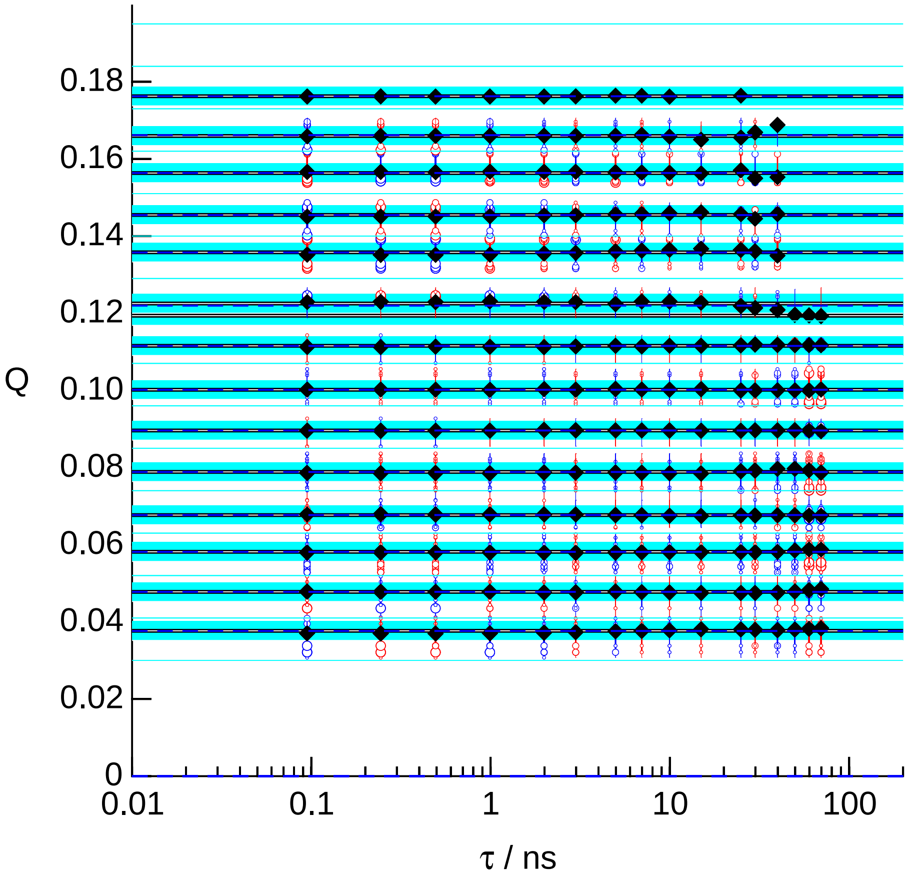

Figure 25: Mapping of single pixel contributions to $S(q,t)$ points: black diamonds. Dashed lines indicate the average q values in the q-binning zones. Size of star rays end symbols (circles) indicate the weight of the contribution from the end symbol (q,t). Cyan stripes indicate the catch zone for postcollection used in order to yield curves with consistent q-labels (controlled by parameter *r.postcoll_qcat*.

All underlying data of this plot group are preset as ASCII tables in file report20670_707.tex The corresponding sections start with a comment line: %%+++DRSPINE-PLOT:7: ...
Data in datreat format are copied to: report20670_707.8.dtr .

SDS5wD2O_"SDS_with_NaCl_in_D2O [22, 800]
Without background subtraction

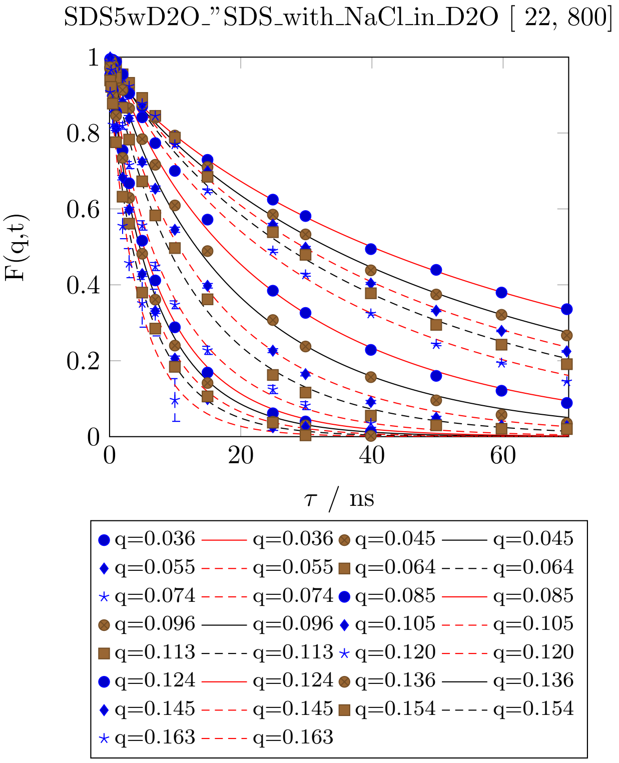

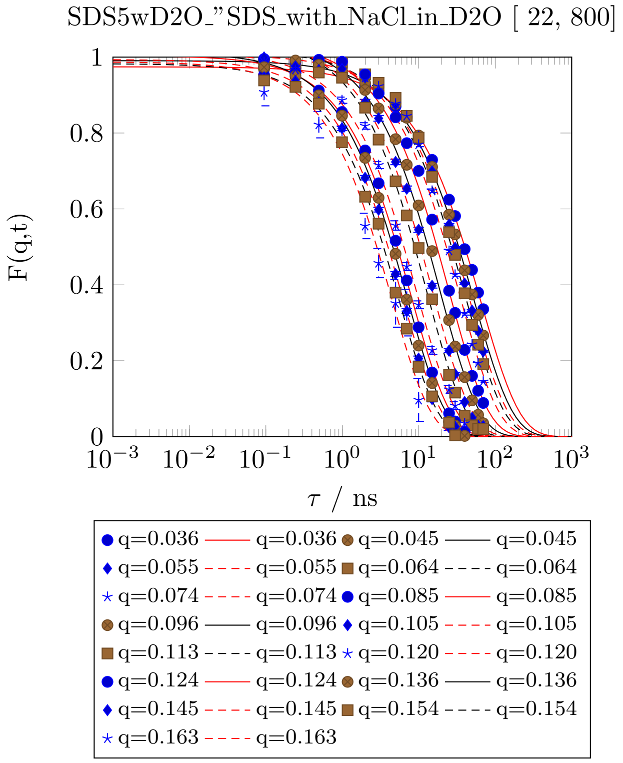

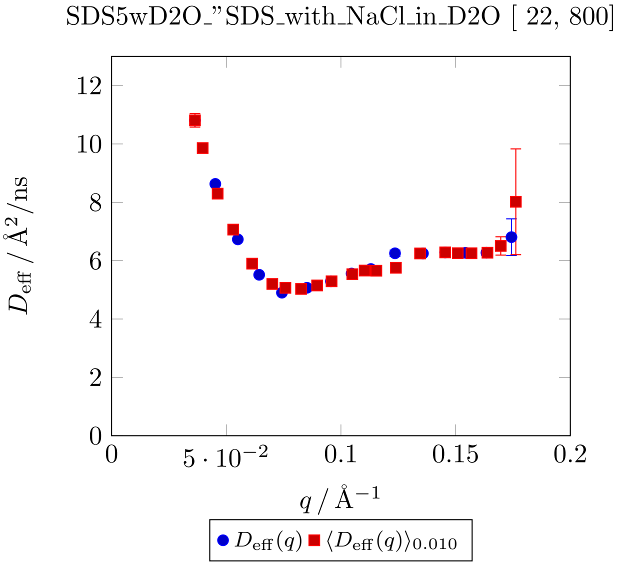

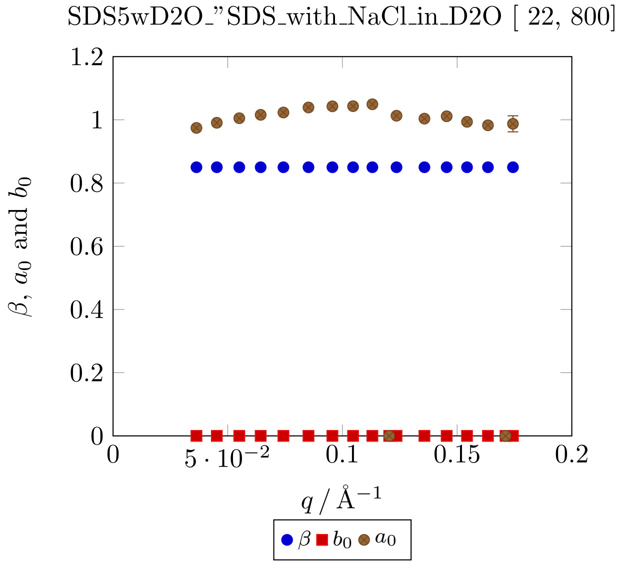

Fitting model:

$$F(q,t) = a_0 \ \left[\exp\{-(t/\tau)^{\beta}\}\right] + b_0 \tag{17}$$

Expression for the effective diffusion constant:

$$D_{\text{eff}}(q) \ = \ \frac{1}{(\tau_0/\beta)\Gamma(1/\beta)\ q^2} \tag{18}$$

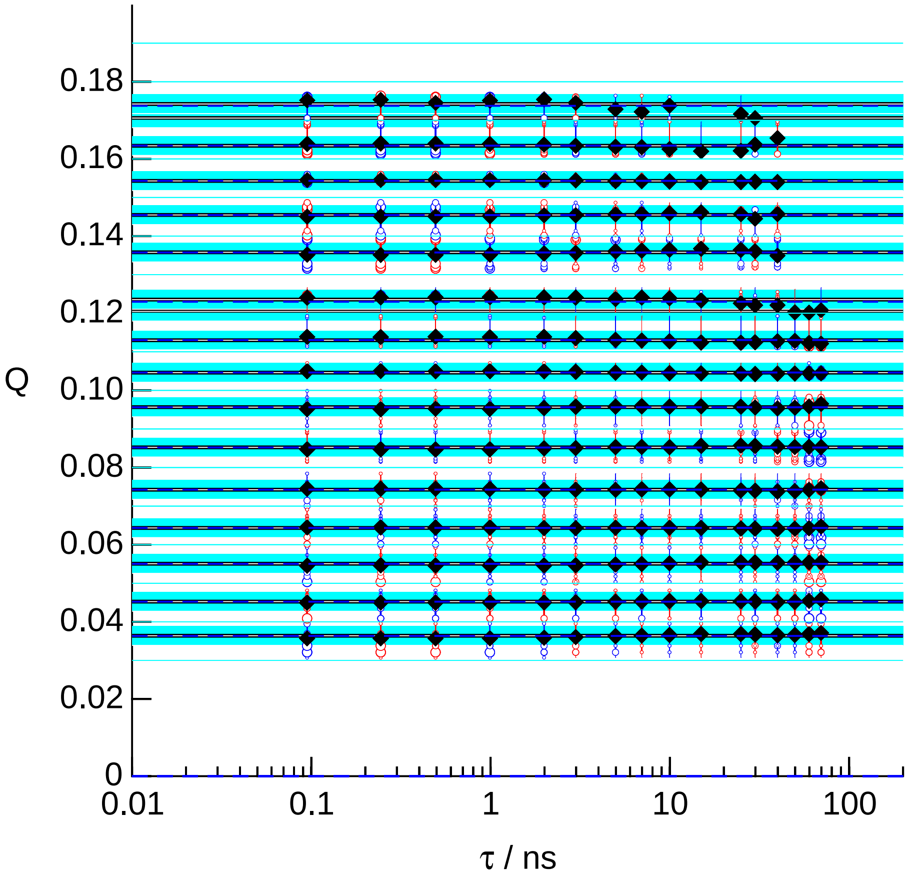

Figure 26: Mapping of single pixel contributions to $S(q,t)$ points: black diamonds. Dashed lines indicate the average q values in the q-binning zones. Size of star rays end symbols (circles) indicate the weight of the contribution from the end symbol (q,t). Cyan stripes indicate the catch zone for postcollection used in order to yield curves with consistent q-labels (controlled by parameter *r.postcoll_qcat*.

All underlying data of this plot group are preset as ASCII tables in file report20670_707.tex The corresponding sections start with a comment line: %%+++DRSPINE-PLOT:8: ...
Data in datreat format are copied to: report20670_707.9.dtr .

SDS5wD2O_"SDS_with_NaCl_in_D2O [24, 800]
Without background subtraction

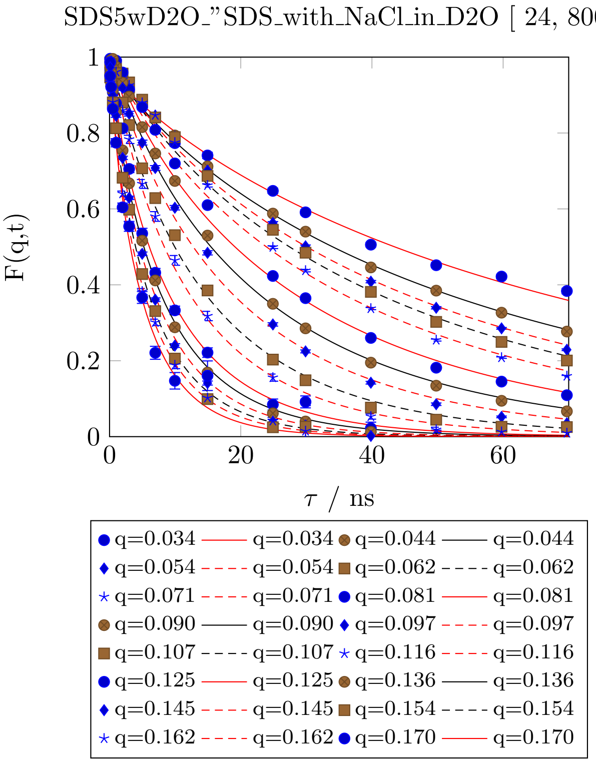

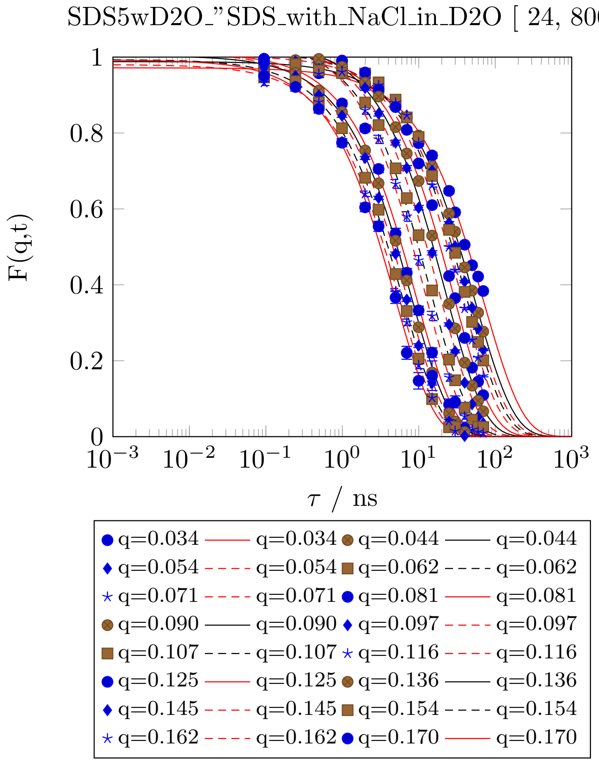

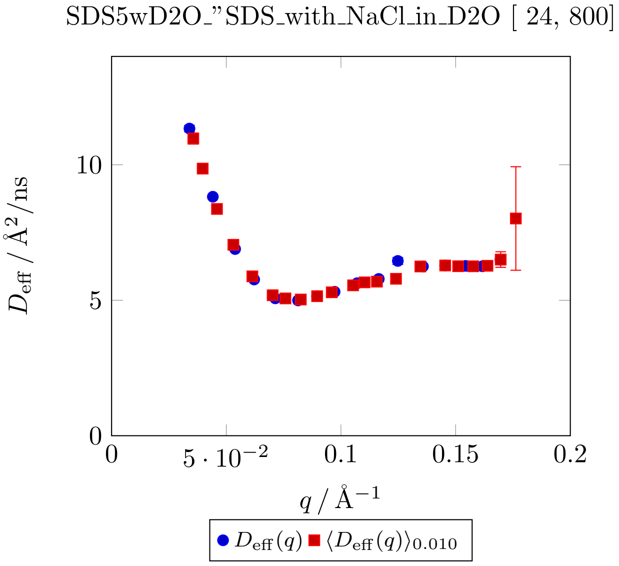

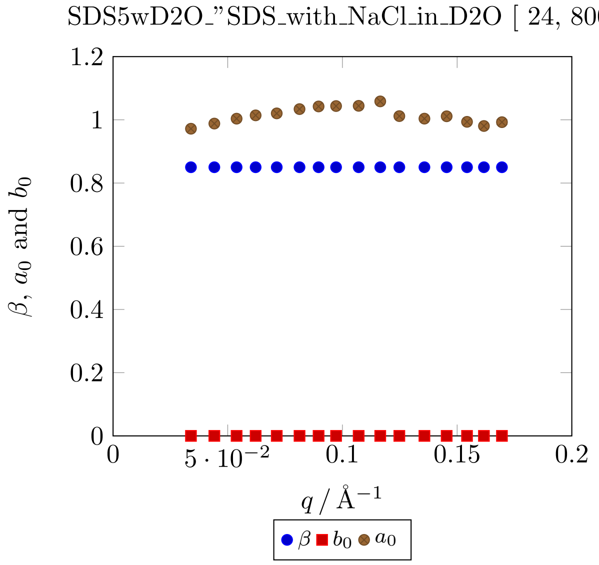

Fitting model:

$$F(q,t) = a_0\ \left[\exp\{-(t/\tau)^{\beta}\}\right] + b_0 \tag{19}$$

Expression for the effective diffusion constant:

$$D_{\mathrm{eff}}(q)\ =\ \frac{1}{(\tau_0/\beta)\Gamma(1/\beta)\ q^2} \tag{20}$$

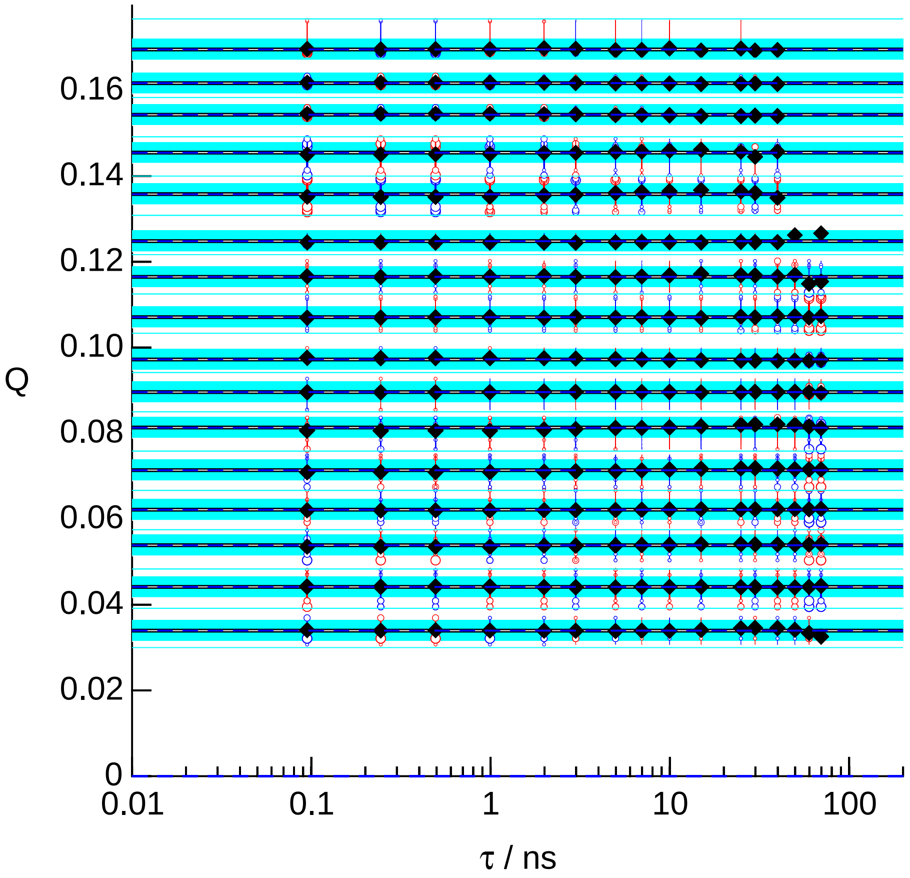

Figure 27: Mapping of single pixel contributions to $S(q,t)$ points: black diamonds. Dashed lines indicate the average q values in the q-binning zones. Size of star rays end symbols (circles) indicate the weight of the contribution from the end symbol (q,t). Cyan stripes indicate the catch zone for postcollection used in order to yield curves with consistent q-labels (controlled by parameter *r.postcoll_qcat*.

All underlying data of this plot group are preset as ASCII tables in file report20670_707.tex The corresponding sections start with a comment line: %%+++DRSPINE-PLOT:9: ...
Data in datreat format are copied to: report20670_707.10.dtr .

SDS5wD2O_"SDS_with_NaCl_in_D2O [26, 800]
Without background subtraction

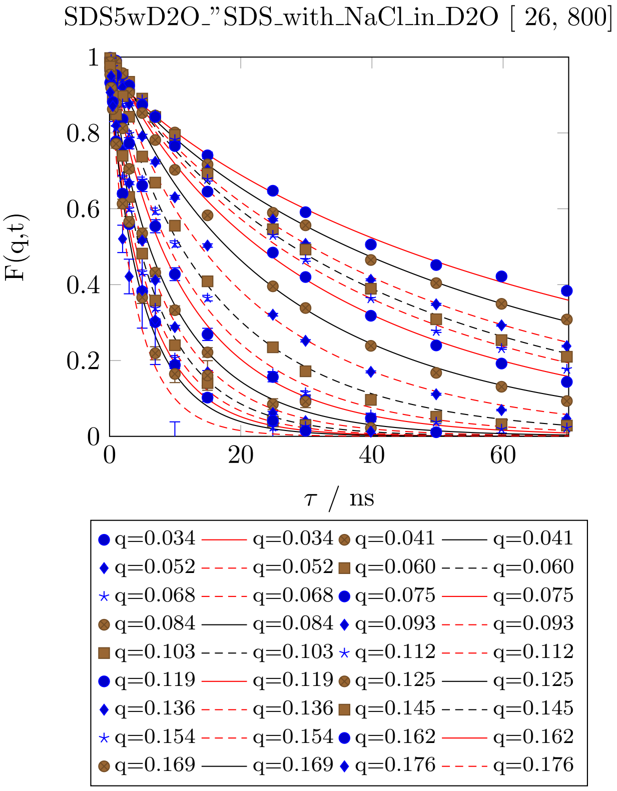

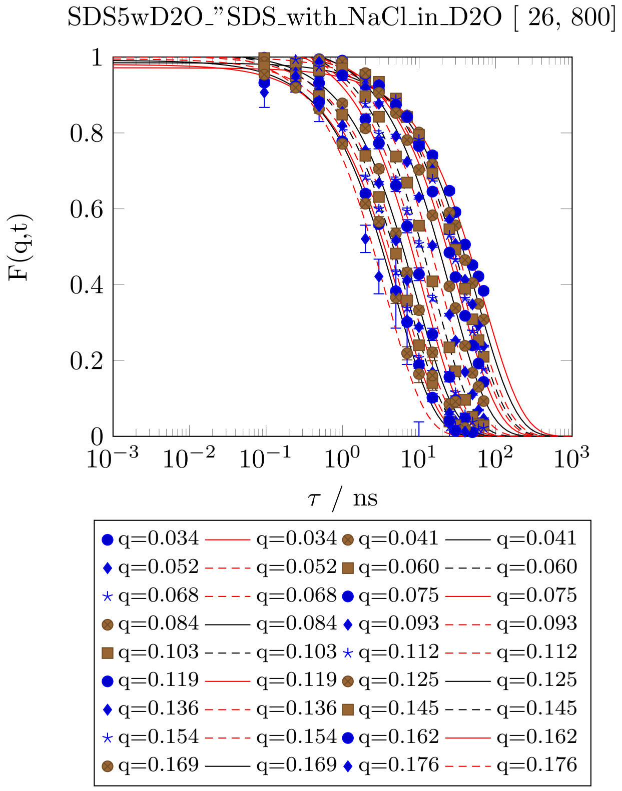

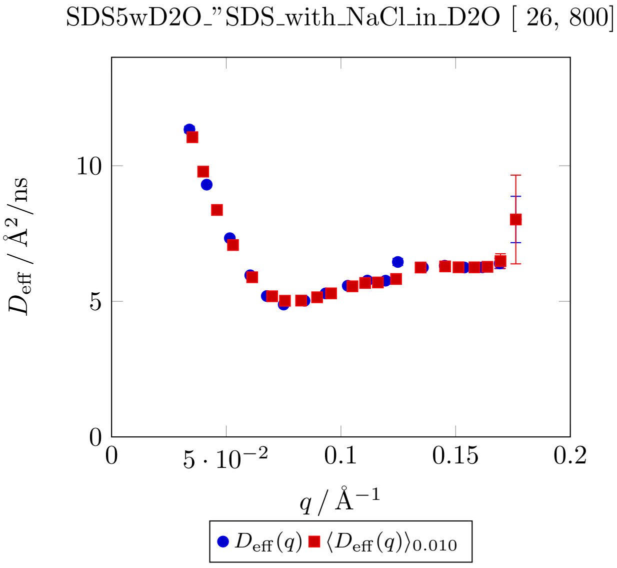

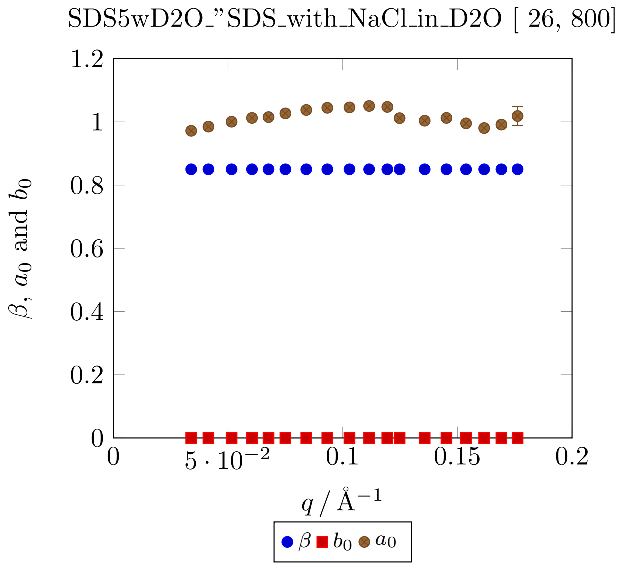

Fitting model:

$$F(q,t) = a_0 \left[\exp\{-(t/\tau)^{\beta}\}\right] + b_0 \tag{21}$$

Expression for the effective diffusion constant:

$$D_{\mathrm{eff}}(q) = \frac{1}{(\tau_0/\beta)\Gamma(1/\beta)\, q^2} \tag{22}$$

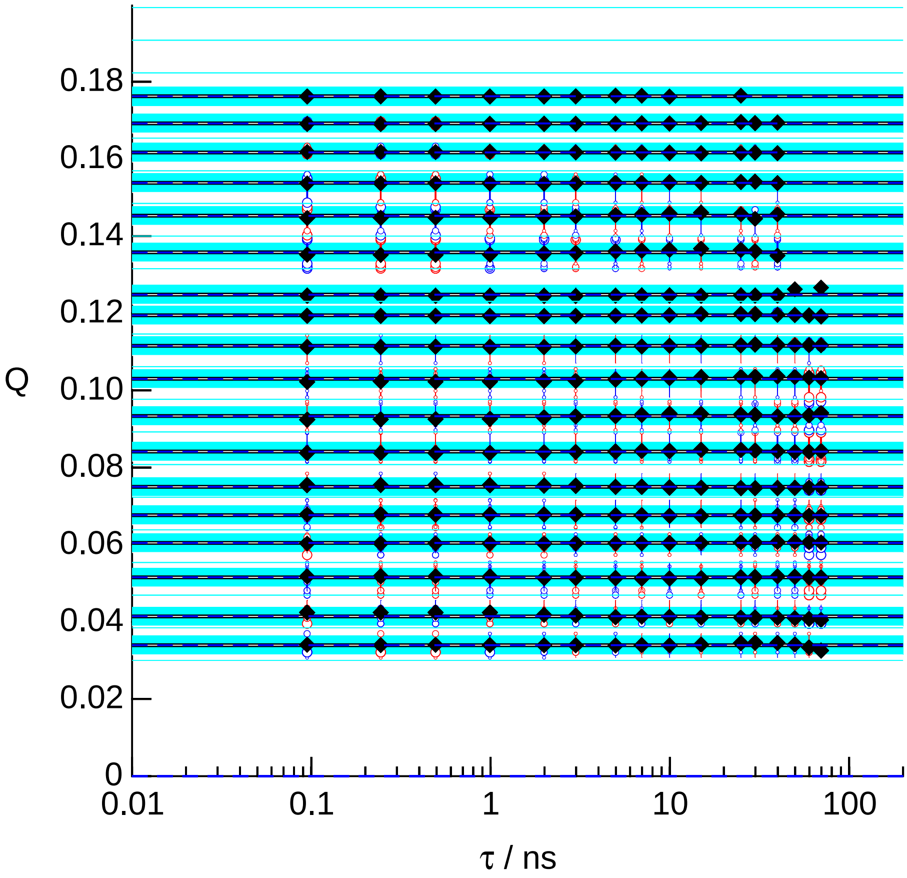

Figure 28: Mapping of single pixel contributions to $S(q,t)$ points: black diamonds. Dashed lines indicate the average q values in the q-binning zones. Size of star rays end symbols (circles) indicate the weight of the contribution from the end symbol (q,t). Cyan stripes indicate the catch zone for postcollection used in order to yield curves with consistent q-labels (controlled by parameter *r.postcoll_qcat*.

All underlying data of this plot group are preset as ASCII tables in file report20670_707.tex The corresponding sections start with a comment line: %%+++DRSPINE-PLOT:10: ...
Data in datreat format are copied to: report20670_707.11.dtr .

SDS5wD2O_"SDS_with_NaCl_in_D2O [30, 800]
Without background subtraction

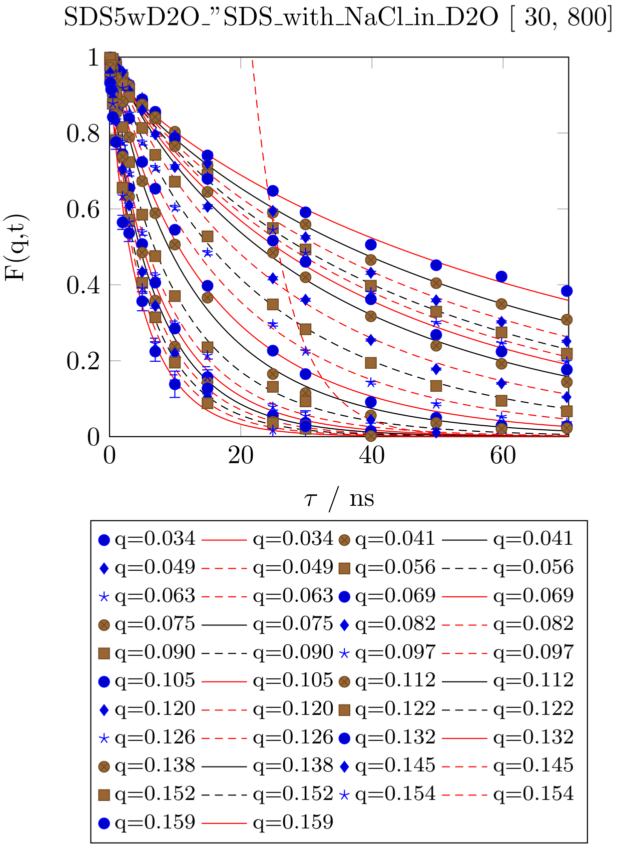

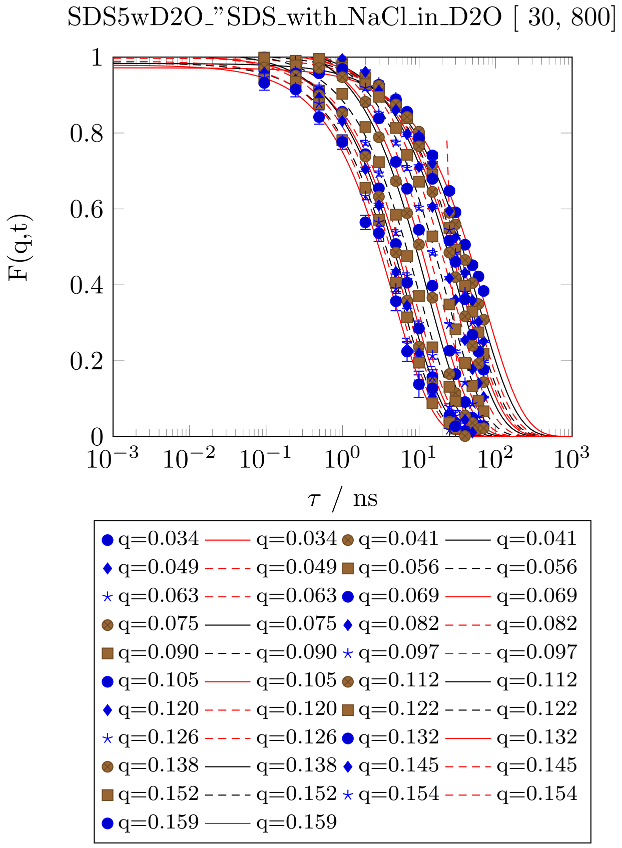

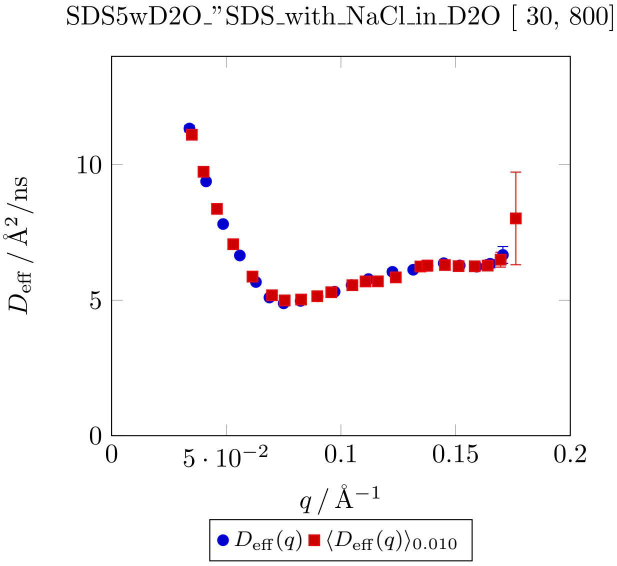

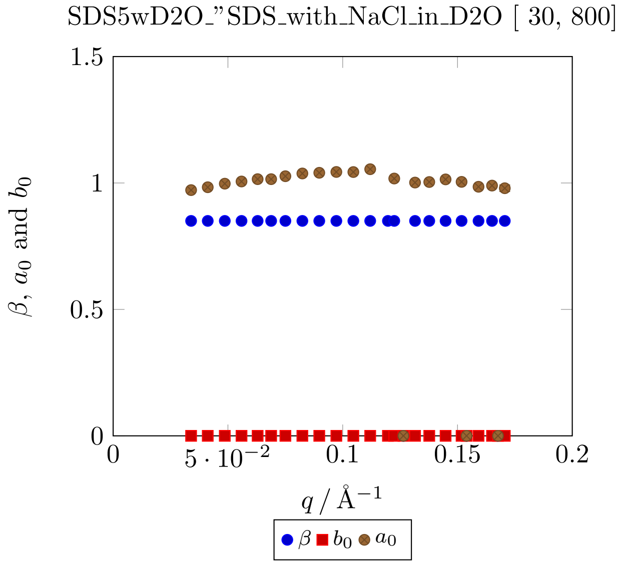

Fitting model:

$$F(q,t) = a_0 \left[\exp\{-(t/\tau)^{\beta}\}\right] + b_0 \tag{23}$$

Expression for the effective diffusion constant:

$$D_{\rm eff}(q) = \frac{1}{(\tau_0/\beta)\Gamma(1/\beta)\, q^2} \tag{24}$$

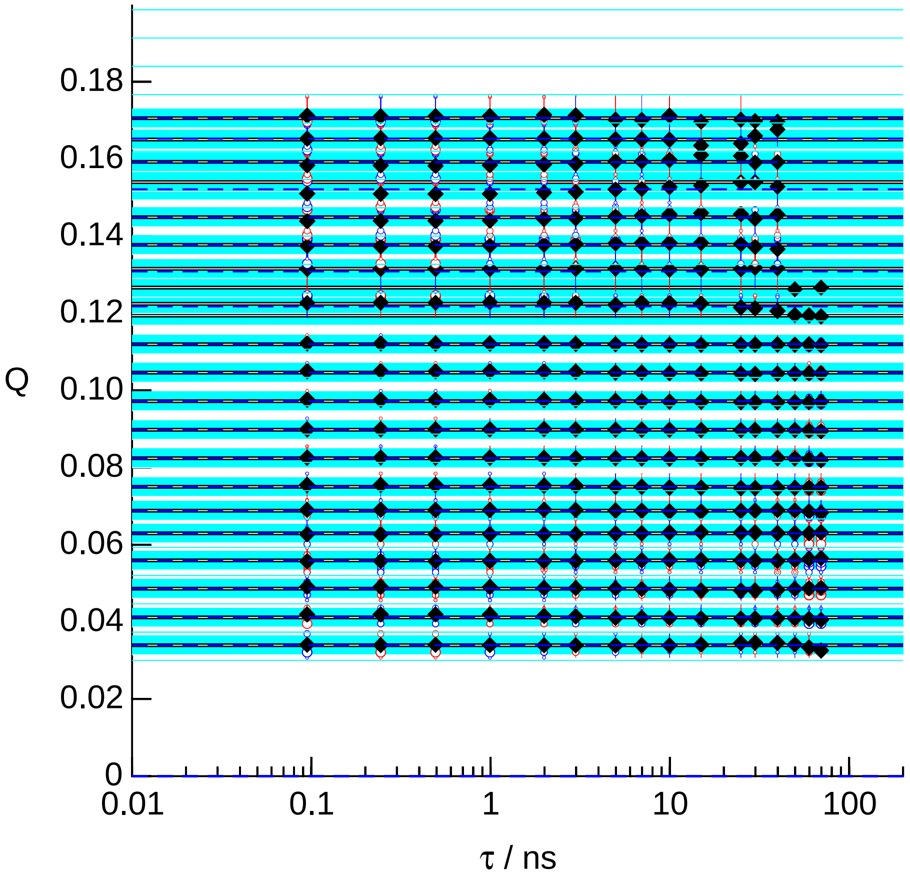

Figure 29: Mapping of single pixel contributions to $S(q,t)$ points: black diamonds. Dashed lines indicate the average q values in the q-binning zones. Size of star rays end symbols (circles) indicate the weight of the contribution from the end symbol (q,t). Cyan stripes indicate the catch zone for postcollection used in order to yield curves with consistent q-labels (controlled by parameter *r.postcoll_qcat*.

All underlying data of this plot group are preset as ASCII tables in file report20670_707.tex The corresponding sections start with a comment line: %%+++DRSPINE-PLOT:11: ...
Data in datreat format are copied to: report20670_707.12.dtr .

SDS5wD2O_"SDS_with_NaCl_in_D2O [32, 800]
Without background subtraction

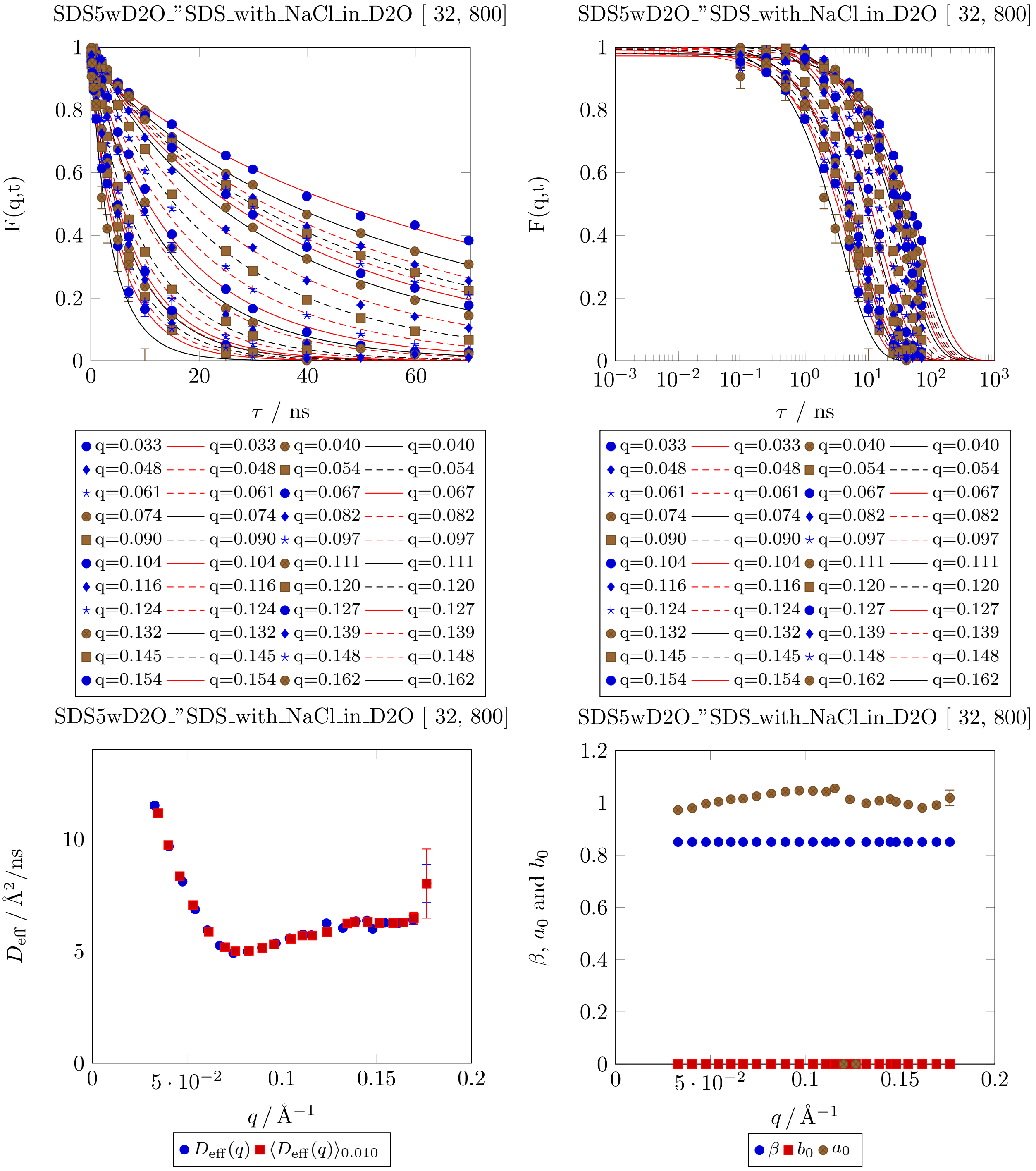

Fitting model:

$$F(q,t) = a_0 \left[\exp\{-(t/\tau)^{\beta}\}\right] + b_0 \tag{25}$$

Expression for the effective diffusion constant:

$$D_{\mathrm{eff}}(q) = \frac{1}{(\tau_0/\beta)\Gamma(1/\beta)\, q^2} \tag{26}$$

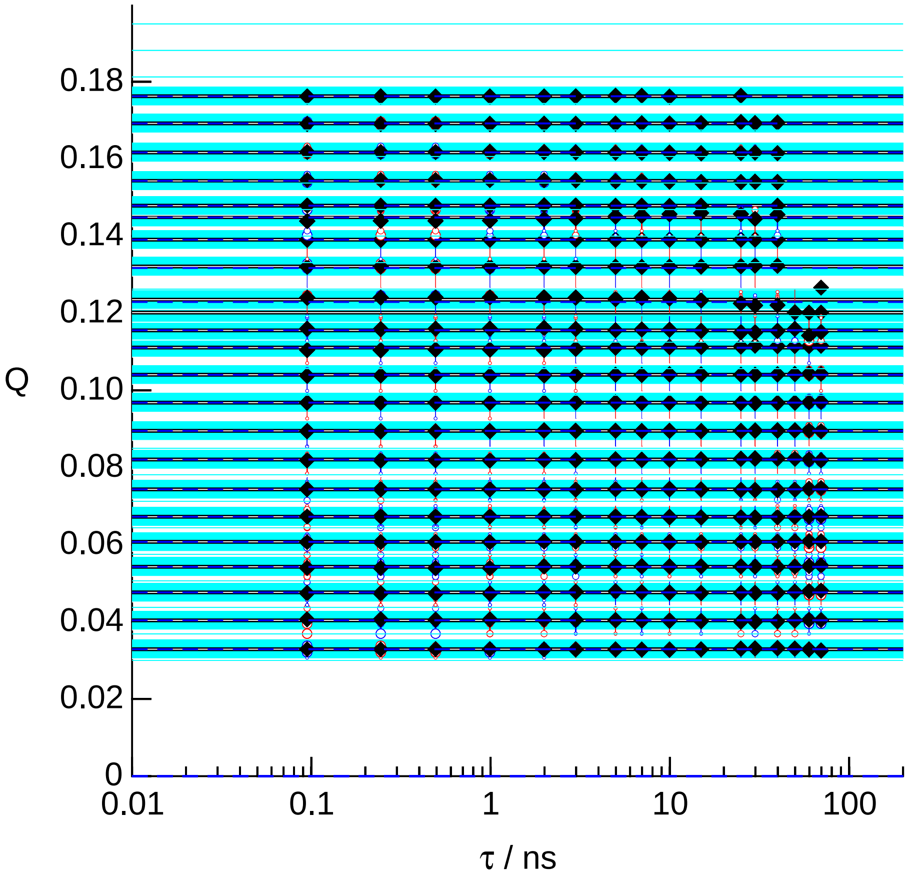

Figure 30: Mapping of single pixel contributions to $S(q,t)$ points: black diamonds. Dashed lines indicate the average q values in the q-binning zones. Size of star rays end symbols (circles) indicate the weight of the contribution from the end symbol (q,t). Cyan stripes indicate the catch zone for postcollection used in order to yield curves with consistent q-labels (controlled by parameter *r.postcoll_qcat*.

All underlying data of this plot group are preset as ASCII tables in file report20670_707.tex The corresponding sections start with a comment line: %%+++DRSPINE-PLOT:12: ...
Data in datreat format are copied to: report20670_707.13.dtr .

SDS5wD2O_"SDS_with_NaCl_in_D2O [34, 800]
Without background subtraction

SDS5wD2O_"SDS_with_NaCl_in_D2O [34, 800]

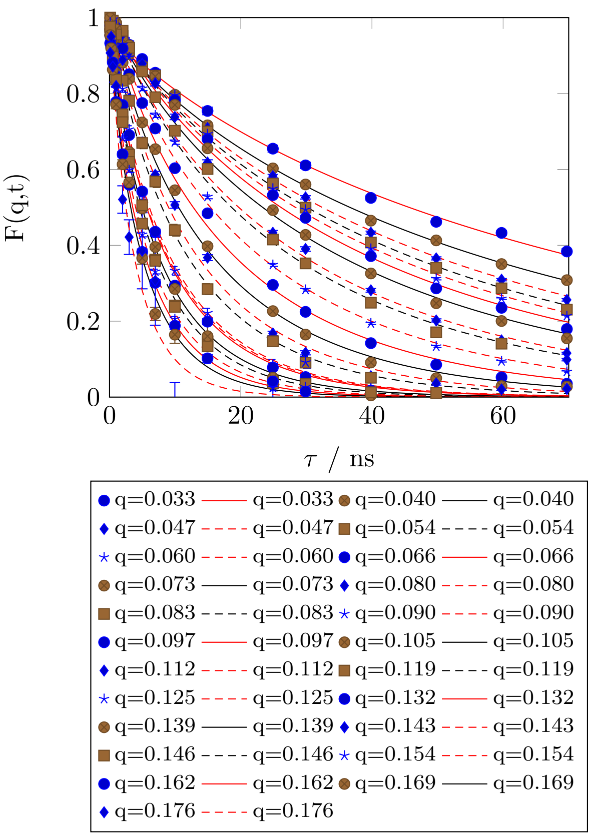

SDS5wD2O_"SDS_with_NaCl_in_D2O [34, 800]

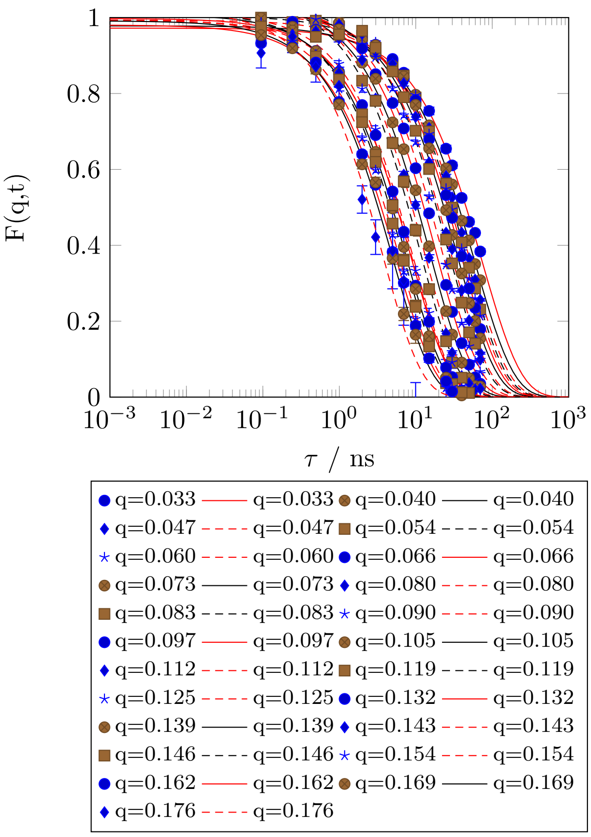

SDS5wD2O_"SDS_with_NaCl_in_D2O [34, 800]

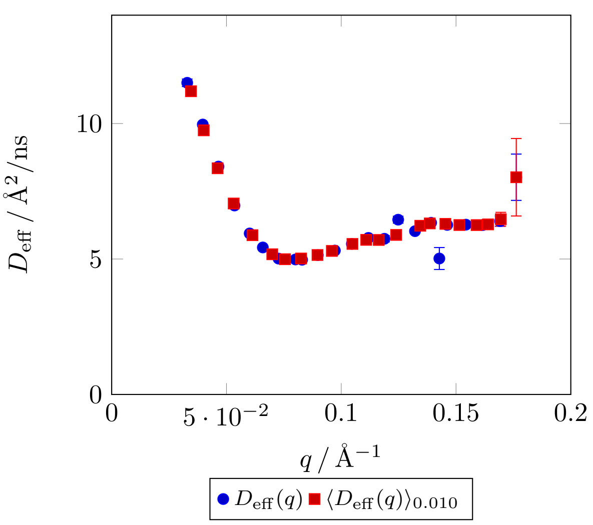

SDS5wD2O_"SDS_with_NaCl_in_D2O [34, 800]

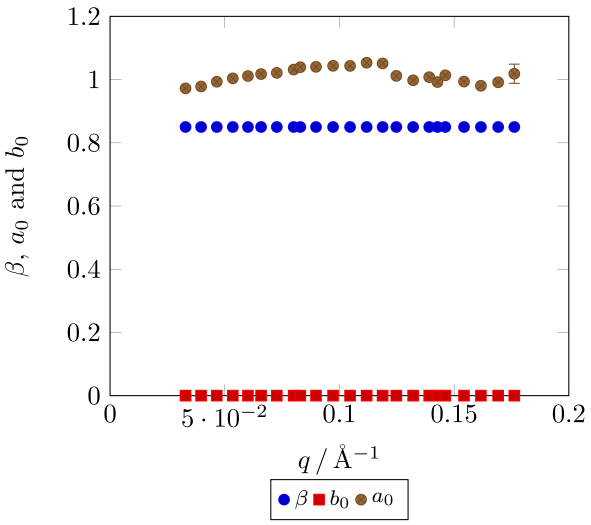

Fitting model:

$$F(q,t) = a_0 \left[\exp\{-(t/\tau)^{\beta}\}\right] + b_0 \tag{27}$$

Expression for the effective diffusion constant:

$$D_{\mathrm{eff}}(q) = \frac{1}{(\tau_0/\beta)\Gamma(1/\beta)\, q^2} \tag{28}$$

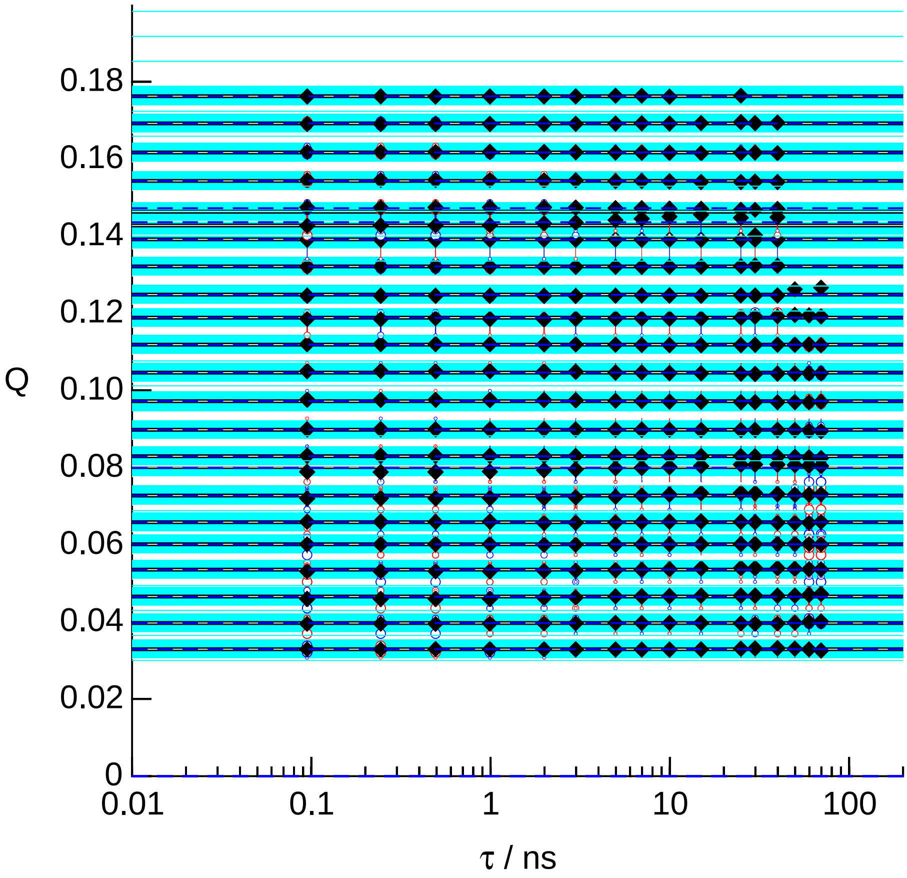

Figure 31: Mapping of single pixel contributions to $S(q,t)$ points: black diamonds. Dashed lines indicate the average q values in the q-binning zones. Size of star rays end symbols (circles) indicate the weight of the contribution from the end symbol (q,t). Cyan stripes indicate the catch zone for postcollection used in order to yield curves with consistent q-labels (controlled by parameter *r.postcoll_qcat*.

All underlying data of this plot group are preset as ASCII tables in file report20670_707.tex The corresponding sections start with a comment line: %%+++DRSPINE-PLOT:13: ...
Data in datreat format are copied to: report20670_707.14.dtr .

SDS5wD2O_"SDS_with_NaCl_in_D2O [36, 800]
Without background subtraction

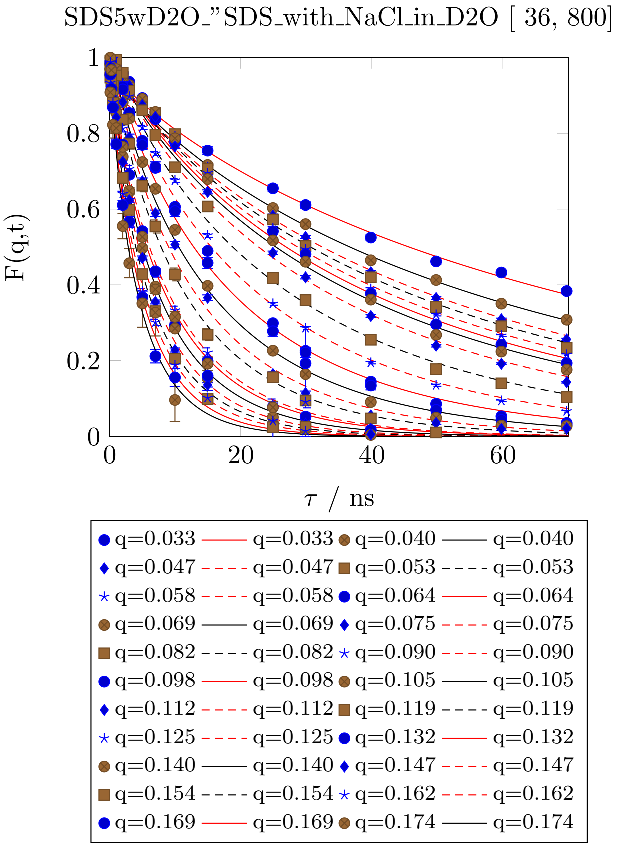

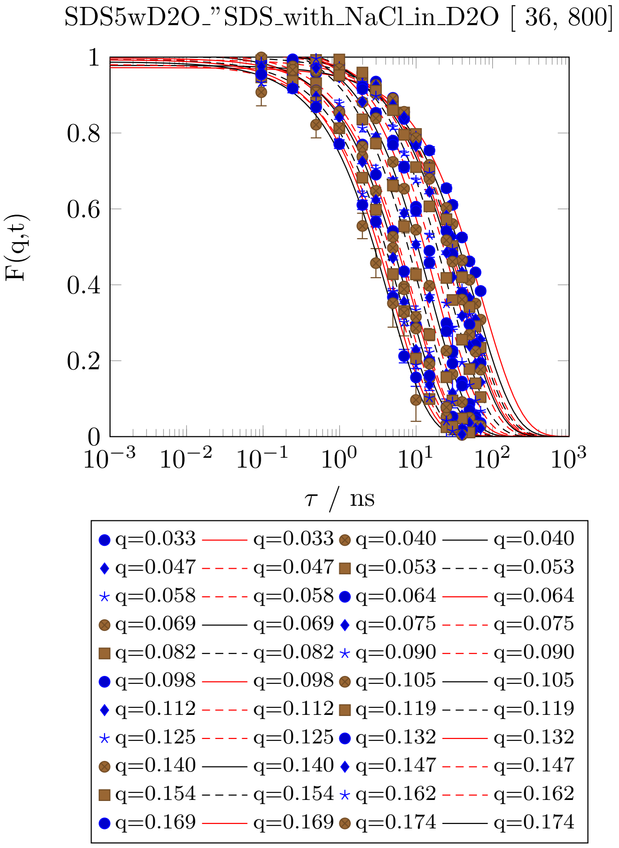

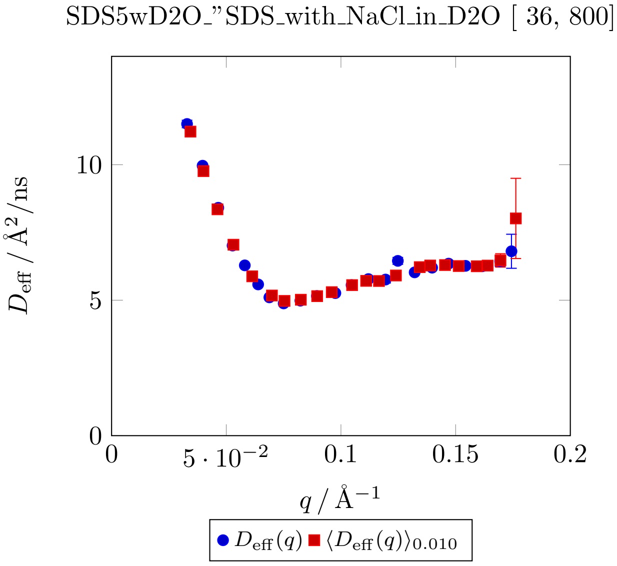

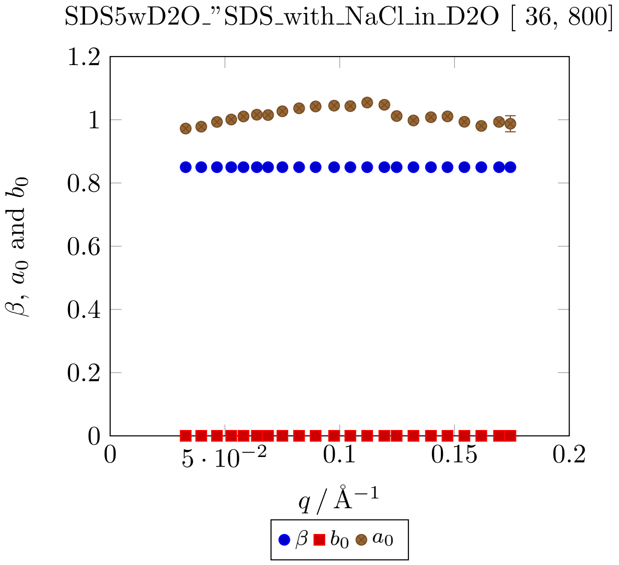

Fitting model:

$$F(q,t) = a_0 \left[\exp\{-(t/\tau)^{\beta}\}\right] + b_0 \tag{29}$$

Expression for the effective diffusion constant:

$$D_{\mathrm{eff}}(q) = \frac{1}{(\tau_0/\beta)\Gamma(1/\beta)\, q^2} \tag{30}$$

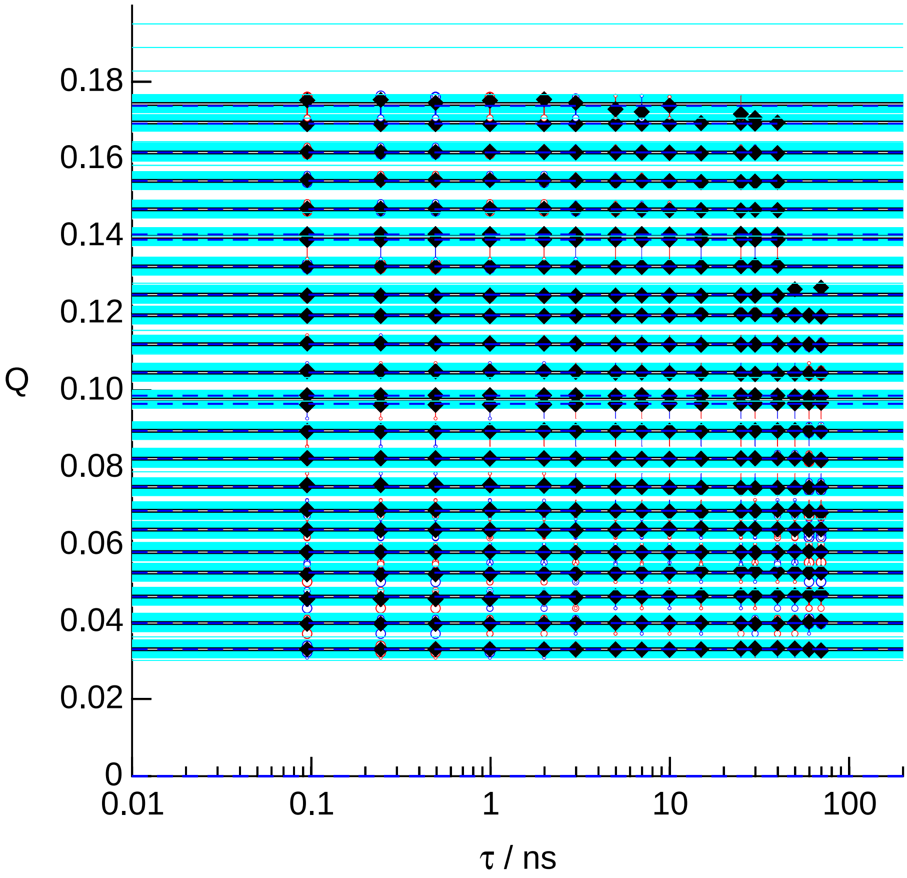

Figure 32: Mapping of single pixel contributions to $S(q,t)$ points: black diamonds. Dashed lines indicate the average q values in the q-binning zones. Size of star rays end symbols (circles) indicate the weight of the contribution from the end symbol (q,t). Cyan stripes indicate the catch zone for postcollection used in order to yield curves with consistent q-labels (controlled by parameter *r.postcoll_qcat*.

All underlying data of this plot group are preset as ASCII tables in file report20670_707.tex The corresponding sections start with a comment line: %%+++DRSPINE-PLOT:14: ...
Data in datreat format are copied to: report20670_707.15.dtr .

Consolidated D_{eff} [$Å^2$/ns] of all evaluation variants (q-catch = 0.0100)

q	Deff	Error(Deff)
0.034391	11.223007	0.167066
0.040064	9.764242	0.068115
0.046175	8.354549	0.049222
0.053127	7.047848	0.027779
0.061324	5.880940	0.019613
0.069916	5.172527	0.019917
0.075401	4.973699	0.036969
0.082509	5.016945	0.018727
0.089633	5.149308	0.038132
0.095990	5.296648	0.027169
0.104864	5.556490	0.045656
0.110993	5.713891	0.071990
0.116582	5.708161	0.140037
0.123972	5.911729	0.098616
0.134355	6.220042	0.052083
0.138914	6.280648	0.116270
0.145369	6.296748	0.071616
0.151339	6.260067	0.054831
0.159186	6.252230	0.108996
0.163958	6.277693	0.160739
0.169552	6.468785	0.246960
0.176244	8.018276	1.480188

Macro: makroSDS

```
makro
clear all
verbose info
version
! ==== set paths ====
datapath ../nse_data
savepath ./
path
eshape triangular
! ==== set some control parameters (otherwise defaults are in effect) ====
set r.min_counts    100
set r.min_sigma     0.05
set r.ave_min       0.0
set r.ave_maxdev    0.25
set r.fqt_min       0.2
set r.fqt_maxsigma  0.8
set r.phase_tol     1e-9
set r.phase_maxdev  1.27
set r.phase_minoff  0.0001
set r.phase_cstep   1.57
set r.max_chisq     1000
set r.postcoll_qcat 0.0025e10
! ==== set binning
bins  pix nbins  8      ! possible choices 4 8 16 32
bins  tof custom 1 2
! read data:
! read <rawfile>  as resolution|sample|background [transmission <value> [check all|critical --
!
read m20666.echo  as resolution
read m20668.echo  as resolution
read m20669.echo  as resolution
read m20676.echo  as resolution
read m20670.echo  as sample
read m20672.echo  as sample
read m20673.echo  as sample
read m20674.echo  as sample
read m20675.echo  as sample
dir
!
match all           ! associate reference (end backround) runs with sample runs
fit res             ! fit the resolution only
fit sam flag offset ! get the phase offset and fit sample
! set some variables for later use
set bgr 0
set tf  1
set beta_ave 0.85
! === post processiong and output ========================================================
report start
report dir details more    normalize max_bvar 0 max_bdiff 0
!     show details
!                          and all phasescans irrespective of
!                          magnetic fluctuations in the environment
!
! set up the collection binning scheme
! for the reactor instrument NSE
! the matching of chosen taus into proper corresponding bins is enforced by nbins LARGE
histo tau nbins 800  min 0.02E-9 max 400E-9 log
! by default create an number of different q-binnig granularity for a posteriorfi choice
! respectively comparison
 histo q   nbins 6   min 0.030E10 max 0.25E10
 collect bgr (bgr) tfac (tf)
 report sqtplot fix_beta (beta_ave)
 histo q   nbins 8   min 0.030E10 max 0.25E10
 collect bgr (bgr) tfac (tf)
 report sqtplot fix_beta (beta_ave)
 histo q   nbins 10   min 0.030E10 max 0.25E10
 collect bgr (bgr) tfac (tf)
 report sqtplot fix_beta (beta_ave)
 histo q   nbins 12   min 0.030E10 max 0.25E10
 collect bgr (bgr) tfac (tf)
 report sqtplot fix_beta (beta_ave)
 histo q   nbins 14   min 0.030E10 max 0.25E10
 collect bgr (bgr) tfac (tf)
 report sqtplot fix_beta (beta_ave)
 histo q   nbins 16   min 0.030E10 max 0.25E10
 collect bgr (bgr) tfac (tf)
 report sqtplot fix_beta (beta_ave)
 histo q   nbins 18   min 0.030E10 max 0.25E10
 collect bgr (bgr) tfac (tf)
 report sqtplot fix_beta (beta_ave)
 histo q   nbins 20   min 0.030E10 max 0.25E10
 collect bgr (bgr) tfac (tf)
 report sqtplot fix_beta (beta_ave)
 histo q   nbins 22   min 0.030E10 max 0.25E10
 collect bgr (bgr) tfac (tf)
 report sqtplot fix_beta (beta_ave)
 histo q   nbins 24   min 0.030E10 max 0.25E10
 collect bgr (bgr) tfac (tf)
 report sqtplot fix_beta (beta_ave)
 histo q   nbins 26   min 0.030E10 max 0.25E10
 collect bgr (bgr) tfac (tf)
 report sqtplot fix_beta (beta_ave)
 histo q   nbins 30   min 0.030E10 max 0.25E10
 collect bgr (bgr) tfac (tf)
 report sqtplot  fix_beta (beta_ave)
 histo q   nbins 32   min 0.030E10 max 0.25E10
 collect bgr (bgr) tfac (tf)
 report sqtplot  fix_beta (beta_ave)
 histo q   nbins 34   min 0.030E10 max 0.25E10
 collect bgr (bgr) tfac (tf)
 report sqtplot  fix_beta (beta_ave)
 histo q   nbins 36   min 0.030E10 max 0.25E10
 collect bgr (bgr) tfac (tf)
 report sqtplot  fix_beta (beta_ave)
report end      ! finish tex report file and create pdf
clean_logs
quit
```
