# Supplementary material for: Efficient data extraction from neutron time-of-flight spin-echo raw data
Source: J Appl Crystallogr. 2019 Aug 29;52(Pt 5):1022–34. doi: 10.1107/S1600576719010847 (PMC6782076; doi:10.1107/S1600576719010847)
Supplement: Supplementary file 2 [file j-52-01022-sup2.pdf]

Supporting Information: Efficient data extraction from neutron time-of-flight spin-echo raw data.

P.A. Zolnierczuk,[a] O. Holderer,[b] S. Pasini,[b] T. Kozielewski,[c] L.R. Stingaciu[d] and M. Monkenbusch[c]

[a]*Forschungszentrum Jülich GmbH, JCNS Outstation, Oak Ridge, Tennessee, USA,* [b]*Forschungszentrum Jülich GmbH, JCNS MLZ, Garching , Germany,* [c]*Forschungszentrum Jülich GmbH, JCNS-1, Jülich , Germany, and* [d]*NScD, Oak Ridge National Laboratory, Oak Ridge, TN, 37831, USA*

NSE; neutron spin-echo; spallation neutron source; data reduction

1. Derivation of the NSE signal in Gaussian approximation (Eq. 3-6) in the main text.

We start by considering the main spin-precession coding variables, the field integrals in the first and second spectrometer arm J_1 and J_2 respectively.

$$J_1 = \int_{\pi/2_1}^{\text{sample}} |\mathbf{B}(l)| dl \quad \text{and} \quad J_2 = \int_{\text{sample}}^{\pi/2_2} |\mathbf{B}(l)| dl \tag{1}$$

where $\mathbf{B}$ is the magnetic field (induction) along the neutron path parametrized by the path length variable l. For simplicity we assume that the π-flipper, which reverses the effective precession sense, coincides with the sample.

The neutron velocities in both arms are v_1 and v_2, their difference corresponds to the energy transfer to the neutron by the scattering process at the sample. The spin-precession angle in each arm then is

$$\psi_i = \frac{\gamma_n J_i}{v_i} \tag{2}$$

and taking into account the action of the π-flipper the final precession angle is

$$\Psi = \psi_1 - \psi_2 \ = \frac{\gamma_n J_1}{v_1} - \frac{\gamma_n J_2}{v_2} \tag{3}$$

Application of high resolution spin-echo implies that the velocity difference Δv due to a typical energy transfer $\Delta E = \hbar\omega$ is very small, with $v_1 = v = h/(m_n\lambda)$ and $v_2 = v + \Delta v \simeq h/(m_n\lambda) + \lambda\omega/2\pi$ then inserting the resulting precession angle into the (ideal) analyzer transmission function $T = 1 \pm \cos(\pm\Psi)/2$ yields the probability of this neutron to arrive at the detector:

$$\frac{1}{2}\left[1 \pm \cos\left(-\frac{\gamma_n J_1}{(h/m_n)\lambda^{-1}} + \frac{\gamma_n J_2}{(h/m_n)\lambda^{-1} + \lambda\omega/2\pi}\right)\right] \tag{4}$$

since ω is small the second term in the argument of the cosine in Eq. 4 can be replaced by its Taylor expansion and to get the average over the probabilities of energy transfers we integrate over the spectral part of the scattering function:

$$I_{\mathrm{Det}} \propto \int \left[1 \pm \cos\left(-\frac{\gamma_n J_1}{(h/m_n)\lambda^{-1}} + \frac{\gamma_n J_2}{(h/m_n)\lambda^{-1}} + \frac{\gamma_n J_2 \lambda^3}{2\pi(h/m_n)^2}\omega\right)\right] S(Q,\omega) d\omega \tag{5}$$

where I_{Det} denotes the detector intensity for an ensemble on neutrons still with unique, sharp value of the initial velocity, c.f. wavelength λ.

For the next step we utilize the fact that $S(Q,\omega) \simeq S(Q,-\omega)$ in the typical realm of NSE experiments (i.e. $\hbar\omega << k_B T$), the cosine addition theorem and $S(Q) = \int S(Q,\omega)d\omega$:

$$I_{\mathrm{Det}} \propto \left[S(Q) \pm \cos\left(\frac{\gamma_n(J_2 - J_1)\lambda}{(h/m_n)}\right) \int \cos\left(\frac{\gamma_n J_2 \lambda^3}{2\pi(h/m_n)^2}\omega\right) S(Q,\omega)d\omega\right] \tag{6}$$

since the NSE spectrometer is operated such that the precession paths are very close to symmetry we have $J_1 \simeq J_2 = J$ and the further steps are expressed now in

terms of J the nominal field integral and the field integral asymmetry $\Delta J = J_2 - J_1$, at ideal (full) symmetry $\Delta J = 0$.

1.1. Gaussian approximation for envelope shape and resolution

In a real spectrometer it is not possible to work with one exactly defined wavelength, in fact the main reason for the applicability of spin echo is the large possible width of the incoming neutron wavelength (velocity) band ensuring enough intensity. For the same reason it is necessary to accept a wide and divergent beam instead of a "single ray" and thus the exact value of the asymmetry parameter ΔJ depends on the path within the beam.

In order to proceed with the analysis of the salient properties of a NSE signal in terms of analytic expressions we resort to Gaussian approximations for the incoming wavelength distribution

$$w(\lambda - \lambda_0) = \frac{1}{\Lambda\sqrt{\pi}} \exp\left[-\left(\frac{\lambda - \lambda_0}{2\Lambda}\right)^2\right] \tag{7}$$

this distribution is quite realistic for reactor based instruments with a wavelength selector even if it deviates slightly from the more triangular transmission function of a neutron velocity selector.

For the distribution of field integral differences within the beam we also use a Gaussian approximation

$$W(\delta - \Delta J) = \frac{1}{\Sigma\sqrt{\pi}} \exp\left[-\left(\frac{\delta - \Delta J}{2\Sigma}\right)^2\right]. \tag{8}$$

Here a Gaussian shape can only be considered as a generic description coarsely approximating some distribution with width corresponding to Σ.

However, these Gaussian approximations allow to derive analytical expressions for the NSE signal.

With that the detector signal as function of the spectrometer control variables $[J, \delta, \lambda_0]$, i.e. field integral (proportional to the main solenoid current), asymmetry (proportional to the phase coil current) and wavelength (at TOF instruments a function of the neutron arrival time:

$$I_{\text{Det}}[J, \delta, \lambda_0] = \frac{1}{2} \int \mathcal{S}_\lambda d\lambda \tag{9}$$

with

$$\mathcal{S}_\lambda = S(Q) \pm \int W(\delta - \Delta J) \cos\left(\Delta J \gamma_n \frac{m_n}{h} \lambda\right) \left\{ \int \cos\left(J\lambda^3 \gamma_n \frac{m_n^2}{2\pi h^2} \omega\right) S(Q,\omega) d\omega \right\} d\Delta J \tag{10}$$

Performing the integration over the field integral inhomogeneity distribution yields

$$\mathcal{S}_\lambda = S(Q) \pm \underbrace{\eta \exp\left(-\left[\Sigma \gamma_n \frac{m_n}{h} \lambda\right]^2\right)}_{\mathcal{R}} \cos\left(\delta \gamma_n \frac{m_n}{h} \lambda\right) \int \cos\left(\underbrace{J\lambda^3 \gamma_n \frac{m_n^2}{2\pi h^2}}_{t} \omega\right) S(Q,\omega) d\omega. \tag{11}$$

This yields the expression showing the salient features of the so-called resolution function $\mathcal{R}$, where we introduced an additional factor $\eta \simeq< 1$, which accounts for non-idealities of the polarisation analysis. Note that at high resolution $\Sigma \propto J$. The prefactor to ω in the second term of Eq. 11 corresponding to a cosine Fourier transform of the scattering function reveals the origin of the expression for the Fourier-time t.

1.1.1. Lambda integration The still missing integration over the wavelength distribution involves the implicit λ-dependence of the scattering function through its Q-dependence ($Q = 4\pi/\lambda \sin(\theta/2) = c_q/\lambda$). With the abbreviation $b = J\lambda^3\gamma_n(m_n/h)^2/2\pi$ we write $t = b\lambda^3$ and $t_0 = b\lambda_0^3$, with that the expression in the cosine Fourier integral in Eq. 10 can be written as a Taylor expansion around λ_0

$$S(Q,t) = S(Q_0,t_0) + \frac{(\lambda-\lambda_0)}{\lambda_0}S_1 + \frac{(\lambda-\lambda_0)^2}{\lambda_0^2}\frac{1}{2}S_2 + \frac{(\lambda-\lambda_0)^3}{\lambda_0^3}\frac{1}{6}S_3 + \cdots \tag{12}$$

with

$$S_1 = \left[\left\{-Q\frac{d}{dQ} + 3t\frac{d}{dt}\right\}S(Q,t)\right]_{Q_0,t_0} \tag{13}$$

$$S_2 = \left[\left\{Q^2\frac{d^2}{dQ^2} + 2Q\frac{d}{dQ} + 9t^2\frac{d^2}{dt^2} + 6t\frac{d}{dt}6Qt\frac{d^2}{dQdt}\right\}S(Q,t)\right]_{Q_0,t_0} \tag{14}$$

The λ integration then yields

$$I \propto \left[\langle S(Q_0)\rangle \pm \underbrace{\eta\frac{1}{A}e^{-\frac{(\Sigma\lambda_0\gamma_n m_n)^2}{(hA)^2}}}_{\mathcal{R}} \times \underbrace{e^{-\frac{(\Lambda\delta\gamma_n m_n)^2}{(hA)^2}}\cos\left(\frac{\delta\gamma_n m_n\lambda_0}{hA^2}\right)}_{\mathcal{E}} S(Q_0,t_0) + ...\right] \tag{15}$$

where we can identify the resolution $\mathcal{R}$ and the echo-shape $\mathcal{E} = \mathcal{E}_e(\delta\cdots)\times\cos(\delta\cdots)$ as parts of the prefactor to the intermediate scattering function. $\langle S(Q_0)\rangle$ denotes the wavelength averaged "static" scattering function. The factor A is a result of the wavelength dependence of the resolution function

$$A = 1\ +\ 4\Sigma^2\Lambda^2\gamma_n^2(m_n/h)^2 \tag{16}$$

For practical purposes $A \simeq 1$, which can be verified by inserting typical values for the wavelength width parameter $\Lambda = \Delta\lambda_{\mathrm{FWHM}}/(4\sqrt{\ln 2})$, $\lambda_0 = 1\,\mathrm{nm}$, FWHM width 10% and the inhomogeneity parameter $\Sigma = 2\times 10^{-6}\,\mathrm{Tm}$ yields $A = 1.0015$. The present analytical form and appearance of A can only be given in this simple form if the Gaussian approximations for w and W are assumed, for other shapes of w no analytic form could be found. Nevertheless, the Gaussian approximation exercise allows for the estimation of the magnitude of the effect and assures us that for the normal range of NSE parameters A is virtually 1. **In the DrSpine implementation $A = 1$.**

Finally, with the abbreviations $g = \gamma_n m_n/h$, $\psi^2 = [(\Sigma\lambda_0)^2 + (\Lambda\delta)^2](g/A)^2$ and $\Phi = \delta\lambda_0 g/A^2$ the next order of the λ can be written as

$$I \propto \left[\langle S(Q_0)\rangle \pm \frac{\eta}{A}e^{-\psi^2}\left\{\cos(\phi)S_0 - \frac{\Lambda^2}{\lambda_0^2}\frac{2}{A}\left([A^2\phi\sin(\phi) + 2(\Sigma\lambda_0 g)^2\cos(\phi)]S_1 - \frac{\cos(\phi)}{2A^2}S_2\right) + \mathcal{O}(\frac{\Lambda^4}{\lambda_0^4})\right\}\right] \tag{17}$$

For a 10% selector $\frac{\Lambda^2}{\lambda_0^2}$ is about 10^{-3} but S_1 and S_2 contain derivatives of the scattering function that may become large. However, for polymer systems $S(Q,t)$ is sufficiently smooth to result in negligible higher order contributions in Eq. 17. For intermediate function with sharper peaks these contributions can become larger.

Since at a TOF instrument (SNS) the effective width of the used λ-bins can be made very small these effects are suppressed even further compared to a selector instrument.

1.1.2. Echo shape envelope functions for selector instruments

Gaussian

$$\mathcal{E}_e(\delta) = \exp\left(-\frac{(\delta\gamma_n m_n)^2}{h^2}\frac{\Delta\lambda_{\mathrm{FWHM}}}{16\ln 2}^2\right) \tag{18}$$

Triangular

$$\begin{aligned}\mathcal{E}_e(\delta) &= 2\,\frac{1-\cos(z)}{z^2}\\ &\quad \text{with}\\ &\quad z = -\frac{(\delta\gamma_n m_n)}{h}\Delta\lambda_{\mathrm{FWHM}}\end{aligned} \tag{19}$$

Triangular (smoothed edges)

$$\begin{aligned}\mathcal{E}_e(\delta) &= 2\,\frac{1-\cos(z)}{z^2}\\ &\times\ \exp\left(-\frac{(\delta\gamma_n m_n)^2}{h^2}\delta\lambda^2\right)\end{aligned} \tag{20}$$

with a smoothing parameter $\delta\lambda << \Delta\lambda$.

2. Combination rule derivation

The data combination rule given in Eq. 19 of the main paper may easily be derived. The problem is basically that of combining two experimental results s_1 and s_2, both with experimental errors δs_1 and δs_2, such that the combined results has the lowest possible error. The optimisation pertains the prefactors α and $1 - \alpha$ used for the linear combination $s_{12} = \alpha s_1 + (1 - \alpha) s_2$. In that case the squared error of the result is

$$\delta s_{12}^2 = (\alpha \delta s_1)^2 + ([1 - \alpha] \delta s_2)^2 \tag{21}$$

and the minimum of Eq. 21 with respect to α corresponds to the optimum combination, i.e.

$$\alpha = \frac{\delta s_2^2}{\delta s_1^2 + \delta s_2^2}. \tag{22}$$

Inserting Eq. 22 into the expression for s_{12} it immediately yields

$$\begin{aligned} s_{12} &= \frac{\delta s_2^2\, s_1 + \delta s_1^2 s_2}{\delta s_1^2 + \delta s_2^2} \\ &= \frac{s_1/\delta s_1^2 + s_2/\delta s_2^2}{1/\delta s_1^2 + 1/\delta s_2^2}. \end{aligned} \tag{23}$$

The resulting error immediately follows from inserting the minimum α into Eq. 21 which – after trivial algebraic rearrangements – equals Eq. 20 of the main paper. As indicated in the main paper the case for more than two terms in the combination immediately follows by iterating the latest combination step.

3. Explanations pertaining the example report

report20670_707 stems from the evaluation of an experiment performed at the reactor instrument J-NSE in Garching. The name is derived from the run number of the leading (first) sample run and the following 707 is an automatic sequence number counting the created reports.

The first table summarizes all loaded raw-data files with the corresponding spectrometer settings etc.. Since this is a reactor experiment the wavelength frame λ_1, n contains a single nominal wavelength, 8Å. Then the wavelength width is read from the selector parameters contained in each raw data file. The consistency of wavelength and wavelength band width can immediately be judged by the plots show e.g. in figure 1 of the report. The only parameters to fit each of these curves to the counts in the corresponding pixel (symbols) are: δ_0, the location of the exact phase symmetry, the echo amplitude a and average level b.

3.1. Parameters

The data reduction in DrSpine can be controlled by a number of parameters pertaining pre-binning, post-histogramming and controls for acceptance/rejection of pixels. Once optimized for one instrument (and possibly classes of experiments, e.g. low Q vs high Q, coherent vs incoherent) it will rarely be necessary to adjust them further. This pertains the values listed in table 2 of the report. In order to expose all aspects of the evaluation they are explicitly set in lines 11 to 22 of the macro shown at the end of the report.

Besides those the detector pixel prebinning and, for TOF instruments, the prebinning of time channels can be specified. These are applied in the data reading step.

The user can also adjust some parameters pertaining the fitting of the echo, like for example the largest acceptable chi-square, the accepted tolerance or the smallest echo amplitude.

Next, the instruction *match* in the macro finds the corresponding data sets (reference, sample, background, *etc.*), and the *fit* command performs the actual echo signal fitting for all pixels and TOF bins. Starting with the best (central) pixels, the program determines the symmetry phase automatically for each pixel, which are then used in

the subsequent steps. In case of small magnetic disturbances and sufficient echo signal in the sample data, the user has the option to apply a global phase offset to the sample data (shifting the reference phase table as a whole) by selecting the option *fit sam offset*. This phase offset comprises all data from the entire detector area.

At last the user may choose to personalize the number of binning in Q and in the Fourier time. The final histogramming serves to collect this information into a selected grid of (Q, t) boxes. There one may explore the trade-offs between the degree of division in either Q, t or both versus statistics, i.e errors of each data point. Due to the application of proper weighting in determining the best effective Q and t values for each histogram box, even coarse binning yields reliable Q, t tables. Histogramming boxes that remain empty after the collection phase are ignored when preparing the output tables. This feature is employed in the example (800 time bins) to make sure that each Fourier time setting of the experiment gets a unique time slot assigned. For the continuous distribution of Fourier times encountered in an TOF-NSE (SNS) experiment one rather uses a t-binning into a few (typically $5 \cdots 30$) time slots equally distributed on a log-scale over the interval covering a range from minimum to maximum time compatible with the extremes of field integral settings and wavelength frames used in the experiment. As is done in the present example report a standard list covering settings from a range of possible divisions is created, enabling a posteriori selection.

As already mentioned in the main text, the final report provides, besides the curves for the intermediate scattering function in a linear and in logarithmic plot, also the possibility of choosing among some basic fitting functions that allow to extract first information on, e.g., the diffusion coefficient. A text (ASCII) file containing the $S(Q, \tau)$ points vs. τ data is automatically generated.
